# Early prediction and longitudinal modeling of preeclampsia from multiomics

**DOI:** 10.1016/j.patter.2022.100655

**Published:** 2022-12-09

**Authors:** Ivana Marić, Kévin Contrepois, Mira N. Moufarrej, Ina A. Stelzer, Dorien Feyaerts, Xiaoyuan Han, Andy Tang, Natalie Stanley, Ronald J. Wong, Gavin M. Traber, Mathew Ellenberger, Alan L. Chang, Ramin Fallahzadeh, Huda Nassar, Martin Becker, Maria Xenochristou, Camilo Espinosa, Davide De Francesco, Mohammad S. Ghaemi, Elizabeth K. Costello, Anthony Culos, Xuefeng B. Ling, Karl G. Sylvester, Gary L. Darmstadt, Virginia D. Winn, Gary M. Shaw, David A. Relman, Stephen R. Quake, Martin S. Angst, Michael P. Snyder, David K. Stevenson, Brice Gaudilliere, Nima Aghaeepour

**Affiliations:** 1 Department of Pediatrics, Division of Neonatal and Developmental Medicine, Stanford University School of Medicine, Stanford, CA 94305, USA; 2 Department of Genetics, Stanford University School of Medicine, Stanford, CA 94305, USA; 3 Department of Anesthesiology, Perioperative and Pain Medicine, Stanford University School of Medicine, Stanford, CA 94305, USA; 4 Department of Obstetrics and Gynecology, Stanford University School of Medicine, Stanford, CA 94305, USA; 5 Departments of Bioengineering and Applied Physics, Stanford University and Chan Zuckerberg Biohub, Stanford, CA 94305, USA; 6 Department of Biomedical Data Science, Stanford University, Stanford, CA 94305, USA; 7 Department of Surgery, Stanford University School of Medicine, Stanford, CA 94305, USA; 8 University of the Pacific, Arthur A. Dugoni School of Dentistry, San Francisco, CA 94103, USA; 9 Departments of Medicine, and of Microbiology & Immunology, Stanford University School of Medicine, Stanford, CA 94305, USA; 10 Digital Technologies Research Centre, National Research Council Canada, Toronto, Canada; 11 Infectious Diseases Section, Veterans Affairs Palo Alto Health Care System, Palo Alto, CA 94304, USA

**Keywords:** preeclampsia, machine learning, predictive modeling, multiomics, biomarkers

## Abstract

Preeclampsia is a complex disease of pregnancy whose physiopathology remains unclear. We developed machine-learning models for early prediction of preeclampsia (first 16 weeks of pregnancy) and over gestation by analyzing six omics datasets from a longitudinal cohort of pregnant women. For early pregnancy, a prediction model using nine urine metabolites had the highest accuracy and was validated on an independent cohort (area under the receiver-operating characteristic curve [AUC] = 0.88, 95% confidence interval [CI] [0.76, 0.99] cross-validated; AUC = 0.83, 95% CI [0.62,1] validated). Univariate analysis demonstrated statistical significance of identified metabolites. An integrated multiomics model further improved accuracy (AUC = 0.94). Several biological pathways were identified including tryptophan, caffeine, and arachidonic acid metabolisms. Integration with immune cytometry data suggested novel associations between immune and proteomic dynamics. While further validation in a larger population is necessary, these encouraging results can serve as a basis for a simple, early diagnostic test for preeclampsia.

## Highlights

• Machine-learning models for prediction of preeclampsia were developed
• Six omics datasets from a longitudinal cohort of pregnant women were analyzed
• Prediction models from urine metabolome and from proteome had the best accuracy
• The prediction model from urine metabolites was validated on an independent cohort

## The bigger picture

The World Health Organization estimates that more than 800 women worldwide die from pregnancy-related causes every day. One of the main causes is a hypertensive disorder, preeclampsia, for which the only treatment is to deliver, often too early. Preeclampsia affects 3%–5% of pregnancies in the United States and up to 8% globally. Machine-learning analyses of high-dimensional multiomics data could potentially capture complex dynamics involved in the preeclampsia pathogenesis. We developed machine-learning models for early prediction of preeclampsia (first 16 weeks of pregnancy) and over gestation by analyzing six omics datasets from a longitudinal cohort of pregnant women. A prediction model using nine urine metabolites had high accuracy and was validated on an independent cohort. While encouraging, our results need to be validated on a larger cohort. If generalizable, our findings could lead to a simple prediction test for use in both developed and developing parts of the world.

## Teaser

Preeclampsia is one of the main complications of pregnancy, posing risk both to the mother and the baby. We developed machine-learning models for early prediction of preeclampsia (first 16 weeks of pregnancy) and over gestation by analyzing six omics datasets from a longitudinal cohort of pregnant women. If further validated, our findings could lead to a simple prediction test for use in both developed and developing parts of the world.

## Introduction

The World Health Organization estimates that more than 800 women worldwide die from pregnancy-related causes every day, with the highest rates of maternal mortality and morbidity in low-income countries.^1^ One of the main causes is a hypertensive disorder of pregnancy, preeclampsia, for which the only treatment is to deliver, often too early. Preeclampsia affects 3%–5% of pregnancies in the United States and up to 8% of all pregnancies globally,^1^ and accounts for 10%–15% of maternal deaths^2^ and 15%–20% of preterm births.^3^

The pathophysiology of preeclampsia is complex and is thought to be caused in part by abnormal placentation as well as a woman’s genetic and immunologic predisposition.^4^ It is believed that abnormal placentation leads to a maternal inflammatory response.^4^ Placental ischemia, oxidative stress, and the presence of a maternal angiogenic imbalance are all characteristics of preeclampsia,^5^^,^^6^ leading to endothelial and end-organ damage, and in some cases to stroke and even death.

Specific biological processes involved in the development of preeclampsia are not yet completely understood. Early prediction of preeclampsia has remained a clinical challenge, owing to incompletely understood causes, various risk factors, and likely multiple pathogenic phenotypes of preeclampsia.^7^^,^^8^ The recent availability of high-throughput omics (e.g., genome, transcriptome, proteome, and metabolome) assays, where each can be performed on small sample volumes, has enabled joint analyses of the high-dimensional multidomain or “multiomics” data measured from the same biological sample.^4^^,^^9^^,^^10^ An integrated analysis may capture complex dynamics involved in the preeclampsia which could ultimately lead to novel therapeutic interventions. Furthermore, applying machine-learning methods capable of extracting the most predictive features from high-dimensional multiomics data could lead to more accurate predictive models, discovery of biomarkers, and improved early detection of women at risk for developing preeclampsia.

In this study, we performed a multiomics analysis of the transcriptome, proteome, metabolome, lipidome, and microbiome from a coordinated set of biospecimens collected longitudinally from normotensive and preeclamptic pregnant women; we then integrated immune system mass spectrometry features that were available for a subset of the women; and finally, we combined the multiomics data with the available clinical/demographics data and performed a joint analysis. Our goals were to: (1) build an early prediction model of preeclampsia; (2) develop a simple and interpretable predictive model based on a small number of biomarkers that can lead to the development of a diagnostic test; (3) compare prediction capabilities of different omics sets; (4) build an integrated multiomics predictive model of preeclampsia to identify a signature of preeclampsia; and (5) gain insights into pathways involved in the pathogenesis of preeclampsia.

## Results

### Study design and multiomics data collection

**Figure 1:**
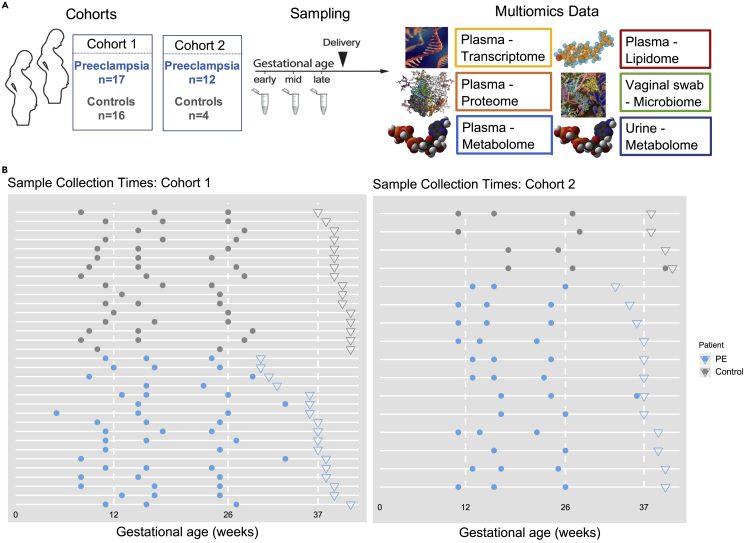
Overview of the study (A) Two independent cohorts were analyzed using six different omics. (B) Sample collection timeline for plasma in our discovery and validation cohorts. Circles indicate pre-delivery sample collection times, and inverted triangles indicate delivery dates for individual women (one per horizontal line).

Our prospective study included 33 women in the discovery cohort (17 preeclamptic, 16 normotensive) and 16 women in the validation cohort (12 preeclamptic, 4 normotensive) (Figure 1A). The validation cohort was used to validate the metabolomics results. Among the preeclamptic women in the discovery cohort, severe and mild preeclampsia were observed in 10 and 7 women, respectively; early- and late-onset preeclampsia were observed in 5 and 12 women, respectively (Table S2). Maternal characteristics, demographics, and gestational ages at delivery are shown in Table S1. Both in discovery and validation cohorts there was a higher prevalence of chronic hypertension, high body mass index (BMI), and twin pregnancies—all known risks for preeclampsia—among preeclamptic compared with normotensive women (Table S1).

Blood, urine, and vaginal swabs were collected longitudinally at two or three time points during pregnancy: early, mid, and late (Figure 1). Across the gestation, we found no significant difference in sampling time between preeclamptic and normotensive groups (p > 0.74 first sample, p > 0.6 second sample, and p > 0.3 third sample; Wilcoxon rank-sum test). These samples were used for measurements of six omics assays: cell-free RNA (cfRNA)/transcriptome (plasma), proteome (plasma), metabolome (plasma and urine), lipidome (plasma), and microbiome (vaginal swab). In addition, immune-system-wide mass cytometry measurements of single cells were obtained on a subset of 19 women from the same cohorts (18 women from the discovery cohort and one woman from the validation cohort). The number of measurements differed markedly among omics datasets, with transcriptome containing the highest number of measurements (Figure S1A). In contrast, the number of principal components explaining 90% of the variance, which quantifies the internal correlation of a dataset, exhibited a smaller difference among datasets (Figure S1B). Thus, although the amount of data varied several orders of magnitude among datasets, their numbers of principal components were much more similar.

### Prediction of preeclampsia in early pregnancy

**Figure 2:**
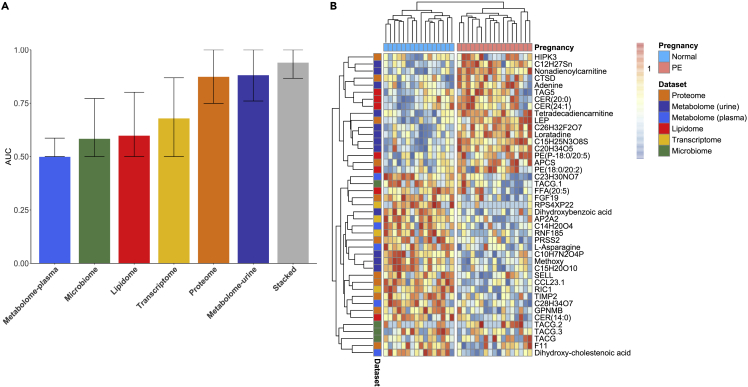
Prediction models in early pregnancy Samples obtained in the first 16 weeks of pregnancy were used. (A) Performance comparison of EN models derived from different omics in terms of the AUC. The integrated (stacked) model utilizing stacked regression exhibited the highest accuracy (AUC = 0.94, 95% CI [0.86, 1]). Among omics sets the urine metabolomic model (AUC = 0.88, 95% CI [0.76, 0.99]) and plasma proteome (AUC = 0.87, 95% CI of [0.75, 0.99]) performed best. (B) Heatmap of ranked values of features identified by EN, perfectly distinguishing preeclamptic from normotensive women.

From a clinical perspective, early prediction of preeclampsia, i.e., within the first 16 weeks of gestation, is of critical importance, as it would enable: early treatment of high-risk women (e.g., with low-dose aspirin^11^); closer monitoring of high-risk pregnancies; and the enrichment of preemptive interventional studies in women at risk for developing preeclampsia.^12^ Identifying a small number of specific biomarkers that are predictive of preeclampsia early in pregnancy could ultimately facilitate the development of a simple and affordable diagnostic test for both high-income and low- and middle-income countries. To this end, we developed an early prediction model for preeclampsia using only samples collected from each omics dataset during the first 16 weeks of pregnancy. To agnostically examine all the measurements in our high-dimensional data, we used Elastic Net (EN), a regularized regression machine-learning method (see experimental procedures). EN was chosen for its ability to extract, from high-dimensional data, a handful of the most predictive features that can predict an outcome with high accuracy.^13^ Performance was evaluated using the leave-one-out cross-validation method. Comparison of predictors demonstrated the highest performance of the urine metabolome predictive model (area under the receiver-operating characteristics curve [AUC] = 0.88, 95% confidence interval [CI] [0.76, 0.99]) followed by the proteome model (AUC = 0.87, 95% CI [0.75, 0.99]) and cfRNA models (AUC = 0.68, 95% CI [0.49, 0.87]) outperforming models from other omics (Figure 2A). Top identified proteins and genes are shown in Figure S7.

The heatmap of rank values of features selected by EN from all omics is shown in Figure 2B. Hierarchical clustering was used to separate preeclamptic from normotensive pregnancies.

**Figure 3:**
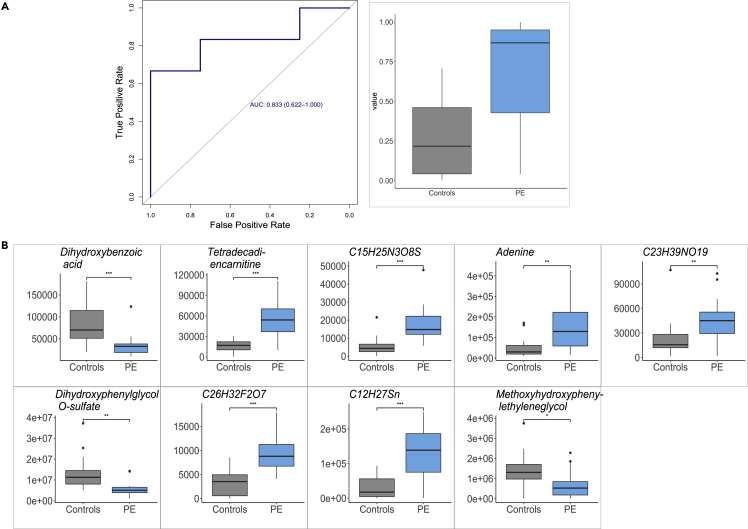
Urine metabolome prediction model using nine metabolites sampled early in gestation validates on the validation cohort Samples obtained in the first 16 weeks of pregnancy are used. (A) AUC = 0.83, 95% CI [0.62, 1] and prediction values (scores) obtained by EN for preeclamptic (PE) and normotensive women. (B) Metabolites identified by EN as biomarkers of preeclampsia. y axis shows the value in early pregnancy stratified by normotensive (gray) and preeclamptic (light blue) pregnancies. p values obtained using Wilcoxon signed-rank univariate analysis show statistical significance of each protein (∗p ≤ 0.05, ∗∗p ≤ 0.01, ∗∗∗p ≤ 0.001).

We next focused on our top-performing model, which was trained from the urine metabolome dataset and consisted of nine metabolites, and evaluated its performance in the validation cohort. The model validated maintaining high performance with an AUC of 0.83 (95% CI [0.62, 1.0]) (Figure 3A), confirming identified metabolites (Figure 3B) as biomarkers of preeclampsia. Furthermore, p values obtained using a separate univariate analysis demonstrated the statistical significance of each of the identified metabolites (Figure 3B). The metabolites identified by EN as the biomarkers were dihydroxybenzoic acid, tetradecadiencarnitine, adenine, dihydroxyphenylglycol *O*-sulfate, methoxyhydroxyphenylethyleneglycol, and four uncharacterized molecules (C_23_H_39_NO_19_, C_26_H_32_F_2_O_7_, C_15_H_25_N_3_O_8_S, C_12_H_27_Sn) (Figure 3B).

### Machine-learning modeling of preeclampsia over gestation

**Figure 4:**
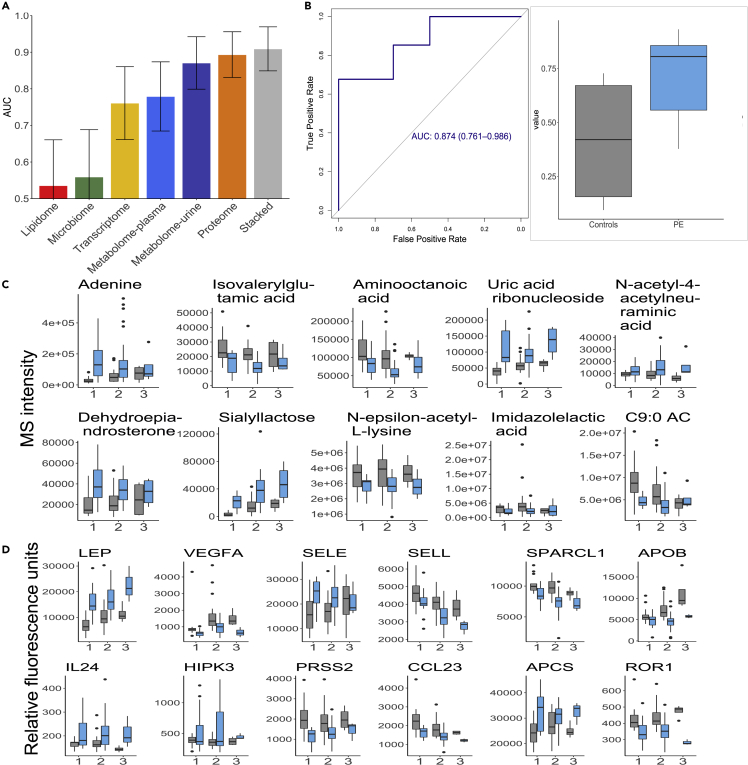
An integrated multiomics machine model outperforms single omics models for preeclampsia (A) Cross-validated performance of machine-learning models in terms of the AUC is shown on the y axis. Each model was obtained using all available samples over gestation. The integrated (stacked) model utilizing stacked regression exhibited the highest accuracy (AUC = 0.91, 95% CI [0.85, 0.97]). Both proteome and metabolome (urine) had high prediction performance (AUC = 0.89, 95% CI [0.83, 0.95] proteome; AUC = 0.87, 95% CI [0.80, 0.94] urine metabolome). (B) Urine metabolome prediction model using ten metabolites sampled over gestation validates on the validation cohort. AUC = 0.874, 95% CI [0.76, 0.99], and prediction values (scores) obtained by EN for normotensive and preeclamptic (PE) women. (C) Metabolites identified by EN as biomarkers of preeclampsia over gestation. y axis shows values stratified by normotensive (gray) and preeclamptic (light blue) pregnancies. p values obtained using linear mixed-effects univariate analysis show statistical significance of each metabolite (p < 0.05). (D) Proteins identified by EN as biomarkers of preeclampsia over gestation. y axis shows a protein value stratified by normotensive (gray) and preeclamptic (light blue) pregnancies. p values obtained using linear mixed-effects univariate analysis show statistical significance of each metabolite (p < 0.05).

We next analyzed longitudinal data that included all samples taken during pregnancy (Figure 1) in order to capture the changes that occur due to preeclampsia during pregnancy and possibly gain insights into the development of preeclampsia. As in the early pregnancy analysis, multivariate models of preeclampsia were trained for each omics using EN (see experimental procedures). To investigate whether a joint analysis of different omics can offer further gains, predictions from separate omics models were integrated in a joint model using stacked regression (see experimental procedures). The performance of all models was evaluated using the leave-one-out cross-validation method. The integrated model exhibited the highest prediction accuracy (AUC = 0.91, 95% CI [0.85, 0.97]), outperforming predictions from each separate model in terms of the point estimate (Figure 4A). EN models from the proteome and urine metabolome exhibited high performance (AUC = 0.89, 95% CI [0.83, 0.95]; AUC = 0.87, 95% CI [0.80, 0.94], respectively) outperforming other omics data, the same trend we observed in the early pregnancy performance. As before, the urine metabolite model was validated in an independent cohort, with an AUC of 0.87 (95% CI [0.76, 0.99]) (Figure 4B), confirming identified metabolites as true biomarkers of preeclampsia.

Top urine metabolites included adenine, isovalerylglutamic acid, uric acid ribonucleoside, 1,5-anhydroglucitol, dehydroepiandrosterone, sialyllactose, *N*^ε^-acetyl-L-lysine, and nonanoylcarnitine. p values obtained using a separate univariate analysis show statistical significance of each of the identified metabolites (Figure 4C). One of the identified metabolites, uric acid ribonucleoside, is an end product in the same pathway as uric acid, whose increased concentration is typical of preeclampsia.^14^ As an end product, uric acid ribonucleoside is more likely to be a sensitive biomarker. Interestingly, the uric acid levels in our data did not discriminate between controls and preeclamptic patients.

A model using top-scoring plasma proteins achieved an AUC of 0.83 (95% CI [0.73, 0.92]) (Figure 4A). The most predictive plasma proteins selected by EN included leptin (LEP), vascular endothelial growth factor A (VEGFA), L-selectin (SELL), E-selectin (SELE), interleukin-24 (IL-24), IL-22, tyrosine-protein kinase transmembrane receptor (ROR1), C-X-C motif chemokine ligand 10 (CXCL10), and SPARC-like 1 (SPARCL1) (Figure 4D), thereby confirming some of the established or indicated proteins associated with preeclampsia^15^^,^^16^^,^^17^ (see Table S4 and discussion for further details), as well as establishing new associations. Also in this case, p values obtained using univariate analysis show that all proteins chosen by EN are statistically significant (Figure 4D).

We point out that, as expected, EN models varied slightly owing to variability of the chosen training set in each leave-one-out cross-validation step^18^ and therefore, the features chosen by EN varied slightly across cross-validations. We recorded the frequency of occurrence for every feature across all cross-validation steps (shown for the proteome model in Figure S2). Having high frequency of occurrence indicates that the feature is relevant for all or a majority of samples, i.e., it is more stable.^18^

Because both in discovery and validation cohorts the prevalence of known preeclampsia risks including chronic hypertension, high BMI, and twin pregnancies was higher among preeclamptic compared with normotensive women (Table S1), we next investigated whether our multiomics model captures mostly these differences. We calculated Spearman correlation between prediction model scores and clinical variables (Figure S3). The first-trimester blood pressure was also included in the analysis. The highest correlation between the model and clinical variables, and the only one that was statistically significant, was found to be between BMI and the model (p < 0.0086). However, even in this case the BMI did not fully correlate with the model (Spearman correlation = 0.63) confirming that our model does not just capture differences in BMI to distinguish between preeclamptic and normotensive women. We observed a low correlation between the model (row labeled “prediction” in Figure S3) with other clinical variables, indicating that the omics model is not just describing differences in the available clinical/demographic characteristics of women but is capturing biological differences.

**Figure 5:**
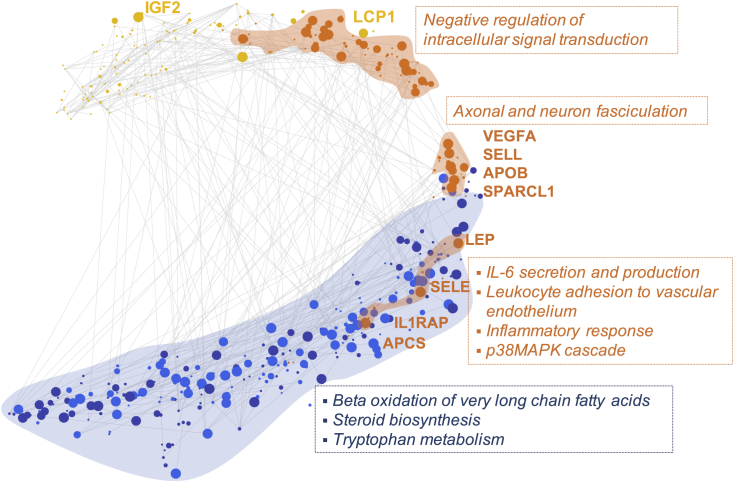
Visualization of predictive features of the transcriptome (yellow), proteome (orange), urine metabolome (dark blue), and plasma metabolome (light blue) Features obtained using all available samples over gestation. Vertices represent features selected by EN laid out using t-SNE. Edges are drawn between features with Spearman correlation >0.55 clearly illustrating high correlations between different omics sets. Size of each node is proportional to the frequency at which it was chosen in prediction models during cross-validation. High frequency of occurrence indicates that a feature is relevant for all or majority of patients, resulting in a more stable model.

The correlation network of all features chosen by EN models for each of the omics sets was plotted using t-distributed stochastic neighbor embedding (t-SNE), revealing multiomics interaction of analytes associated with preeclampsia (Figure 5). Edges between features indicate a Spearman correlation >0.55. As expected, features from one omics set tend to group together, with higher correlation among them. In addition, we observe that correlation exists among different omics datasets. Exploiting these correlations may be a plausible explanation as to why the integrated prediction model outperforms individual models as shown in Figure 3. Pathway enrichment analysis revealed that three protein clusters observed in the plot were associated with different pathways: (1) pathways related to immune response (bottom cluster); (2) pathways related to neurodevelopment (middle cluster); and; (3) pathways related to intracellular signal transduction (top cluster) (Figure 5). Three metabolic pathways were enriched: (1) steroid biosynthesis; (2) tryptophan metabolism, as in the case of univariate analysis (Figure 7); and (3) β-oxidation of very long chain fatty acids whose role in preeclampsia has been previously observed.

Finally, we compared the most predictive features as identified by EN in early pregnancy versus during gestation in terms of their significance (−log_10_(p values)). Plasma proteins are shown in Figure S5A and urine metabolites in Figure S5B. We observe that a large number of proteins identified by EN stay statistically significant in both cases, including LEP, SELL, CCL23, ROR1, IL1RAP, SELL, SELE, VEGFA, IGFBP1, and SPARCL1 (Figure S4A). Some of the proteins are significant over gestation but not early in pregnancy (e.g., APOB) possibly due to a smaller number of samples. A similar trend is observed for urine metabolites (Figure S4B). Also noteworthy is that because EN uses sparsity, a feature that is associated with preeclampsia may be excluded from the final model if that model already includes another feature highly correlated with the original one. This is especially true in scenarios with a large number of features and high-dimensional regime such as in our study. This effect is illustrated in Figure S5, showing the difference in chosen features for prediction models over gestation and in early pregnancy.

### Single-cell characterization of the immune system

**Figure 6:**
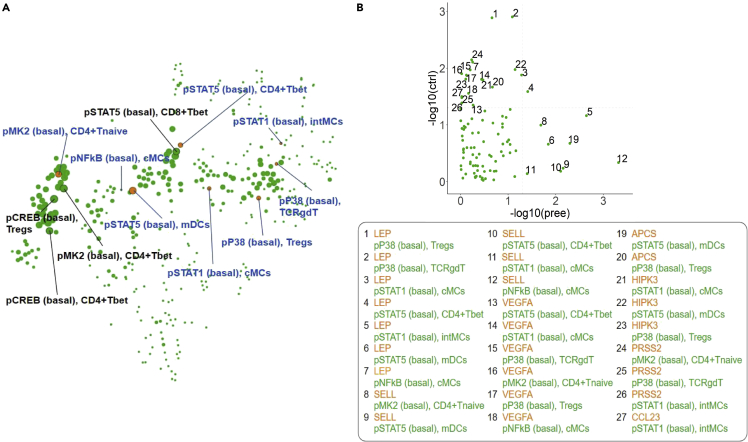
Correlation between predictive immune features and multiomics model (A) Previously identified predictive immune features strongly correlate with the multiomics predictive model. Visualization shows features most correlated with the prediction of the stacked model. Features shown in orange are the seven most predictive immunome features that also highly correlate with the multiomics predictive model. Size of each node is proportional to the −log_10_(p value) of Spearman correlation. (B) Comparison of p value of correlation for the top immune and top proteome features. Each node is a pair comprising an immune and a proteome feature.

Preeclampsia is strongly associated with inflammation and aberrant maternal immune system adaptations during pregnancy.^19^ To assess immunity—which is complementary to pathways covered by proteins and metabolites—and connect differential abundances of plasma proteins and urine metabolites in preeclamptic pregnancies to biological changes, immune-system-wide mass cytometry measurements of single cells obtained in a subset of the same patient cohort were integrated with our plasma proteome and urine metabolome prediction models, as these two models had the best accuracy. Immune cell dynamics between first- and second-trimester blood samples obtained from high-dimensional mass cytometry were previously used to develop a prediction model of preeclampsia.^20^ We found that seven (out of eight) of the immune features reported by Han et al.^20^ correlated highly with the prediction based on our integrated algorithm (Spearman correlation p < 0.05) (Figure 6A, highlighted in orange), confirming the predictive value of immune cell features as well as plasma proteins and urine metabolites. To investigate whether this correlation between predictive features was biologically meaningful, we focused on the correlations of feature behavior between the eight earlier reported predictive immune features (Figure 6A) and the top 12 most informative plasma proteome features (Figure 4D) across pregnancy (Figure 6B). LEP and SELL levels were particularly strongly correlated with the eight immune cell features (Figure 6B). Interestingly, basal pSTAT5 signaling in T helper 1 (Th1) cells (CD4^+^Tbet^+^), the top immune feature to distinguish control from preeclamptic pregnancies,^20^ correlated with LEP levels in both control and preeclamptic patients. Uniquely in preeclamptic cases, LEP levels were correlated with basal pSTAT1 signaling in intermediate myeloid cells (intMCs) (Spearman correlation p = 0.002) and basal STAT5 signaling in myeloid dendritic cells (mDCs) (Spearman correlation p = 0.01). Moreover, SELL levels were uniquely correlated with immune features in preeclamptic pregnancies and not with controls, i.e., correlated with basal pNFkB and pSTAT1 signaling in cMCs, basal pSTAT5 signaling in Th1 cells and mDCs, and basal pMAPKAPK2 signaling in naive CD4 T cells. Preeclamptic pregnancies were not characterized by—in other words, had potentially lost—concerted proteome/immune behavior, which was prominently observed in healthy pregnancies, i.e., correlations of leptin with basal pP38 signaling in T regulatory (Treg) and T cell receptor γδ (TCRγδ) cells. These correlations exemplify the biological connection between responsiveness of immune cells and its plasma environment.

### Relationship between clinical data and omics measurements

Clinical and demographics data contain maternal characteristics known to be associated with the risk of preeclampsia, e.g., preexisting hypertension, race, BMI, height, and gravida. We combined ten variables that were available in this dataset (Table S1) with the most predictive sets, (1) plasma proteome and (2) urine metabolome models, to better understand their mutual relationship. The ten clinical variables were included together with the top ten omics features, all combined in the single cross-validation step. Inclusion of clinical and demographics data improved the performance when combined with both the plasma proteome and the urine metabolome (urine metabolome AUC = 0.96, 95% CI [0.92, 0.99]; proteome AUC = 0.91, 95% CI [0.85, 0.97]) (Figure S6A). The most predictive clinical variables included maternal age, BMI, height, and preexisting hypertension. We observed several significant correlations (Spearman correlation p < 0.05) between clinical variables and plasma proteins/urine metabolites that were present only among preeclamptic women. These included: leptin with maternal BMI/weight, in agreement with existing literature;^21^ CCL23 with height; SELL with gravida (Figure S6B); maternal age with adenine—previously observed^22^—and maternal age with isovalerylglutamic acid (Figure S6C).

In addition, to compare the above performance with the performance of a baseline model, we trained EN only on the available maternal and pregnancy characteristics. The obtained model still had a good accuracy, AUC = 0.85, 95% CI [0.66, 1.0], but the performance, as expected, was lower compared with the combined model. The most predictive variables in the model were maternal hypertension, BMI, race, age, and number of babies in the current pregnancy. This is in line with results in our previous work that developed a machine-learning model for early prediction of preeclampsia from electronic health record data.^23^

### Univariate analysis of preeclampsia pathogenesis from multiomics measurements

We next present univariate analysis with the Benjamini-Hochberg procedure that identifies all features significantly associated with preeclampsia. We further used these features to perform pathway enrichment analysis.

#### Over the course of pregnancy

**Figure 7:**
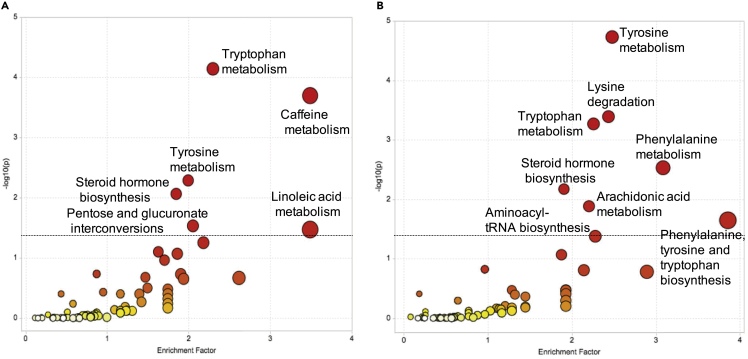
Identified enriched pathways from urine metabolome urine over gestation and in early pregnancy (A) Pathway enrichment analysis over gestation using metabolites from urine that were significant (FDR < 0.05, Wilcoxon signed-rank test with Benjamini-Hochberg procedure). Pathways shown above the dotted line were significant (p < 0.05). (B) Pathway enrichment analysis for early pregnancy using metabolites from urine that were significant (FDR < 0.05, linear mixed-effects model with Benjamini-Hochberg procedure). The color and the size of a circle are proportional to the −log(*p*) and pathway impact value, respectively, where *p* denotes a p value.

Changes over gestation of 1,215 metabolic features among 8,718 were significantly associated with preeclampsia outcome (false discovery rate [FDR] < 0.05, linear mixed-effects [LME] model with Benjamini-Hochberg procedure). Pathway enrichment analysis using these urine metabolites identified the following pathways (p < 0.05) (Figure 7A): (1) tryptophan metabolism; (2) caffeine metabolism; (3) tyrosine metabolism; (4) steroid hormone biosynthesis; (5) pentose and glucuronate interconversions; (6) linoleic acid metabolism. The steroid hormone biosynthesis pathway plays an important role in pregnancy progression.^24^ Both the steroid hormone biosynthesis pathway and the caffeine metabolism pathways and caffeine metabolites have previously been associated with pregnancy,^25^^,^^26^ and tryptophan metabolism with preeclampsia.^27^ Metabolites in the steroid hormone biosynthesis pathway and in the caffeine metabolism pathway present in the data with high level of significance are respectively shown in Figures S8C and S8D. The comparison between enrichment factors and statistical significance of pathways enriched over gestation versus in early pregnancy is shown in Figure S22.

Among 1,305 proteins, 437 had changes that were significantly associated with preeclampsia outcome over gestation (FDR < 0.05, LME model with Benjamini-Hochberg procedure). The top 64 proteins at significance level $\text{p}<5\times 10^{-4}$ (LME model) showed markedly different values between normotensive and preeclamptic women (Figure S9). Top proteins included IL-1 receptor accessory protein (IL1RAP) and SELL, both known to play a role in the immune response.^28^ Enriched pathways grouped into ten biological processes, the most prevalent being positive regulation of cellular processes (including biological, cellular, protein metabolic, immune system, and apoptotic processes among others) (46.4%) (Figure S10). In the cfRNA set, 306 features were significantly associated with preeclampsia outcome over gestation (FDR < 0.05, LME model with Benjamini-Hochberg procedure). Enriched pathways grouped into 11 biological processes, the most prevalent being RNA splicing (37.3%) (Figure S11). Top features included YOD1 (known to be related to developmental processes^29^), BIRC2, CEP63, and LCP1 (also previously implicated with preeclampsia^30^). A network of top proteome, transcriptome, and urine and plasma metabolome features is shown in Figure S12.

#### Early pregnancy

In early pregnancy, 497 out of 8,718 urine metabolic features had changes significantly associated with preeclampsia when compared with normotensive controls (FDR < 0.05, Wilcoxon signed-rank test with Benjamini-Hochberg procedure). Pathway enrichment analysis on these urine metabolites identified the following pathways (p < 0.05) (Figure 7B): (1) tyrosine metabolism; (2) lysine degradation; (3) tryptophan metabolism; (4) phenylalanine metabolism; (5) steroid hormone biosynthesis; (6) arachidonic acid metabolism; (7) phenylalanine, tyrosine, and tryptophan biosynthesis; (8) aminoacyl-tRNA biosynthesis. Arachidonic acid metabolism is a central regulator of the inflammatory response and has a known role in the pathogenesis of preeclampsia.^31^ Similarly, tryptophan metabolism has an important role in pregnancy, providing increased protein synthesis by the mother, fetal growth development; and serotonin for signaling pathways.^32^ Individual metabolites from these two pathways are shown in Figures S8A and S8B.

In the proteome set containing 1,305 proteins, three proteins—LEP, CCL23, and FAM3D—were significantly associated with preeclampsia outcome (FDR < 0.05, Wilcoxon signed-rank test with Benjamini-Hochberg procedure) identifying one significantly enriched pathway, negative regulation of glucagon secretion (Fisher’s exact test with Benjamini-Hochberg procedure, FDR < 0.05). The reason we did not adjust for covariates, specifically BMI—the only covariate with statistically significant correlation with the model predictions (Figure S3)—is that we wanted to capture the underlying biology including the mechanism under which the existing factors such as BMI are associated with preeclampsia. By adjusting for BMI, we would potentially remove pathways otherwise enriched and involved in preeclampsia.

#### Outlier analysis

We observed that a few women in our cohort were consistently misclassified by our prediction algorithm (Figure S13). A few normotensive control women resembled those with preeclampsia on a molecular level in some of the top predictive features across omics sets. Vice versa, there were some preeclamptic women whose top molecular features more closely resembled those of controls. Reexamination of the clinical charts revealed that one of the preeclamptic women, while clearly hypertensive, had proteinuria in the context of gross hematuria, obscuring whether proteinuria was related to preeclampsia. Therefore, she may have been misdiagnosed with preeclampsia but rather only had gestational hypertension. This highlights that the predictive model can pick up discrepancies within the clinical chart. For the other women whose clinical diagnosis held, this implies that their phenotypic features that classified them in either normotensive or preeclampsia group did not match their molecular phenotypes. Of interest, one preeclamptic woman, which the prediction classified as control, developed HELLP syndrome very late in gestation at 41 + 3 weeks. Therefore, if she had delivered closer to the due date she would have been considered a control. Thus, if others in the control group have a similar molecular phenotype, this may represent a late-onset preeclampsia related to placental aging in the post-term period.

## Discussion

Recent omics studies of preeclampsia typically included up to two omics datasets.^10^^,^^33^^,^^34^ Our study presents an integrated analysis of six high-throughput omics datasets obtained on the same biological sample, containing more than 50,000 measurements per sample. This multiomics analysis enabled uniform comparison of omics sets and revealed improved predictive ability for preeclampsia status relative to individual biological modalities, and indications of biological processes associated with the disease across multiple modalities. The first part of the analysis focused on early prediction of preeclampsia with the goal of comparing the six omics and identifying the best biomarkers. We then used a multiomics approach to integrate six omics datasets into one integrated (stacked) prediction model. The multiomics analysis demonstrated that the plasma protein and urine metabolome had the highest accuracy, both early in pregnancy (Figure 2A) and over gestation (Figure 4A). For that reason, we followed this with a more targeted analysis of plasma proteins and urine metabolites that were identified as having the highest accuracy. Ultimately, our goal is to develop a simple diagnostic test with high accuracy, and these two omics datasets were identified as most promising from our analysis.

One of the main strengths of our study is that, in our cohort, biological samples were not only collected longitudinally from each woman, but each individual sample was also simultaneously measured for proteome, transcriptome, metabolome, lipidome, and vaginal swab for microbiome, thereby providing a unique opportunity to systematically study changes attributable to preeclampsia over gestation, and compare the capability of each of these omics sets to predict and characterize preeclampsia. All 50,000 measurements were used in the prediction algorithm to agnostically identify the best biomarkers of preeclampsia.

Among our six omics, urine metabolomic and plasma proteomic datasets demonstrated the highest prediction accuracies, both over gestation and early in pregnancy. A prediction model using a small number of urine metabolites provided high accuracy over gestation (AUC = 0.88, cross-validated) and early in pregnancy (AUC = 0.875, cross-validated). The prediction model was validated on an independent cohort (AUC = 0.83 in early pregnancy; AUC = 0.87 over gestation), confirming identified metabolites as true biomarkers. Univariate analysis demonstrated the statistical significance of these biomarkers.

The EN prediction model with plasma proteins achieved AUC of 0.83 over gestation and of 0.88 in early pregnancy. Several of the proteins identified by our model as the most predictive have previously been well established as biomarkers of preeclampsia (VEGFA,^15^ LEP,^16^ SELL,^35^ CXCL10,^36^ ROR1,^37^ and IL1RAP^38^), further validating our results. Some of the identified proteins have been previously indicated in individual studies but have not yet been confirmed (IL-24,^17^ HIPK3,^39^ and SPARCL1^40^), and some have been identified for the first time (IL-22). While these biomarkers were previously examined in typically more targeted studies examining a single or small group of these proteins (e.g., IL-24^17^), our agnostic approach demonstrates that put together, their combined measurements result in an accurate model of preeclampsia. One of identified proteins in our model was VEGFA. Reduced levels of VEGFA have previously been described in preeclamptic pregnancies, owing to increased levels of placental soluble FMS-like tyrosine kinase-1 (sFLT-1), which validate our study.^15^^,^^41^^,^^42^ Among the other known biomarkers of preeclampsia—sFLT-1, pregnancy-associated plasma protein A (PAPP-A), placental growth factor (PIGF), and endoglin (ENG)—PIGF and PAPP-A were indeed significantly different between normotensive and preeclamptic women (Figure S15). The fact that ENG and sFLT-1 were not significant may be in part due to the small size of our cohort. In addition, we point out that sFLT-1 is a good predictor of preeclampsia later in pregnancy and once suggestive clinical features are observed,^43^ whereas the majority of our samples were taken before that point. Clinically, it is the sFLT-1/PIGF ratio that is used as a biomarker and not individual levels (Figure S14B). Throughout pregnancy PIGF levels are increasing until sFLT-1 levels start to increase, which is consistent with what is observed in our data (Figure S14) and suggests a variety of new hypotheses for testing. We did not include PP13 (galectin13) measurements, another known biomarker of preeclampsia.

Preeclampsia is accompanied by a dysregulated maternal immune adaptation to pregnancy, which is already detectable in early pregnancy.^19^^,^^20^ This aberrant signature was previously identified in women who developed preeclampsia later.^20^ Here we report that the intricate functional capacities of immune cells are coevolving with their environment throughout the course of pregnancy, showing that top informative immune feature levels are highly correlated with top informative plasma proteins. This interconnectedness supports both prediction approaches, confirming their individual usefulness while complementing the validity of each approach. The results highlight the known pathophysiology of preeclampsia and suggest novel associations between immunological and proteomic dynamics. In preeclamptic pregnancies, immune responses were uniquely correlated with levels of LEP and SELL.

LEP, known to be elevated in the plasma of preeclamptic women,^16^ is an immune regulatory hormone produced by adipose tissue and the placenta.^16^^,^^44^ LEP activates the JAK/STAT and MAPK pathways directly through binding to the leptin receptor expressed on leukocytes, and thereby modulates both innate and adaptive immune responses,^45^^,^^46^ including skewing of CD4 T cells toward Th1 polarization^47^ and inhibiting Treg proliferation.^46^ Accordingly, we observed that LEP levels in preeclamptic and control pregnancies correlated with STAT and MAPK pathway signaling both in innate and adaptive immune cells, suggesting that dysregulated leptin levels in preeclamptic pregnancies might contribute to the aberrant immune signature while, reciprocally, inflammation itself might enhance plasma leptin levels.^44^^,^^45^ Moreover, while in healthy pregnancies LEP levels correlated with p38 signaling in Treg and TCRγδ, this correlation was lost in preeclamptic pregnancies, suggesting that regulation of immune tolerance might be disrupted in preeclamptic pregnancies.

Furthermore, we reported decreased SELL levels in preeclamptic pregnancies that correlated with basal pSTAT, pNFkB, and pMAPKAP2 signaling in innate (mDC and cMC) and adaptive immune cells (Th1 and naive CD4 T cells). SELL is shed from leukocytes during activation and migration, and soluble L-selectin can be used as a surrogate marker for inflammation.^48^ Notably, a drop in soluble SELL levels is observed during sepsis.^49^ Previous studies reported conflicting results for circulating soluble SELL levels in preeclampsia,^35^^,^^50^^,^^51^ including low soluble SELL levels at 20 weeks of gestation, prior to onset of preeclampsia.^50^ Preeclampsia-associated enhanced ectodomain shedding of cell-adhesion molecules could be directly linked to changes in signaling responses in circulating immune cells by shedding-mediated activation of intracellular pathways.^48^ Alternatively, the correlation could reflect independent inflammatory mechanisms, as decreased levels of circulating SELL have been proposed to be due to its adsorption to luminal vascular ligands, which are upregulated by an activated endothelium, a feature of preeclampsia.^6^^,^^50^^,^^52^

The model from urine metabolites predicted preeclampsia with highest accuracy. Enrichment analysis identified discriminant biological pathways associated with preeclampsia when considering early and all time points. The steroid hormone biosynthesis pathway was significant (p < 0.05) in both models while arachidonic acid metabolism was significant in early pregnancy. Arachidonic acid is a precursor to a myriad of bioactive lipids including prostaglandins (PGs), prostacyclin, thromboxane, hydroperoxyeicosatetraenoic acid, leukotrienes, lipoxins, hypoxins, anandamide, and epoxyeicosatrienoic acids, which play key roles in inflammatory, vascular, and coagulation processes.^53^ As early as the 1960s the role of the eicosanoids in preeclampsia pathogenesis was proposed, and by the 1970s evidence supported that an increase in thromboxane (TXA2; produced by platelets) over prostacyclin (PGI2; produced by endothelium) associated with preeclampsia.^54^ This is one of the biological underpinnings for the use of low-dose aspirin for the prevention of preeclampsia. Mills et al.^55^ reported longitudinal measurements of the urinary metabolites of thromboxane and PGI2 throughout gestation. Although they did not find a significant increase in the urinary concentrations of TXA2, they did find a significant decrease in PGI2 as early as 13–16 weeks of gestation and a significant elevation in the ratio of thromboxane to PGI2 as early as 17–20 weeks of gestation in women destined to develop preeclampsia. While this PG imbalance is noted both prior to and at the time of clinical presentation (after 20 weeks), the fact that arachidonic acid metabolism was only observed in early pregnancy may explain why clinical studies note that low-dose aspirin initiation prior to 16 weeks is needed for significant prevention of preeclampsia.^56^

The tryptophan pathway was identified as highly associated with preeclampsia over gestation (Figure 7). Indoleamine-2,3-dioxygenase (IDO) is the first and rate-limiting enzyme in this pathway producing kynurenine, which then is converted into a number of bioactive metabolites. IDO is an intracellular enzyme produced by many cell types and while not secreted, impacts neighboring cells by tryptophan depletion and production of bioactive metabolites. The role of IDO in both normal and abnormal pregnancies, including preeclampsia, has been recently reviewed.^57^ IDO expression increases with pregnancy, and tryptophan depletion in the placenta inhibits T cell-mediated rejection of semi-allogeneic fetal tissues.^58^ Kynurenine is an endogenous ligand that activates the aryl hydrocarbon receptor (AhR).^59^ This activation skews the differentiation of T cells to immunosuppressive Tregs rather than proinflammatory Th17 cells after exposure to transforming growth factor β.^60^^,^^61^ Notably, kynurenic acid and xanthurenic acid, two metabolites of kynurenine, can also activate AhR signaling and may participate in immune regulation.^62^^,^^63^ Therefore, deficiency of IDO impacts Treg development. Notably, IDO knockout mice, when pregnant, develop a preeclampsia-like phenotype.^64^ The metabolic signal related to tryptophan metabolism in the model over gestation may be related to the immune signature of preeclampsia, highlighting the importance of immune alterations occurring in the later stages of preeclampsia. Caffeine metabolism was also identified as highly associated with preeclampsia over gestation. This pathway and caffeine metabolites have previously been associated with pregnancy.^25^^,^^26^

Models to predict preeclampsia early in pregnancy were previously based on maternal characteristics (demographics and medical history), followed by addition of uterine artery Doppler measurements and specific biomarkers.^65^^,^^66^^,^^67^^,^^68^^,^^69^^,^^70^ Levels of angiogenic and/or antiangiogenic proteins (PlGF, sFlt-1, and ENG), or their ratios, have been established as biomarkers with high prediction accuracy later in pregnancy.^15^^,^^41^^,^^71^ More recently, analysis of omics datasets have been successfully applied to identify various biomarkers related to preeclampsia.^10^^,^^33^^,^^72^ Most of these studies were based on measurements from one or at most two omics datasets, and often from samples taken only at one time point during pregnancy. Here we show that clinical and demographic characteristics (i.e., weight, height, race) were complementary to omics measurements and improved prediction models.

Another important problem would be to develop a more specific model to predict severe preeclampsia. Among existing models for prediction of preeclampsia based on maternal characteristics and specific biomarkers, there are fewer that predict specifically severe preeclampsia,^73^^,^^74^^,^^75^ and a prediction model from multiomics assays would be an important contribution to this literature. Given the small number of women with severe preeclampsia in our cohorts, we plan to address this topic in future studies by analyzing cohorts richer in this pregnancy outcome.

Our study is limited by a small sample size—a typical limitation when high-cost multiomics analysis is conducted—and consideration of a cohort from a single hospital. Another limitation comes from the fact that targeted assays (and untargeted assays that rely on a reference database) need to be carefully validated for the samples to which they are applied. For example, our targeted aptamer-based proteomics assay has been carefully validated in human plasma samples but cannot be readily applied to vaginal swabs without careful validation studies. Inherently to the machine-learning approach, developing a prediction model depends on the underlying sample distribution of the data used. Distribution shift, caused by differences among various cohorts, can impact the performance of a machine-learning algorithm.^76^ For this reason, we a took special care in obtaining our results by: (1) performing careful machine-learning analysis to avoid overfitting; (2) validating our model on an independent cohort; (3) demonstrating that features identified by machine learning are statistically significant when analyzed by a separate, univariate analysis; and (4) examining our prediction model in relation to a previously established model from immunological data. In this study, the mass cytometry data were not included in the multiomics prediction model because these data were not available for 14 out of 33 women. However, integrative analysis of the restricted set of common samples revealed important connections between our model and key immune features.

While encouraging, our results need to be validated on a larger, more diverse set of women. If the results prove generalizable, our findings demonstrating high predictive power from a small number of urine metabolites and proteins could lead to a simple prediction test based on a small number of urine metabolites, suitable for use in both developed and developing parts of the world.

## Experimental procedures

### Resource availability

#### Lead contact

Further information and requests for resources should be directed to and will be fulfilled by the lead contact, Ivana Marić (ivanam@stanford.edu).

#### Materials availability

This study did not generate new unique reagents.

### Study design

We performed a longitudinal, prospective study of a cohort of pregnant women receiving routine ante- and postpartum care at the Lucile Packard Children’s Hospital at Stanford University, California, as previously described.^20^^,^^77^ Women were eligible for the study if they were at least 18 years of age and were in their first trimester of pregnancy. The study was approved by the Institutional Review Board of Stanford University (#21956), and all participants signed an informed consent form.

Peripheral blood samples (for mass cytometry analysis), plasma samples (for proteomic, transcriptomic [cfRNA], metabolomic, and lipidomic analyses), urine samples (for metabolomic analysis), and vaginal swabs (for microbiome analysis) were collected from each woman at two or three time points during pregnancy. Sample collection, their analyses, and quality assessment for some of them was previously described,^9^ and are presented in the supplemental information. The validation cohort included 16 women from the same hospital, for which longitudinal samples with only metabolomic analyses were available. Metabolomic analyses were performed following the same methodology as for the discovery cohort.

### Definition of preeclampsia

Preeclampsia was defined using the American College of Obstetrics and Gynecology classification^3^ as follows: hypertension that develops after 20 weeks of gestation (systolic or diastolic blood pressure of 140 and/or 90 mmHg, respectively, measured on at least two occasions, 4 h to 1 week apart) and proteinuria (300 mg in a 24-h urine collection, a protein/creatinine ratio of at least 0.3 [each measured as mg/dL] or, if these were not readily available, a random urine specimen containing 1+ protein by dipstick). In the absence of proteinuria, preeclampsia was diagnosed if the presence of thrombocytopenia (platelet count less than 100,000/μL), impaired liver function (elevated blood levels of liver transaminases to twice the normal concentration), the new development of renal insufficiency (elevated serum creatinine greater than 1.1 mg/dL), pulmonary edema, or new-onset cerebral or visual disturbances. Early-onset and late-onset preeclampsia were distinguished based on whether diagnosis was before or after 34 weeks of gestation.

### Machine-learning analyses

Prediction models for each omics dataset were developed for each omics set using an EN model.^13^ Given N×p matrix of predictors (measurements) $X=\left(x_{1},\ldots x_{p}\right)\;$ and a vector of responses $y=\left(y_{1},\ldots ,y_{N}\right)$, regression coefficients $\beta =\left(\beta_{1},\ldots ,\;\beta_{p}\right)$ and an intercept term $\beta_{0}$ in the EN model are obtained by maximizing the likelihood, or equivalently minimizing the negative log likelihood together with $L_{1}$ and $L_{2}$ penalty:

(Equation 1) $$\left[\frac{1}{N}\sum_{i=1}^{N}L\left(\beta_{0},\beta ;y,X\right)+\lambda \left(\left(1-\alpha \right){\left\|\beta \right\|}_{2}+\alpha \left\|\beta \right\|\right)\right]\;.$$

Logistic regression was used, for which the negative log likelihood evaluates to

$$L\left(\beta_{0},\beta ;y,X\right)=\sum_{i=1}^{N}y_{i}\left(\beta_{0}+x_{i}^{T}\beta \right)-log\left(1+e^{\beta_{0}+x_{i}^{T}\beta }\right)\;.$$

For the high-dimensional setting (p≫N) considered here, EN, which performs both shrinkage and automatic selection of predictors, can provide both high accuracy and facilitate interpretability. Prior to training a model, low-variance measurements from transcriptome and microbiome were filtered out. Other omics sets did not have near-zero variance measurements.

For integration of omics datasets (Figure 4), a nested (two-level) cross-validation approach was used to train predictive models to estimate the risk of preeclampsia (Figure S15). At the first level, the EN model was used as described above (Equation 1). At the second level, predictions of EN models were integrated using stacked regression.^78^^,^^79^^,^^80^ Specifically, to use EN models in the two-level approach, for each modality *k*, k=1,…K and data $X^{k}=\left(x_{1}^{k},\ldots x_{p_{k}}^{k}\right)$, a leave-one-out EN model, denoted $c_{-i}^{k}\left(x_{i}\right)$, was repeatedly fitted and evaluated at patient i. At the second level, stacked regression with non-negative coefficients^14^ was used, so that the regression coefficients of the final model $\left(\gamma_{1},\ldots ,\;{\text{γ}}_{\text{K}}\right)$ were determined by

$$\min\sum_{i=1}^{N}{\left(y_{i}-\sum_{k=1}^{K}\gamma_{k}c_{-i}^{k}\left(x_{i}^{k}\right)\right)}^{2}s.t.\;\gamma_{i}\geq 0.$$

Note that the leave-one-out approach used in stacked regression has a purpose to form an unbiased linear combination of EN models.^79^ In contrast to the original stacking approach in which different prediction models fit on the same data are stacked, here, we use the same model (EN) but fit to different omics to obtain different estimators which are then stacked. A stacked regression model can be regarded as a special case of a two-layer neural network; its special construction provides for an easier interpretation.

We point out that the nested cross-validation is done where in each step of cross-validation, EN models for each omics set are first trained and then the stacked model is trained in the same step. After the stacked model is built, it is tested on the test patient who was left out in the outer cross-validation loop. Therefore, no leakage of information between training and test data occurred (see detailed flowchart in Figure S15). In addition, the manuscript is accompanied by data and source code to enable both independent reproduction of our results and evaluation of the machine-learning techniques used. Furthermore, tuning of two parameters of EN algorithm, λ,α shown in Equation 1 above was also performed without using the test set data: function cv.glmnet in R package glmnet was used for implementation on EN that internally performs a separate cross-validation using the training set to choose λ. The value of α was not optimized and it was set to α = 0.9.

One of our main goals was to identify a small subset of biomarkers that can predict preeclampsia with high accuracy and could thereby be used as a simple diagnostic test. For these reasons, performance of the refitted EN model for each omics set was next evaluated by treating the EN model as a model-selection procedure and performing a refitting step on the selected support (features) in the same cross-validation step.^81^ The refitted model is then tested on the test patient who was left out in the cross-validation loop (see detailed flowchart in Figure S16). It is known that $L_{1}$-penalization used in EN performs excessive shrinkage of the large coefficients of the prediction model.^82^ Refitting can resolve this problem and obtain a model with a smaller number of features.

Finally, to investigate a possible gain from integration of available clinical and demographic characteristics, a prediction model that takes omics (from a specific multiomics set) and clinical and demographics variables as an input to an EN model was fit and evaluated.

To build the model over gestation, multiple (2–3) samples available from the same patient were treated as independent inputs to the algorithm. Once prediction scores were obtained for each sample, scores for a same patient were averaged into the final risk score for that patient. Performance was estimated using a leave-one-out cross-validation procedure, such that in each cross-validation step all measurements of one patient are left out from the training set and are used for testing. In addition, urine metabolome prediction models, with and without clinical/demographics variables, were validated on a separate validation cohort. This dataset was produced independently of the initial dataset and was only used once at the end. Specifically, a prediction model was trained and its parameters determined using the discovery cohort and was then tested only once on the validation cohort. The prediction accuracy of the model in terms of the area under receiver-operating characteristics curve was evaluated. t-SNE^83^ was used for network visualization in Figure 5. For network visualization in Figure S13, a *k*-nearest-neighbor graph (with k=2) was constructed between features. The network layout was computed with the LargeVis algorithm.^84^ The analysis was performed using R software (version 3.6.1).

### Pathway enrichment analysis

Univariate analysis was performed to identify features with significant associations between each feature and the pregnancy outcome, both in early pregnancy (Wilcoxon signed-rank test) and over gestation (LME model). The Benjamini-Hochberg procedure was used to control the FDR.^85^ Metabolome pathway enrichment analysis on identified metabolites was performed using MetaboAnalyst.^86^ The hypergeometric test was used for over-representation analysis in MetaboAnalyst. Proteome pathway enrichment analysis was performed using GeneOntology^87^^,^^88^ and topology-based Gene Ontology scoring (topGo), an R software package. Circular Gene Ontology (CirGO) software for visualizing two-level hierarchically structured gene ontology terms^89^ was used to visualize proteome and transcriptome pathway enrichment.

## Data Availability

Raw and processed untargeted metabolomics data were deposited to the Metabolomics Workbench with the following study IDs: ST001889 for plasma and ST001890 for urine. The Project DOI for these studies is https://doi.org/10.21228/M8WD84. Our microbiome reads have been submitted to SRA. The BioProject accession is PRJNA752652. https://dataview.ncbi.nlm.nih.gov/object/PRJNA752652?reviewer=aofjjbr2j556u6vckeolc1i2t2. Transcriptome data are available at: https://drive.google.com/file/d/12JXm30he5psipz6iCiIUtZxWsy-IwG08/view?usp=sharing. The data that support the findings of this study have also been deposited on GitHub in the form of csv files at https://github.com/ivanam5/Multiomics_Preeclampsia. All data except for the clinical variables have been made available. Clinical variables cannot be shared due to the HIPAA constraints. Code to reproduce main analyses in the manuscript is available on GitHub at https://github.com/ivanam5/Multiomics_Preeclampsia. R software is needed to run the code.

## Supplemental information

Document S1. Supplemental experimental procedures, Figures S1–S22, and Tables S1–S5

Document S2. Article plus supplemental information
