# Supplementary material for: Early prediction and longitudinal modeling of preeclampsia from multiomics
Source: Patterns (N Y). 2022 Dec 9;3(12):100655. doi: 10.1016/j.patter.2022.100655 (PMC9768681; doi:10.1016/j.patter.2022.100655)
Supplement: Document S1. Supplemental experimental procedures, Figures S1–S22, and Tables S1–S5 [file mmc1.pdf]

Patterns, Volume *3*

Supplemental information

Early prediction and longitudinal modeling of preeclampsia from multiomics

Ivana Marić, Kévin Contrepois, Mira N. Moufarrej, Ina A. Stelzer, Dorien Feyaerts, Xiaoyuan Han, Andy Tang, Natalie Stanley, Ronald J. Wong, Gavin M. Traber, Mathew Ellenberger, Alan L. Chang, Ramin Fallahzadeh, Huda Nassar, Martin Becker, Maria Xenochristou, Camilo Espinosa, Davide De Francesco, Mohammad S. Ghaemi, Elizabeth K. Costello, Anthony Culos, Xuefeng B. Ling, Karl G. Sylvester, Gary L. Darmstadt, Virginia D. Winn, Gary M. Shaw, David A. Relman, Stephen R. Quake, Martin S. Angst, Michael P. Snyder, David K. Stevenson, Brice Gaudilliere, and Nima Aghaeepour

Supplemental Experimental Procedures

Content:

Tables S1 to S5

Figures S1 to S22

Supplemental Experimental Procedures

Table S1. Patient and pregnancy characteristics

	**Discovery Cohort 1 (N=33)**		**Validation Cohort (N=16)**	
**Demographics**	*Controls (N=16)*	*Preeclampsia (N=17)*	*Controls (N=4)*	*Preeclampsia (N=12)*
**Maternal age at enrollment**				
(years, mean ± SD)	32.1 ± 4.9	31.1 ± 6.3	30.7 ± 4.8	32.3 ± 4.5
**Gravida** (N, % nulliparous)	7 (43.7)	6 (35.3)	2 (50)	5 (41.7)
**Ethnicity** (N, %)				
Hispanic	0 (0)	8 (47)	0 (0)	2 (16.7)
Non-Hispanic	16 (100)	9 (53)	4 (100)	9 (75)
Unknown	0 (0)	0 (0)	0 (0)	1 (8.3)
**Race (N, %)**				
White	16 (100)	9 (52.9)	4 (100)	5 (41.7)
African-American	0 (0)	1 (6.0)	0 (0)	1 (8.3)
Asian	0 (0)	4 (23.5)	0 (0)	4 (33.3)
Unknown	0 (0)	3 (17.6)	0 (0)	1 (8.3)
Other	0 (0)	0 (0)	0 (0)	1 (8.3)
**Preexisting hypertension**	0 (0)	4 (23.5)	0 (0)	4 (33.3)
**Height**				
(cm, mean ± SD)	166.9 ± 7.4	158.8 ± 6.2	163 ± 3.5	163.8 ± 7.7
**Weight**				
(kg, mean ± SD)	61.9 ± 9.1	74.0 ± 20.3	62.6 ± 8.1	79.6 ± 25.9
**BMI** (mean ± SD)	22.8 ± 3.3	29.4 ± 7.9	23.5 ± 2.5	29.4 ± 7.7
**Multiple gestation** (N, %)	0 (0)	2	0 (0)	0 (0)
**Baby gender (male N, %)**	8 (50)	11 (64.7)	4 (100)	6 (50)

Table S2. Preeclampsia patient characteristics.		
	**Cohort 1 (n=17)**	**Validation Cohort (n=12)**
**Gestational age at the onset of preeclampsia** (mean ± SD)	35.8 ± 3.8	36.6 ± 3.7
**Early Onset** (N, %)	5 (29.4)	1 (8.3)
**Severe preeclampsia** (N, %)	10 (58.8)	7 (58.3)

Table S3. List of annotated urine metabolites selected in EN models.

Compound ID	Mode	Molecular Ion	Metabolite	Formula	KEGG	HMDB	MSI annotation level
368.2789_8.5	pRPLC	[M+H]+	C14:2 AC (Tetradecadiencarnitine)	C21H37NO4		HMDB 13331	2
249.0074_2.8	nRPLC	[M-H]-	Dihydroxyphenylglycol O-sulfate	C8H10O7S		HMDB 01474	3
153.0193_1.5	nRPLC	[M-H]-	Dihydroxybenzoic acid	C7H6O4		HMDB 13676	2
298.2009_5.1	pRPLC	[M+H]+	C9:2 AC (Nonadienoylcarnitine)	C16H27NO4			2
632.2045_0.8	nRPLC	[M-H]-	Sialyllactose	C23H39NO19		HMDB 00825	2
263.0231_4.8	nRPLC	[M-H]-	Methoxy hydroxyphenylethyleneglycol sulfate	C9H12O7S		HMDB 00559	3
359.0984_3.9	nRPLC	[M-H]-	Methoxy hydroxyphenylglycol glucuronide	C15H20O10	C03033	HMDB 00496	3
136.0614_0.9	pRPLC	[M+H]+	Adenine	C5H5N5	C00147	HMDB 00034	1
385.2366_8.1	pRPLC	[M+H-H2O]+	Dehydrocholic acid	C24H34O5			2
189.1598_17.5	pHILIC	[M+H]+	N6,N6,N6-Trimethyl-L-lysine	C9H20N2O2	C03793	HMDB 01325	1
131.0713_2.3	nRPLC	[M-H]-	C6:0,OH FA (Hydroxyhexanoic acid)	C6H12O3		HMDB 00409	2
425.0804_12.3	nHILIC	[M-H]-	Cysteineglutathione disulfide	C13H22N4O8S2		HMDB 00656	3

232.1178_3.5	pRPLC	[M+H]+	Isovalerylglutamic acid	C10H17NO5		HMDB 00726	3
352.1246_3.4	pRPLC	[M+H]+	N-Acetyl-O-acetylneuraminic acid	C13H21NO10		HMDB 60492	3
289.2159_8.8	pRPLC	[M-H]-	Dehydroepiandrosterone	C19H28O2	C01227	HMDB 00077	3
169.1235_7.9	nRPLC	[M-H]-	C10:1 FA (Decenoic acid)	C10H18O2		HMDB 41012	2
189.0767_3.1	nRPLC	[M-H]-	C8:0, OH DC FA (Hydroxysuberic acid)	C8H14O5		HMDB 00325	3
176.1029_0.5	pRPLC	[M+H]+	Citrulline	C6H13N3O3	C00327	HMDB 00904	1
299.0631_2	nRPLC	[M-H]-	Uric acid ribonucleoside	C10H12N4O7	C05513	HMDB 29920	3
189.1234_8.2	pHILIC	[M+H]+	N-epsilon-acetyl-L-lysine	C8H16N2O3	C02727	HMDB 00206	1
202.1437_6.8	pRPLC	[M+H]+	N-Acetylaminooctanoic acid	C10H19NO3		HMDB 59745	3
302.2323_6.2	pRPLC	[M+H]+	C9:0 AC (Nonanoylcarnitine)	C16H31NO4		HMDB 13288	2
157.0602_8.2	pHILIC	[M+H]+	Imidazolelactic acid	C6H8N2O3	C05132	HMDB 02320	3
139.0497_0.7	pRPLC	[M+H]+	Nicotinamide N-oxide	C6H6N2O2		HMDB 02730	1
263.023_1.7	nRPLC	[M-H]-	Methoxy hydroxyphenylethyleneglycol sulfate	C9H12O7S		HMDB 00559	3
209.0665_8.5	nHILIC	[M-H]-	1,5-anhydroglucitol (1,5-AG)	C7H14O7	C07326	HMDB 02712	3
314.2324_5.5	pHILIC	[M+H]+	C10:1 AC (Decenoylcarnitine)	C17H31NO4		HMDB 13205	2
230.1034_3.5	nRPLC	[M-H]-	Isovalerylglutamic acid	C10H17NO5		HMDB 00726	3
284.1854_3.9	pRPLC	[M+H]+	C8:2 AC (Octadienoylcarnitine)	C15H25NO4			2
281.1494_2	pHILIC	[M+H]+	Tyr-Val	C14H20N2O4		HMDB 29118	2
342.2634_8	pRPLC	[M+H]+	C12:1 AC (Dodecenoylcarnitine)	C19H35NO4		HMDB 13326	1
314.2323_6.7	pRPLC	[M+H]+	C10:1 AC (Decenoylcarnitine)	C17H31NO4		HMDB 13205	2
153.0547_5	pRPLC	[M+H]+	2-Hydroxyphenylacetic acid	C8H8O3	C05852	HMDB 00669	2
448.3065_4.7	nHILIC	[M-H]-	Glycoursodeoxycholic acid	C26H43NO5		HMDB 00708	3
166.0862_10.4	pHILIC	[M+H]+	Pyridinebutanoic acid	C9H11NO2		HMDB 01007	3

330.227_5.7	pRPLC	[M+H]+	C10:1, OH AC (Hydroxydecenoylcarnitine)	C17H31NO5			2
263.1289_6.1	nRPLC	[M-H]-	gamma-CEHC	C15H20O4		HMDB 01931	2
565.3016_9.4	nRPLC	[M-H-H2O]-	Cholic acid glucuronide	C30H46O10		HMDB 02577	3
286.1396_11.1	pHILIC	[M+H]+	Glycylprolylhydroxyproline	C12H19N3O5		HMDB 02171	3
467.2655_9.8	nRPLC	[M-H]-	5alpha-Androstan-3alpha,17beta-diol 17-glucuronide	C25H40O8			3
258.1698_3.1	pRPLC	[M+H]+	C6:1 AC (Hexenoylcarnitine)	C13H23NO4		HMDB 13161	2
302.2325_5.5	pHILIC	[M+H]+	C9:0 AC (Nonanoylcarnitine)	C16H31NO4		HMDB 13288	2
229.1545_0.5	pRPLC	[M+H]+	N,N,N-trimethyl-alanylproline betaine (TMAP)	C11H20N2O3		HMDB 0240365	2
230.1031_8.4	nHILIC	[M-H]-	Isovalerylglutamic acid	C10H17NO5		HMDB 00726	3
455.2473_12	nRPLC	[M-H]-	Sulfolithocholic acid	C24H40O6S		HMDB 00907	2
360.2744_5.5	pHILIC	[M+H]+	C12:0,OH AC (Hydroxydodecanoylcarnitine)	C19H37NO5		HMDB 13164	2
448.307_9.3	nRPLC	[M-H]-	Glycoursodeoxycholic acid	C26H43NO5		HMDB 00708	3
514.284_4.8	nHILIC	[M-H]-	Taurocholic acid	C26H45NO7S	C05122	HMDB 00036	3
176.103_9.1	pHILIC	[M+H]+	Citrulline	C6H13N3O3	C00327	HMDB 00904	1
129.0658_8.5	pHILIC	[M+H]+	Dihydrothymine	C5H8N2O2	C00906	HMDB 00079	1
100.0757_1.2	pRPLC	[M+H]+	2-Piperidinone	C5H9NO		HMDB 11749	2
375.2888_11	pRPLC	[M+H]+	Hydroxycholenoic acid	C24H38O3		HMDB 00308	3
144.0301_5.9	nHILIC	[M-H]-	Keto-glutaramic acid	C5H7NO4	C00940	HMDB 01552	3

**Table S4. Related references to proteins identified by our prediction model.**		
**Protein**	**Function**	**Mechanism**
LEP[1-5]	Immune regulatory hormone	Possibly contributes to the aberrant immune signature
VEGFA[6,7]	Angiogenic factor	Lack of VEGF causes endothelial cell dysfunction
SELE[8]	Adhesion molecule	
SELL[9-11]	Marker for inflammation	Several mechanisms possible (conflicting results reported)
ROR1[12]	Tyrosine kinase receptor	Downregulation inhibits human trophoblast cell proliferation, migration, and invasion
CXCL10[13]	Pro-inflammatory and anti-angiogenic chemokine	May reflect enhanced systemic inflammatory response
SPARCL1[14]	Impedes trophoblast migration and invasion	Transcriptional profile revealed downregulation in preeclampsia
IL-24[15]	Cytokine	MiRNA-203a-3p inhibits inflammatory response in preeclampsia by regulating IL24
HIPK3[16]	Impacts biological behavior of trophoblast cells	Affects migration, invasion and proliferation of trophoblast cells

Figures:

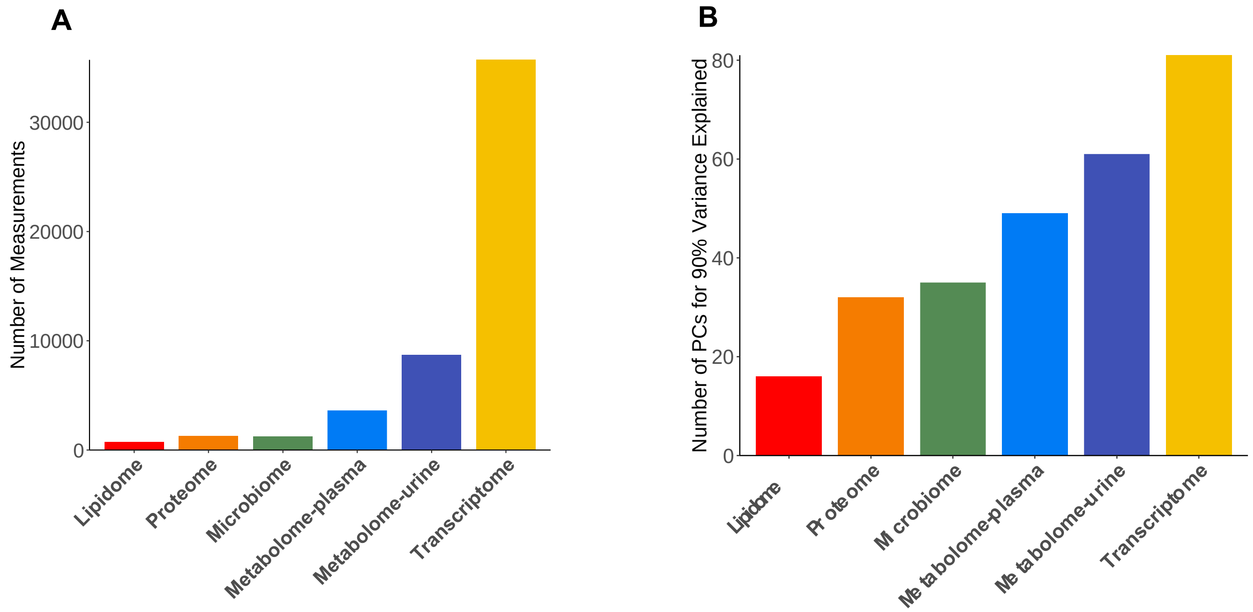

Figure S1. Features of six omics datasets. A. Number of measurements in each dataset; **B.** Number of principal components to account for 90% of the variance. Datasets containing more strongly correlated features yield fewer principal components.

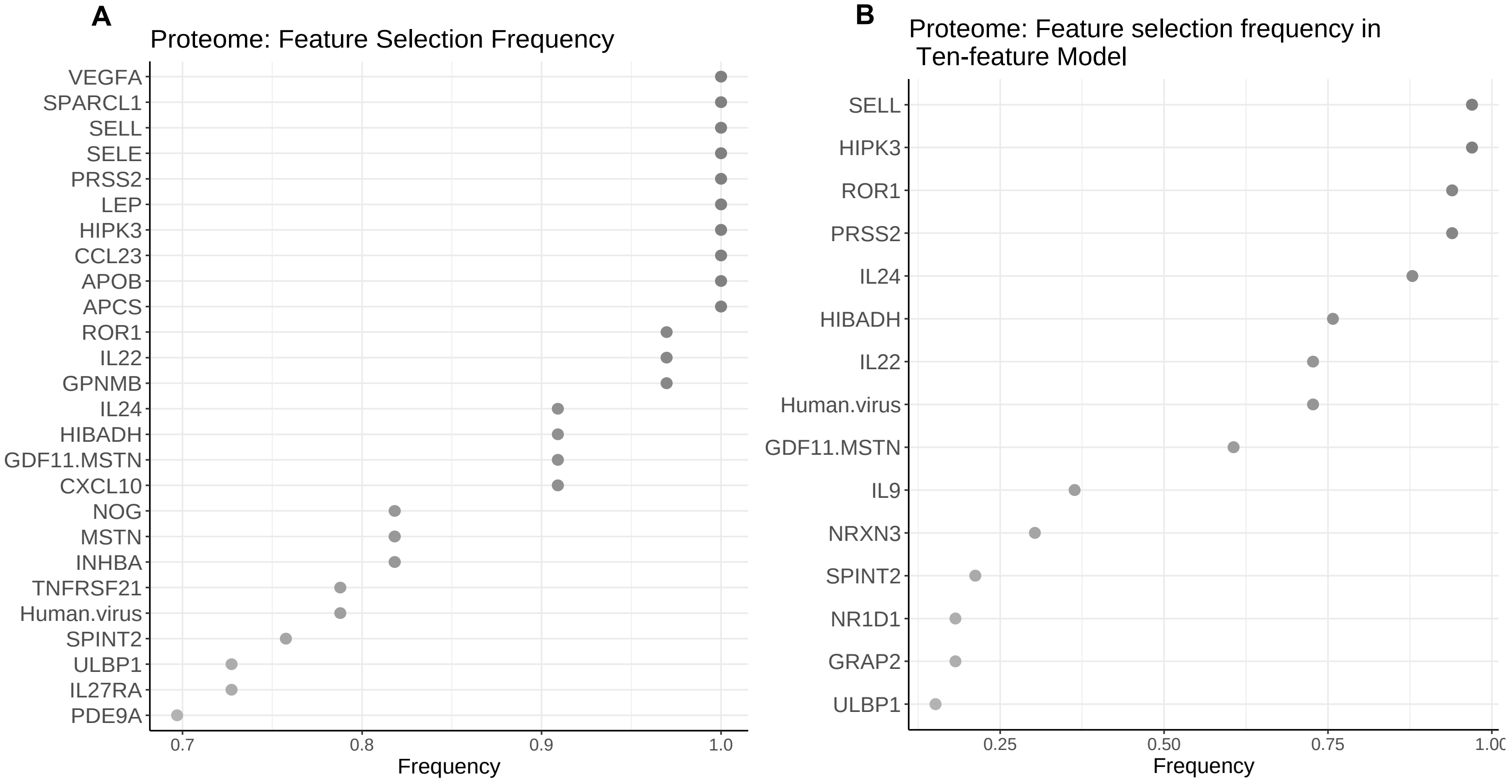

Figure S2. Proteomics feature selection frequency in prediction model over gestation. Each model was obtained using all available samples over gestation. **A. Elastic net model. B. Elastic net model with ten features.** Y-axis shows proteins chosen with the highest frequency across all EN prediction models, where one EN prediction model is built in each cross-validation step. X-axis shows the frequency with which each protein is chosen across all models.

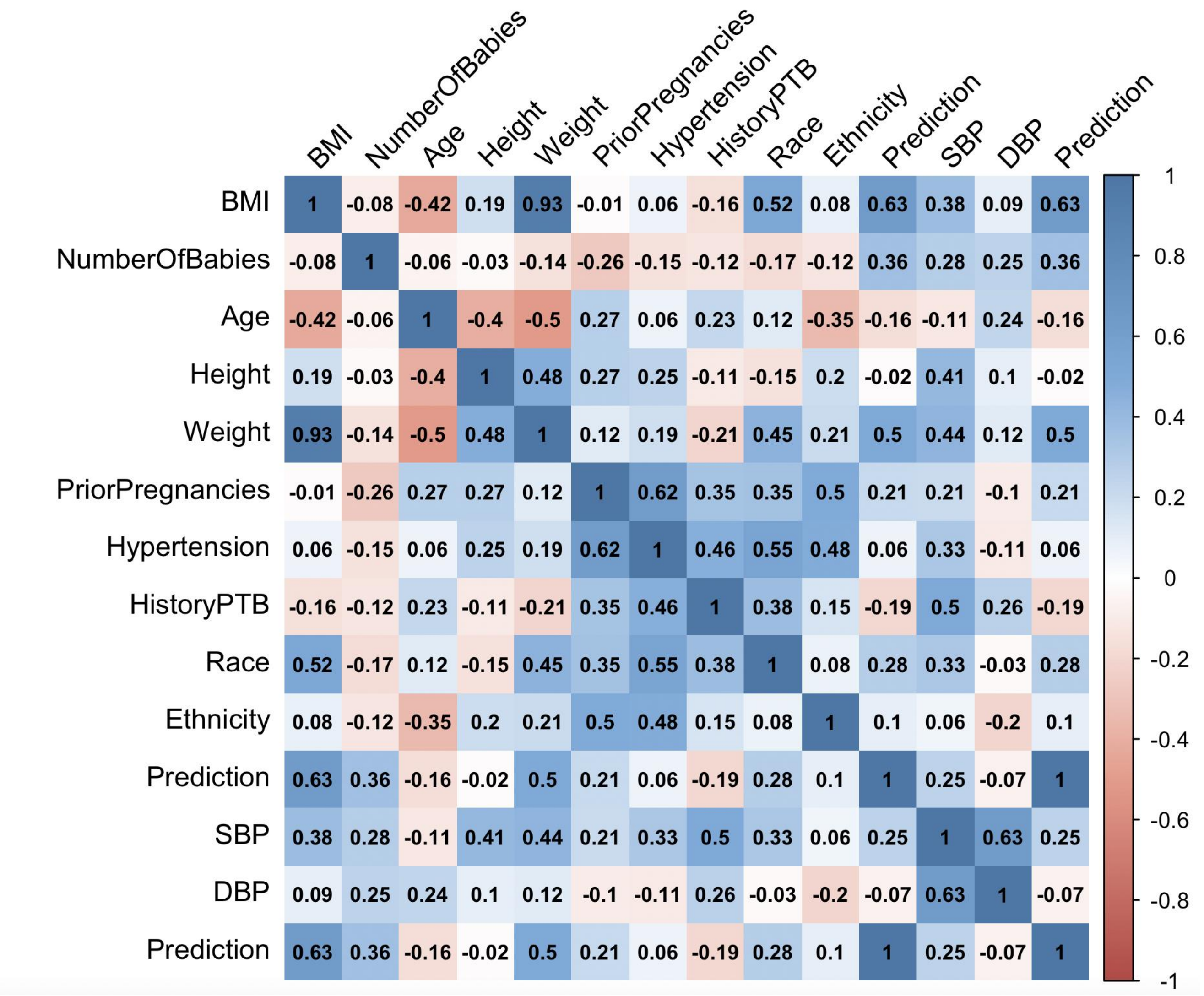

Figure S3. Spearman correlation between predictions obtained from EN model for urine metabolome using available samples over gestation and available clinical variables. The highest correlation, and the only one that was statistically significant was with BMI (p< 0.0086).

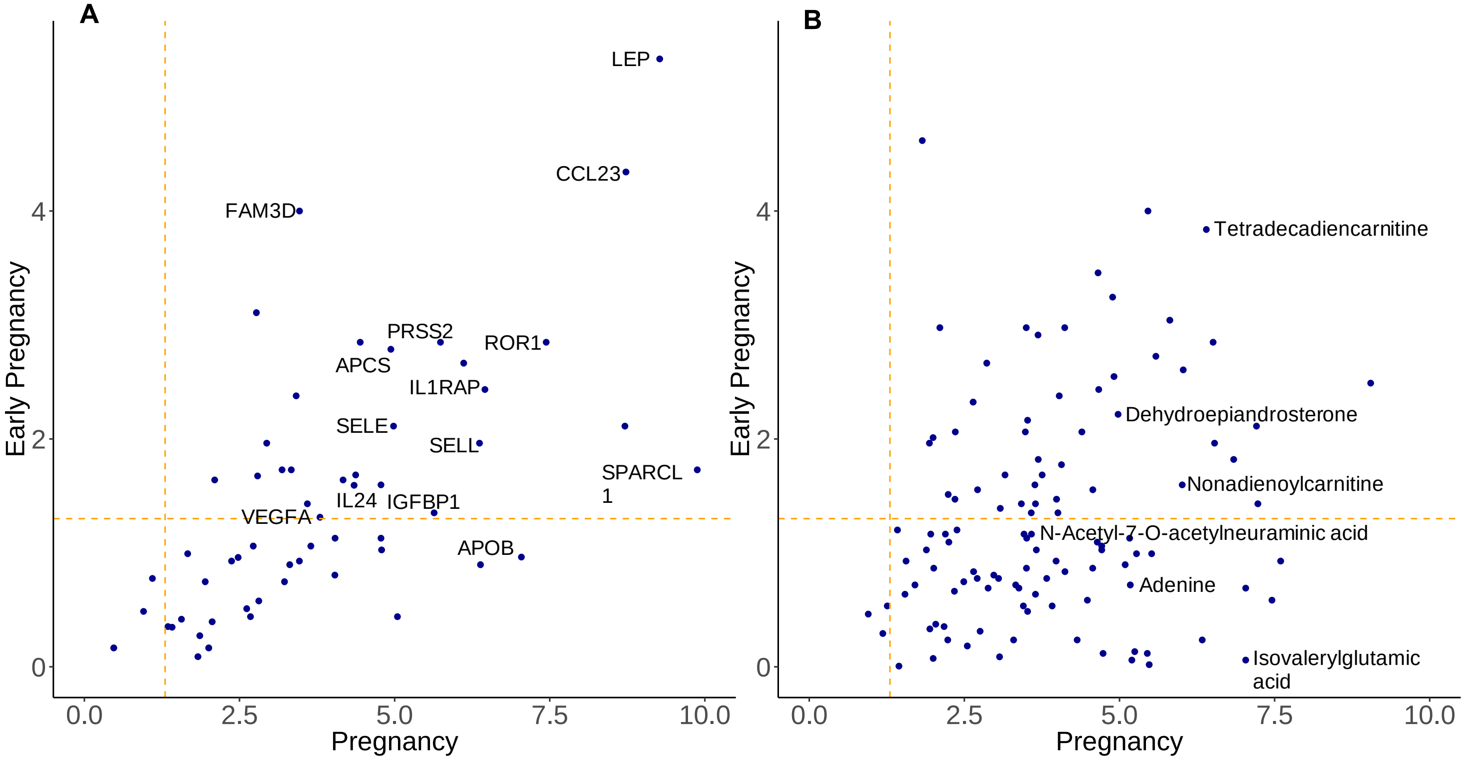

Figure S4. Biomarker comparison: entire pregnancy vs. early pregnancy. X-axis and Y-axis show -log(p-value) of each biomarker in early pregnancy and over gestation. **A.** Most predictive proteins. **B.** Most predictive urine metabolites. All values higher than -log(0.05) indicated by the orange line are significant. We observe that most of the biomarkers regardless of the prediction model are statistically significant over gestation. Less biomarkers are statistically significant in early pregnancy which is expected due to a smaller number of samples.

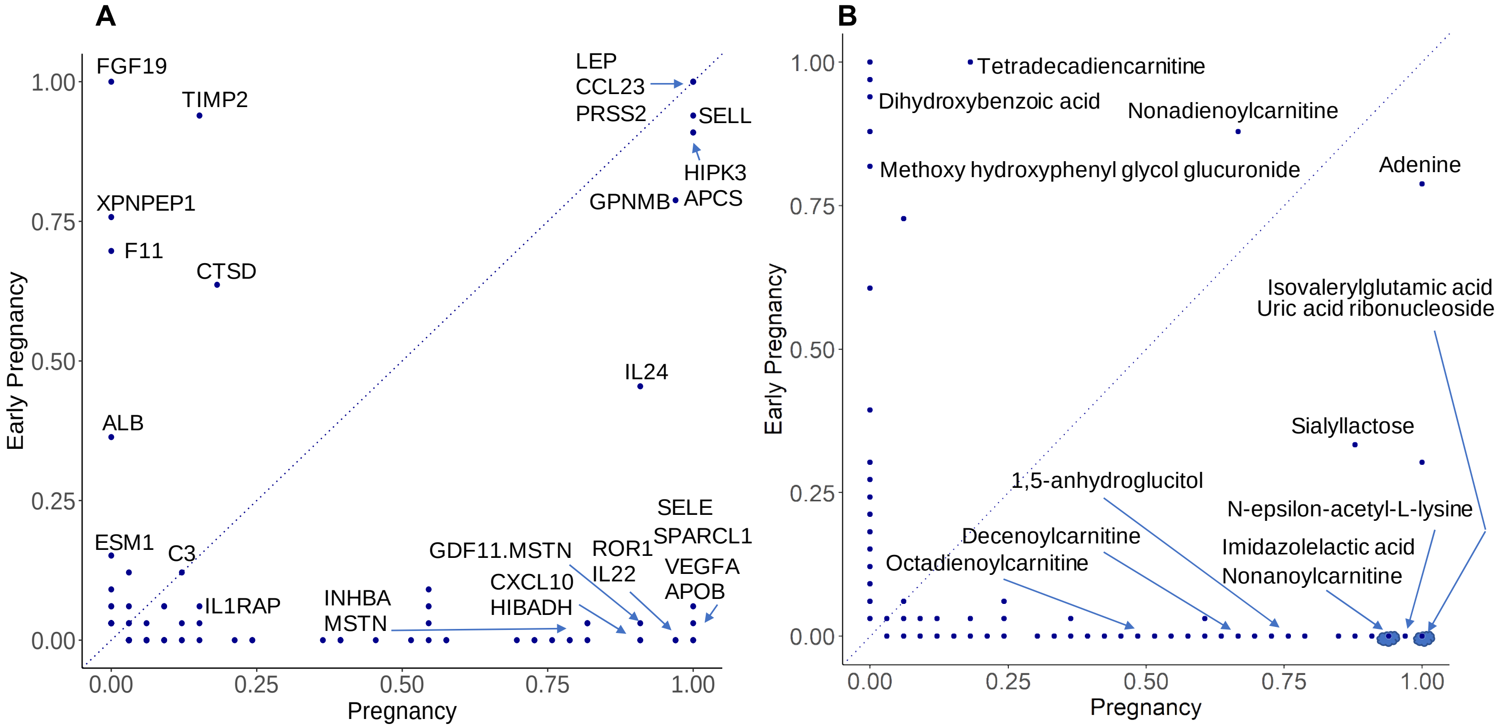

Figure S5. Biomarker comparison: entire pregnancy vs. early pregnancy. X-axis and Y-axis show the respective frequency of each biomarker in early pregnancy and over gestation. **A.** Most predictive proteins. **B.** Most predictive urine metabolites. Blue circles around dots imply the same position for more than one protein/urine metabolite.

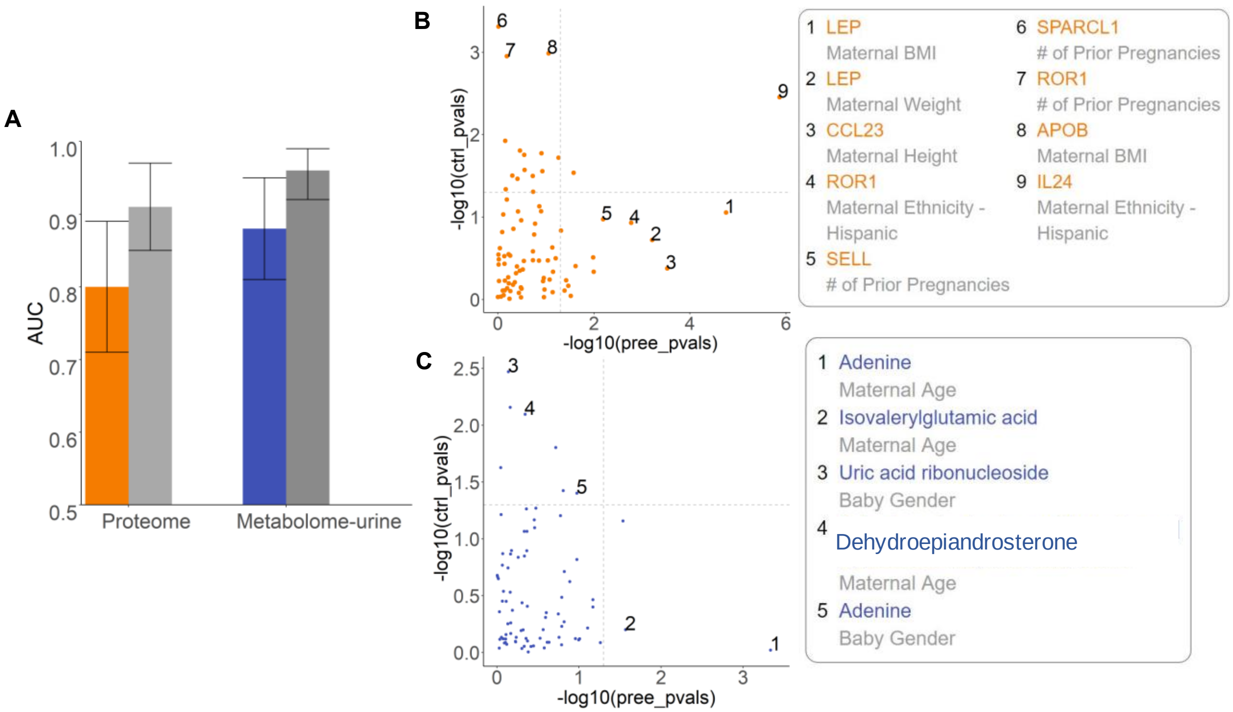

Figure S6. Relationship between urine metabolome and proteome with clinical features over gestation. A. Prediction accuracy of urine metabolome and plasma proteome. Dark blue (for urine metabolome) and orange (for proteome) bars show performance without clinical data (proteome: AUC = 0.83, 95% CI: [0.73, 0.92); urine metabolome: AUC = 0.88, 95% CI [0.81, 0.95]). Grey bars show performance with clinical data (proteome AUC=0.91, 95% CI: [0.85, 0.97]; urine metabolome AUC=0.96, 95% CI: [0.92, 0.99]). **B**. Comparison of P-value of correlations of the top proteome and clinical features. Value of $-\log_{10} P$ for preeclamptic patients and controls is shown on x-axis and y-axis, respectively. Each node is a pair of a proteome and a clinical feature. **C.** Comparison of P-value of correlations of the top urine metabolites and EHR features. Each node is a pair of a proteome/urine metabolome and a clinical feature.

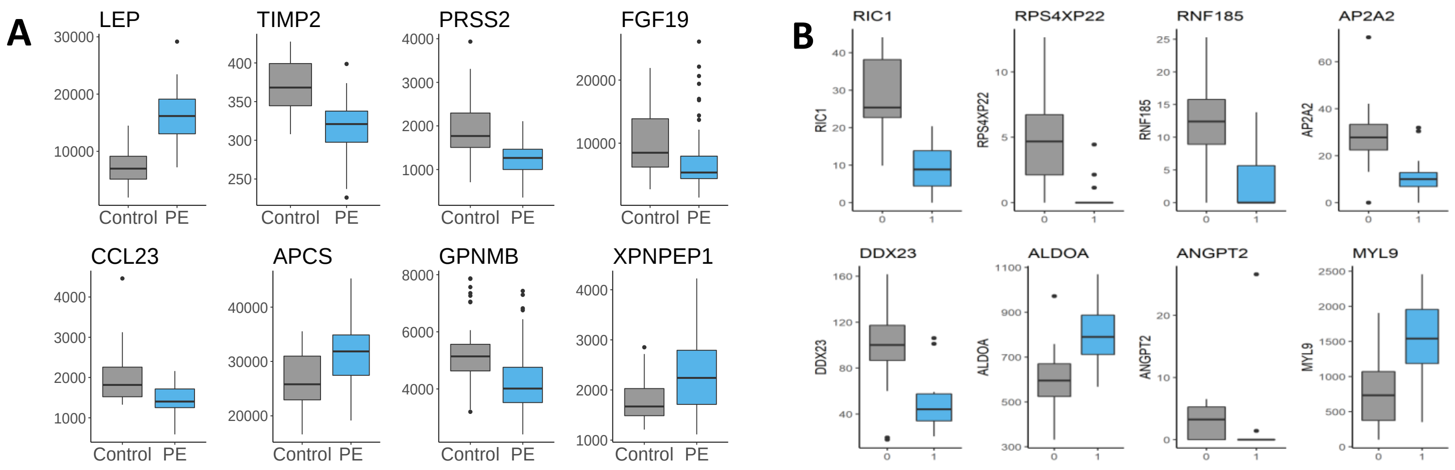

Figure S7. Top ranking proteins and genes identified by prediction models in early pregnancy. **A**. Top-ranking proteins. **B.** Top-ranking genes. Y-axis shows the value in early pregnancy stratified by normal (grey) versus preeclamptic pregnancy (light-blue).

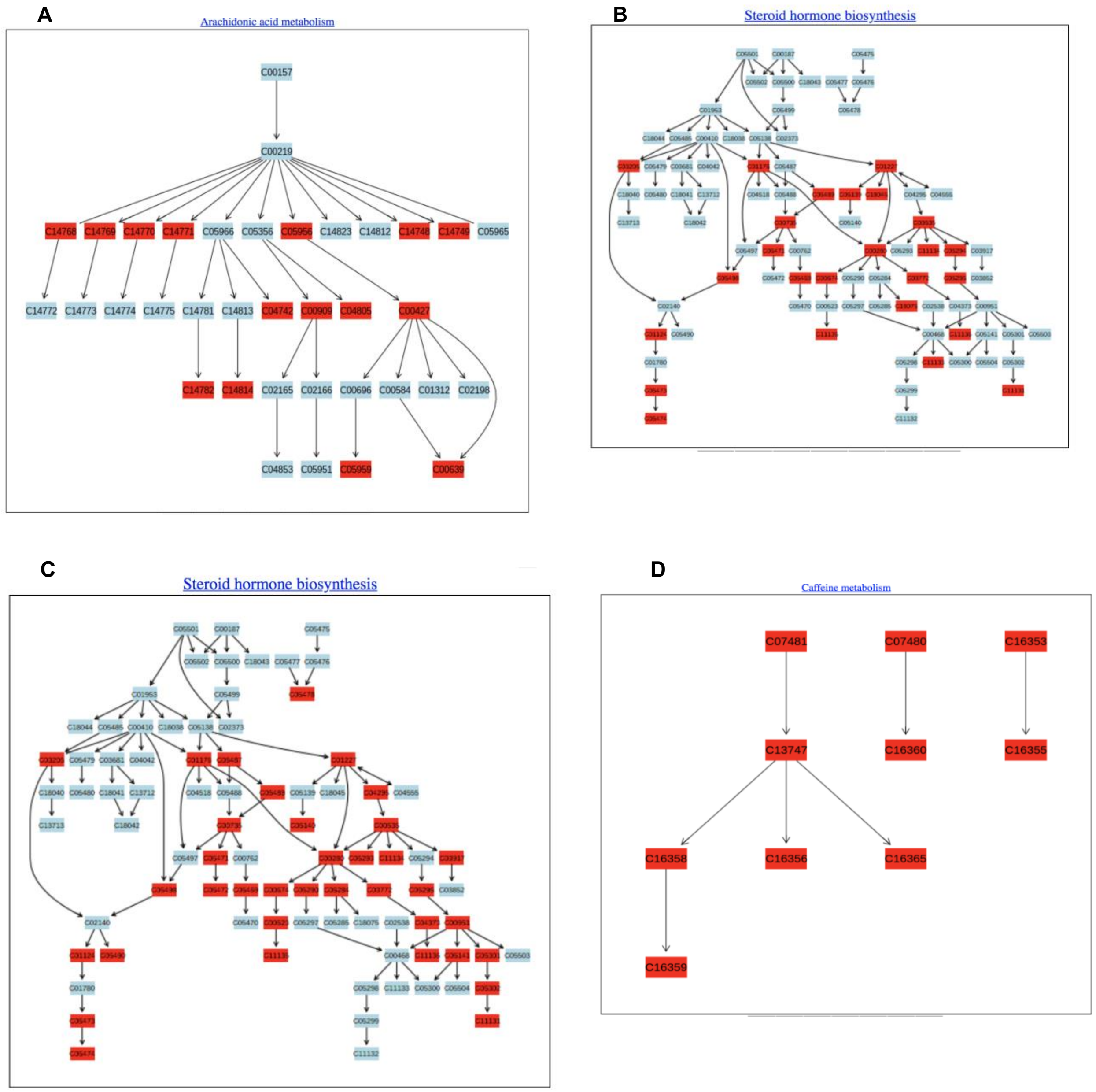

Figure S8. Identified pathways enriched in urine metabolomic data obtained in early pregnancy and over gestation. A. Metabolites in the arachidonic acid pathway in early pregnancy. Metabolites present in the data with high level of significance are shown in red. Metabolites not present in the data are shown in light blue. **B.** Metabolites in the steroid hormone biosynthesis pathway in early pregnancy. **C.** Metabolites in the steroid hormone biosynthesis pathway over gestation. **D**. Metabolites in the caffeine metabolism pathway over gestation.

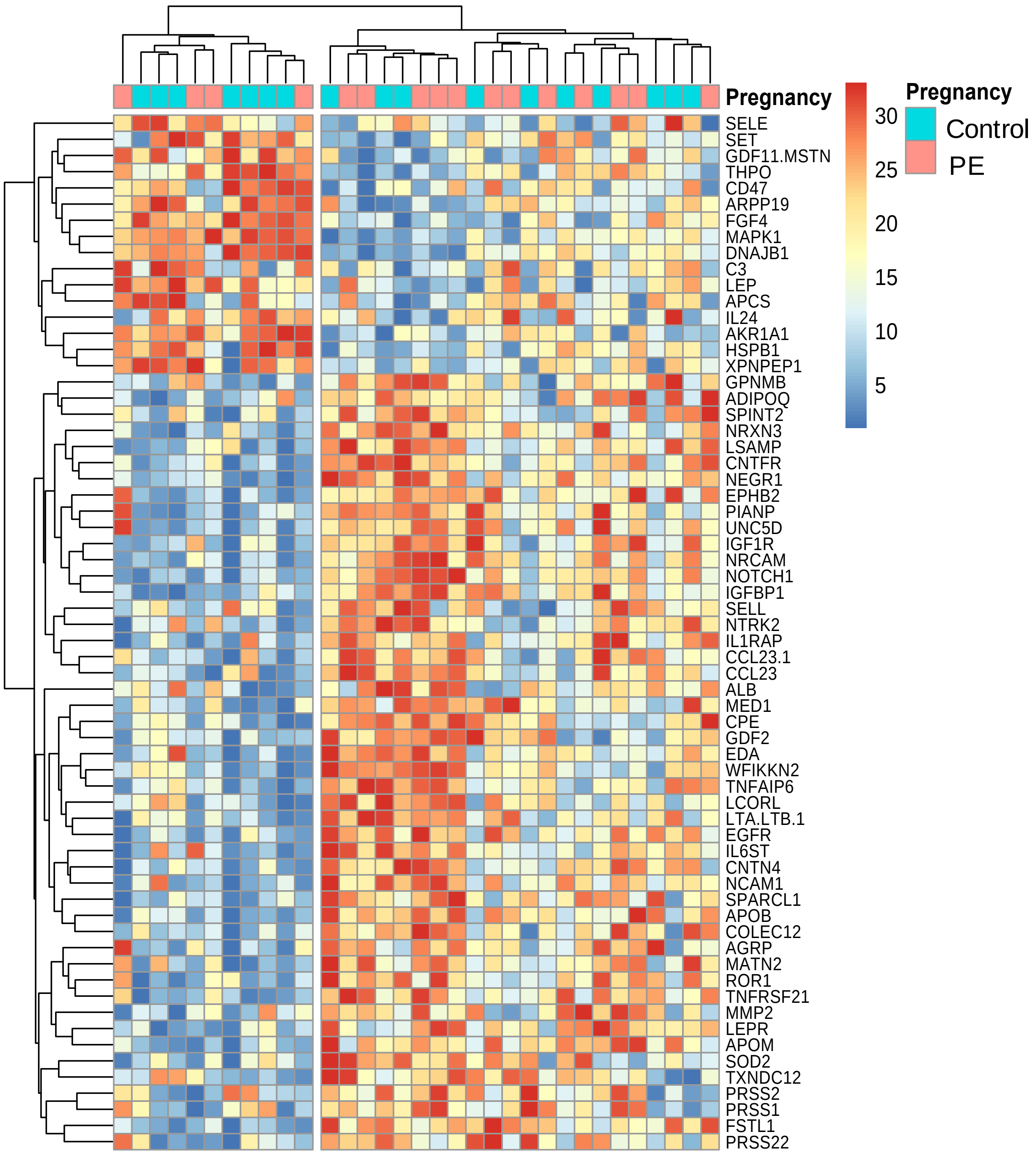

Figure S9. Univariate analysis of proteomic data collected over gestation. Heatmap of the ranked average value of the protein over three trimesters. Changes over gestation of 437 proteins were significantly associated with preeclampsia outcome (Benjamini-Hochberg, FDR < 0.05); 64 proteins with the smallest p-value ($p < 5 \cdot 10^{-5}$, Linear Mixed-Effects Model) are shown.

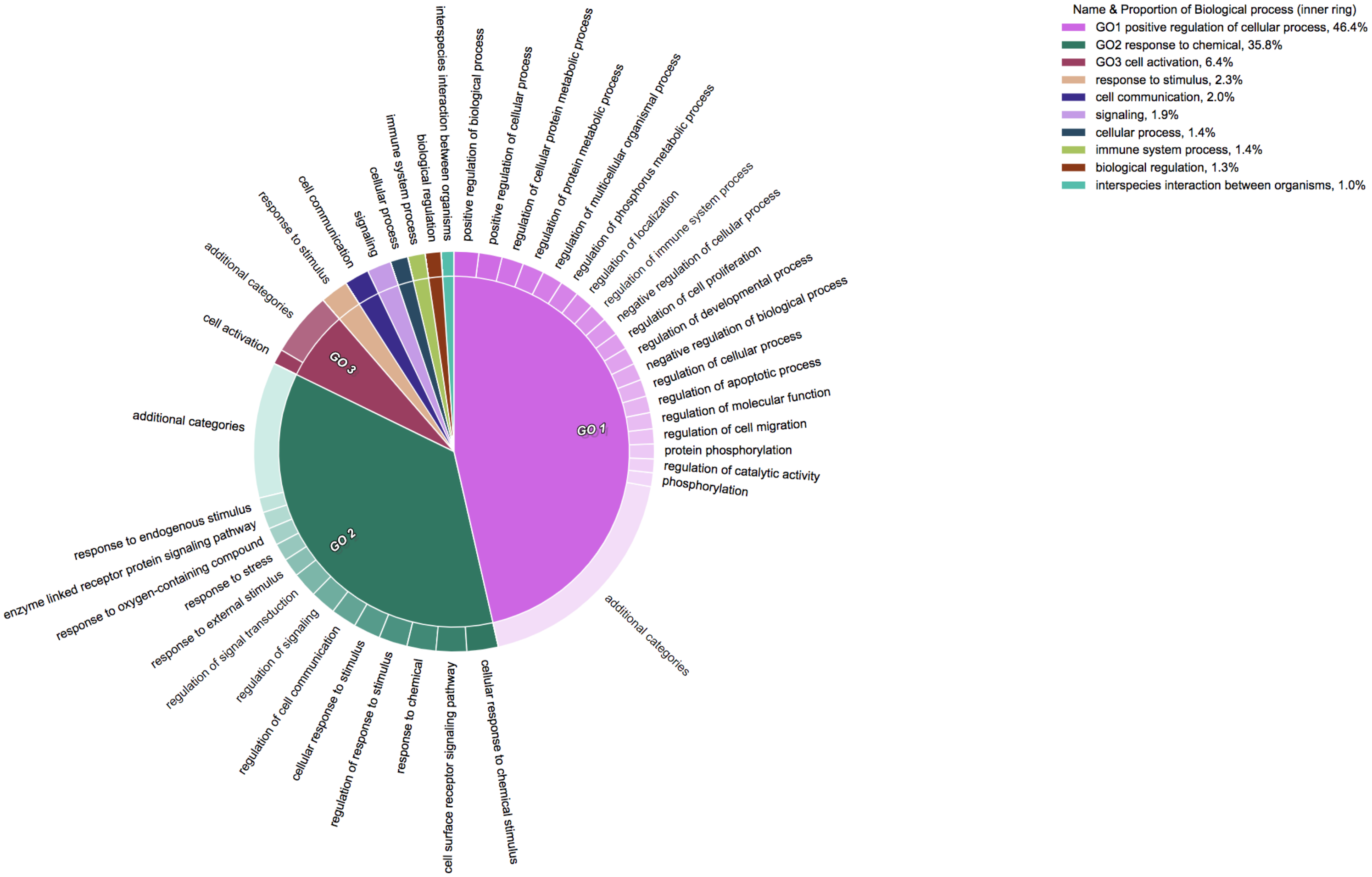

Figure S10. Enriched protein pathways grouped into ten biological processes. Enriched pathways were obtained using all available samples over gestation. The most prevalent biological process was positive regulation of cellular process was (46.4%).

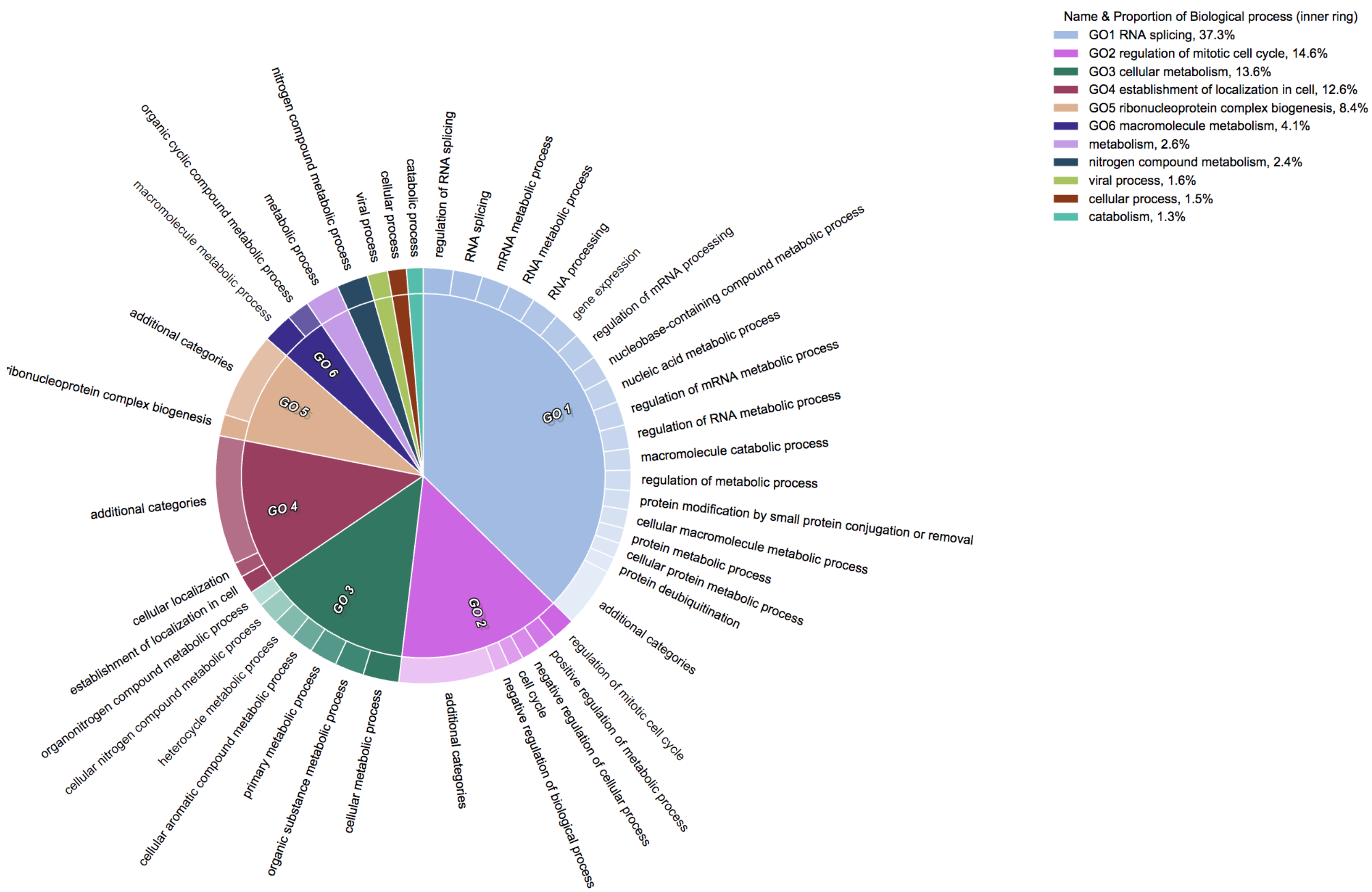

Figure S11. Enriched cfrna pathways in two-level hierarchical structure grouped into eleven biological processes. Enriched pathways were obtained using all available samples over gestation. The most prevalent biological process was RNA splicing (37.3%).

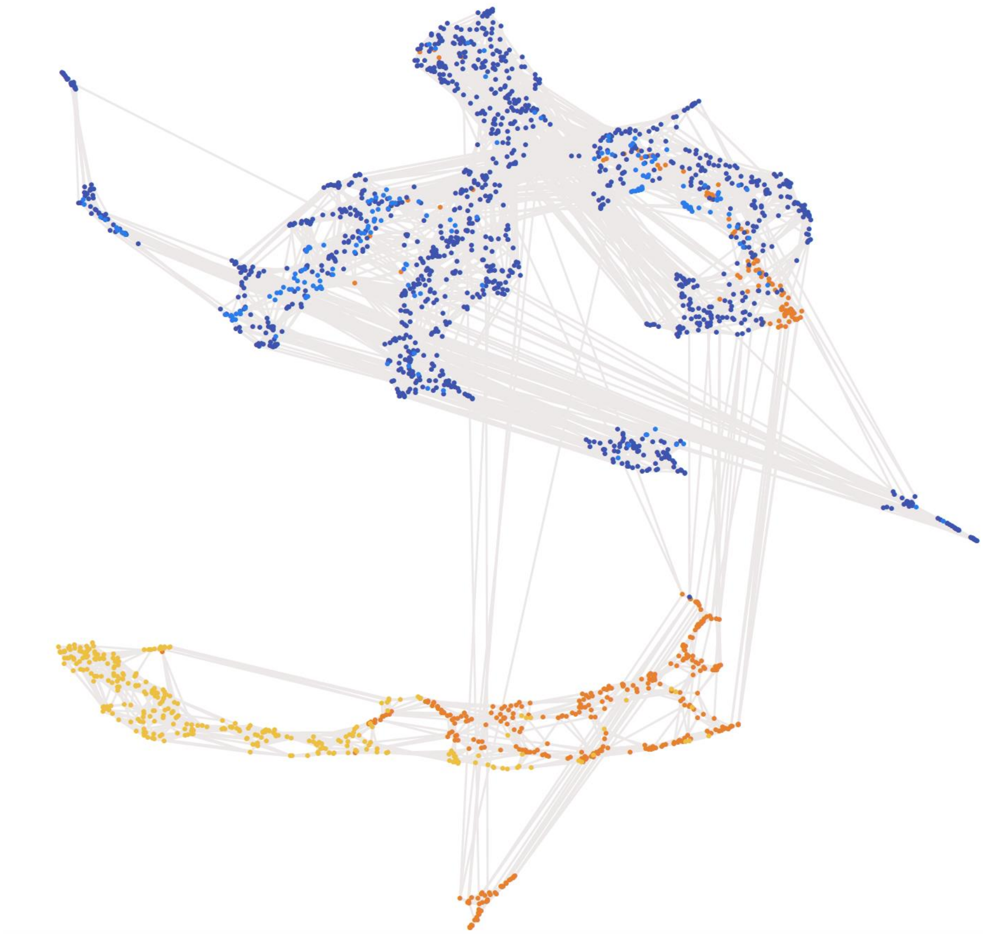

Figure S12. Network of features from different omics sets over gestation. Features with significant association with preeclampsia are shown (FRD<0.05, Linear Mixed-Effects Model with Bonferroni-Hochberg correction). Proteome, urine metabolome, plasma metabolome and transcriptome are shown respectively in orange, dark blue, light blue and yellow. 17 distinct communities were identified.

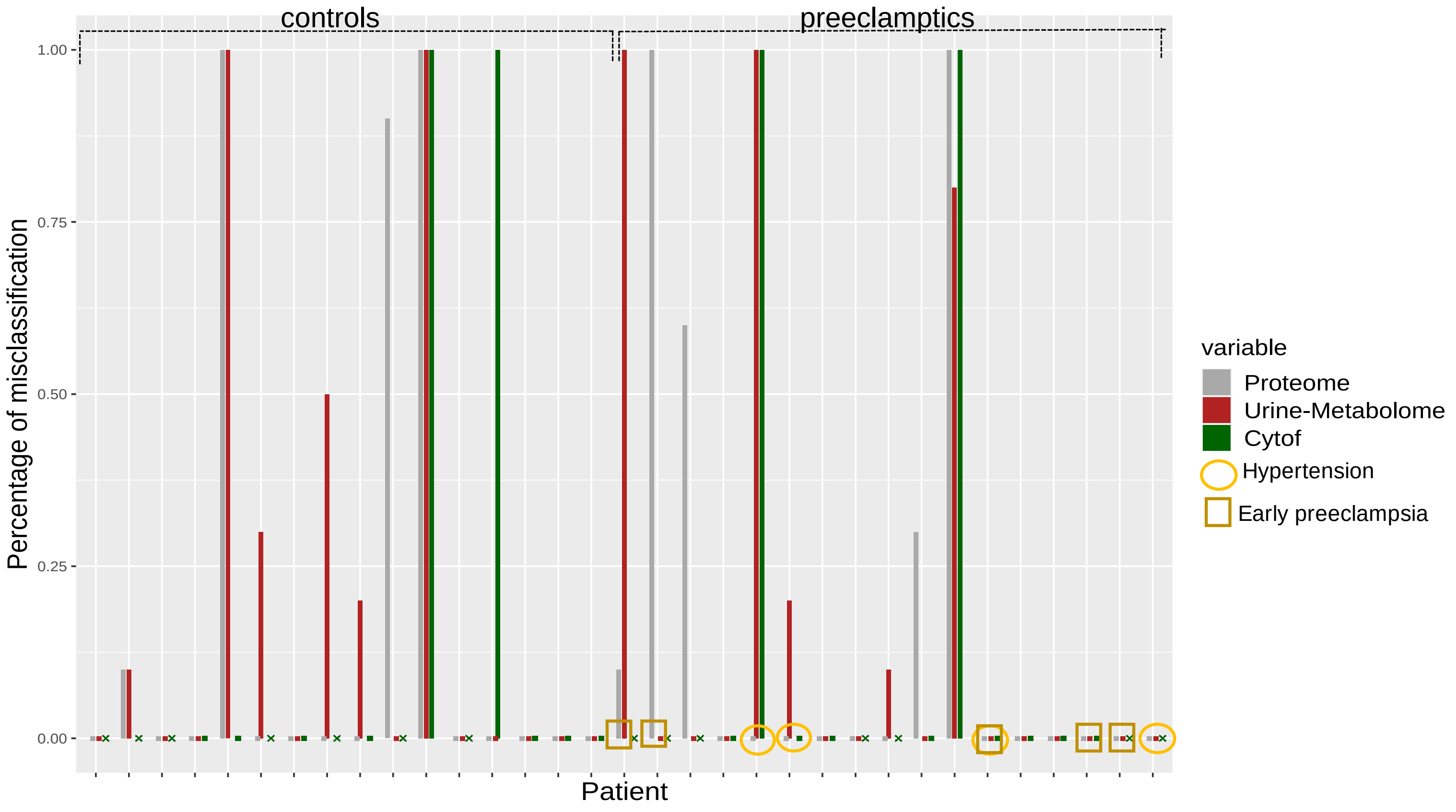

Figure S13. Misclassification rate per each patient in the cohort. Misclassification rate is shown for three prediction algorithms: proteome (gray), urine metabolome (red) and immunome (green).

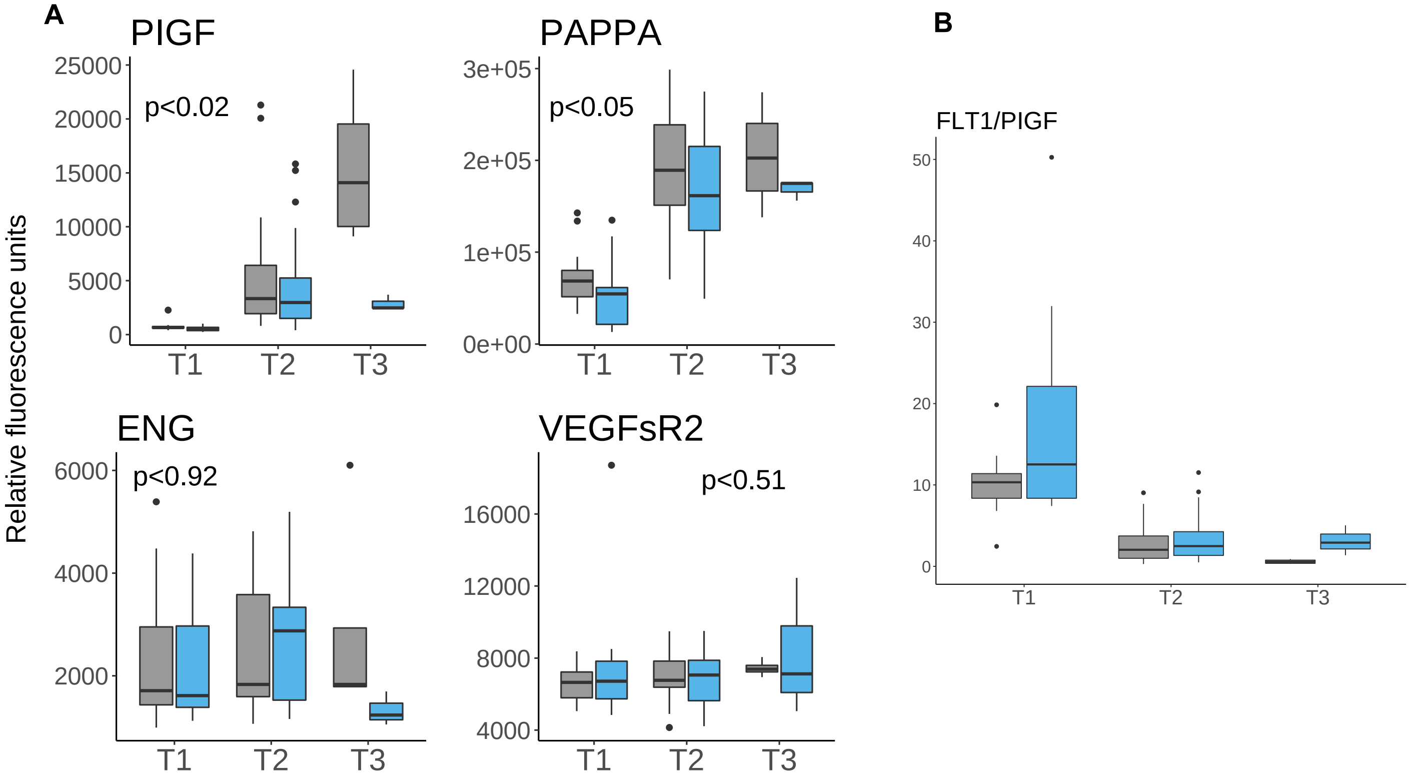

Figure S14. A. Values of known preeclampsia biomarkers over gestation. Values for Placenta growth factor (PIGF), pregnancy-associated plasma protein-A (PAPP-A), endoglin (ENG), vascular endothelial growth factor receptor 2 (VEGFsR2) proteins are shown. For VEGFsR2, the corresponding gene is sFLT-1. Y-axis shows a value stratified by normal pregnancy (grey) and preeclamptic pregnancy (blue). P-value using LME model is shown. PIGF and PAPP-A came as significant. **B.** FLT1/PIGF Ratio.

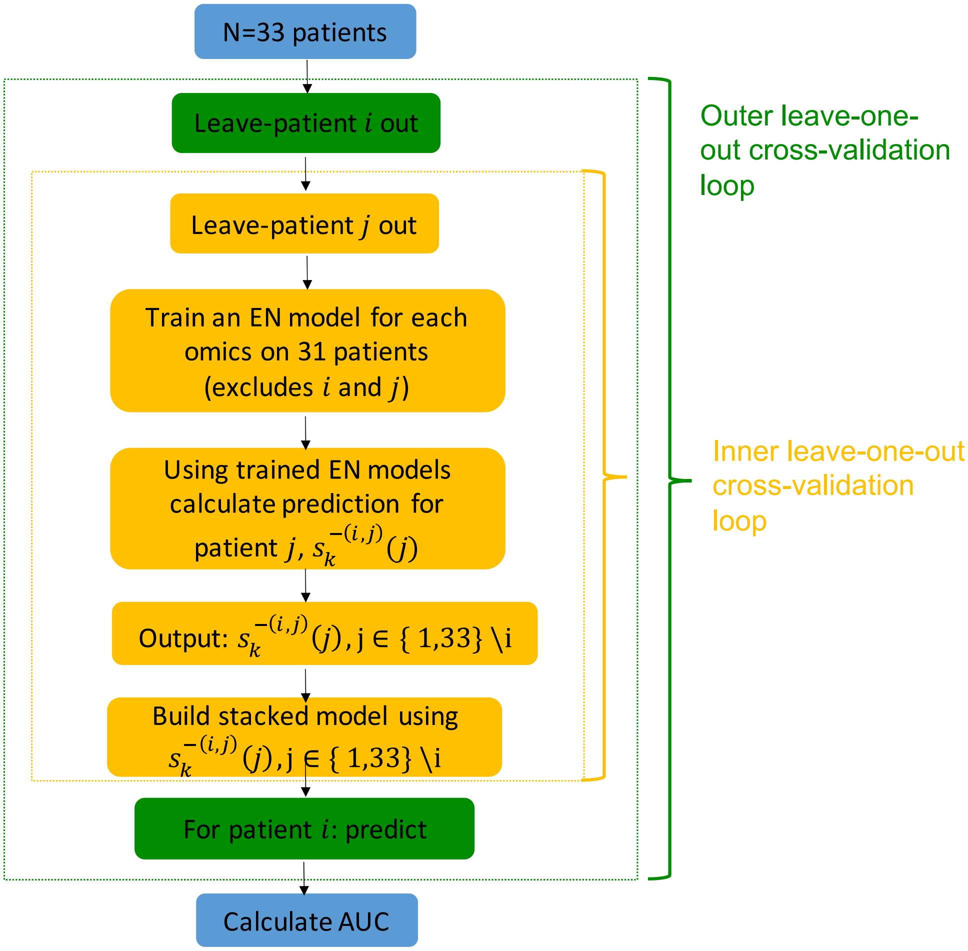

Figure S15. Integration using nested (two-step) cross-validation to build predictive model of preeclampsia using six omics datasets. In each step of cross-validation, EN models for each omics set are first trained and then the stacked model is trained in the same step. After the stacked model is built, it is tested on the test patient that was left out in the outer cross-validation loop. Therefore, no leakage of information between training and test data occurred.

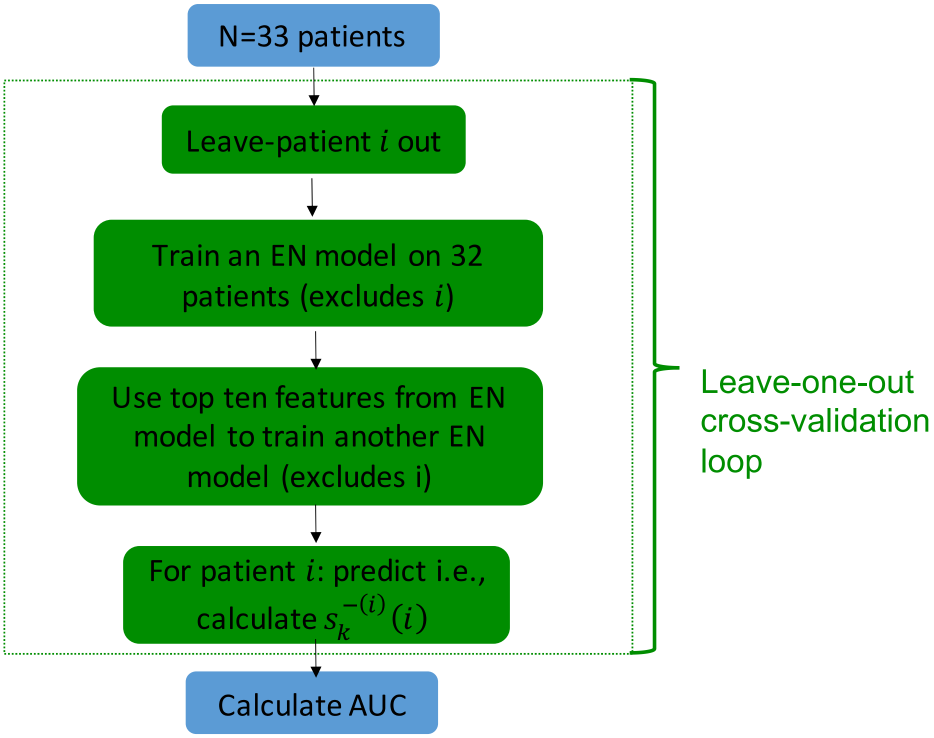

Figure S16. Algorithm for an EN prediction model using top ten features. In each cross-validation step, EN model is trained and then a regression model is trained based on ten features chosen by EN in the same step. The refitted model is then tested on the test patient that was left out in the cross-validation loop. Therefore, no leakage of information between training and test data occurred.

Supplemental Experimental Procedures

1. Cell-free RNA Transcriptome

Cell-free RNA (cfRNA) was extracted from 1 mL of plasma using Plasma/Serum Circulating RNA and Exosomal Purification kit (Norgen, cat 29500) following manufacturer instructions. Residual DNA was digested using BaselineZERO DNase (Epicentre) and then cleaned using RNA Clean and Concentrator-96 kit (Zymo). RNA was eluted to 12 ul in elution buffer. Libraries were prepared using 4 uL cfRNA and SMARTer Stranded Total RNAseq Kit v2 -Pico Input Mammalian Components (Clontech Cat No 634419) and SMARTer RNA Unique Dual Index Barcodes (Set A, Cat 634452) according to the manufacturer's manual. Short read sequencing was performed using the Illumina NovaSeq (2 × 75 bp) platform to an average depth of 50 million reads per

sample. Raw sequencing reads were trimmed with trimmomatic and then mapped to the human reference genome (hg38) with STAR. Duplicates were removed by Picard and then unique reads were quantified using htseq-count. Mapping quality statistics were aggregated using MultiQC.

To estimate RNA degradation, we first counted the number of reads per exon and annotated each exon with its corresponding gene ID and exon number using htseq-count. We then counted the number of genes for which all reads mapped exclusively to the 3' most exon per sample and divided by the total number of genes detected to obtain the fraction of genes where all reads mapped to the 3' most exon.

Finally, we estimated ribosomal read fraction by counting the number of reads that mapped to the ribosomal region (GL00220.1:105424-118780) using samtools view.

Dataset quality is described in the parallel work[17]. Briefly, for every sequenced sample, we estimated three quality parameters were estimated as previously described by our group[18,19]. Our final analysis included a subset of all samples that passed pre-defined quality cutoffs, empirically estimated based on ~700 previously sequenced cell-free RNA samples collected from 5 sites across the globe. Finally, we visualized sample quality as a function of the three defined metrics and find that low-quality samples both cluster separately using hierarchical clustering and drive variance using principal component analysis. Both visualizations and further details regarding quality metrics can be found in Moufarrej et. al (Main text, Methods, Fig S1,2)[17].

2. Proteome

Proteomic assay: Blood was collected into EDTA tubes, immediately placed in ice, centrifuged (3,000 rpm), and plasma was removed and transferred into 1.5-ml microfuge tubes. Tubes were then spun at 13,000 rpm for 1 min, plasma was transferred into another set of microfuge tubes and spun again for 1 min. Plasma was stored at -80°C. All processing was completed within 60 min of collection.

All proteomic analyses were performed blinded and in randomly allocated samples by SomaLogic, Inc. (Boulder, CO) using a highly multiplex aptamer-based platform [S20]. The assay quantifies relative concentrations of 1,310 proteins over a wide dynamic range (> 8 log) using chemically-modified aptamers with slow off-rate kinetics (SOMAmer reagents). Each SOMAmer reagent is a unique, high-affinity, single-strand DNA endowed with functional groups mimicking amino acid side chains. Nucleotide signals are quantified using relative florescence on microarrays (Agilent Technologies, Santa Clara, CA). The assay has a historic median intra- and inter-run coefficient of variation of about 5%, and median lower and upper limits of quantification of 3.0 pM and 1.5 nM[20].

Quality control at the sample level included the use of control SOMAmers on the microarray to monitor for differences in hybridization efficiency, and the calculation of the median signal over all SOMAmers to account for technical variability. The resulting hybridization and median scale factors were used for data normalization across samples. Acceptable scale factors ranged between 0.4 and 2.5 based on historic runs. Quality control at the SOMAmer level included the

use of replicate calibrator plasma samples (7) and biological controls (4) to monitor for repeatability and batch-to-batch variability. Historic values were used for each SOMAmer to derive a calibration scale factor. Acceptance criteria were a median scale factor between 0.8 and 1.2, and deviation by less than 0.4 from the plate median for 95% of SOMAmers. All quality metrics for the proteomic assay were met with plate scale factors of 1.24 and 1.46, and SOMAmer calibration factors < 0.4 for 95% of SOMAmers. The median coefficient of variation was 4.1%. A negligible number of proteins did not pass quality control (Fig S17).

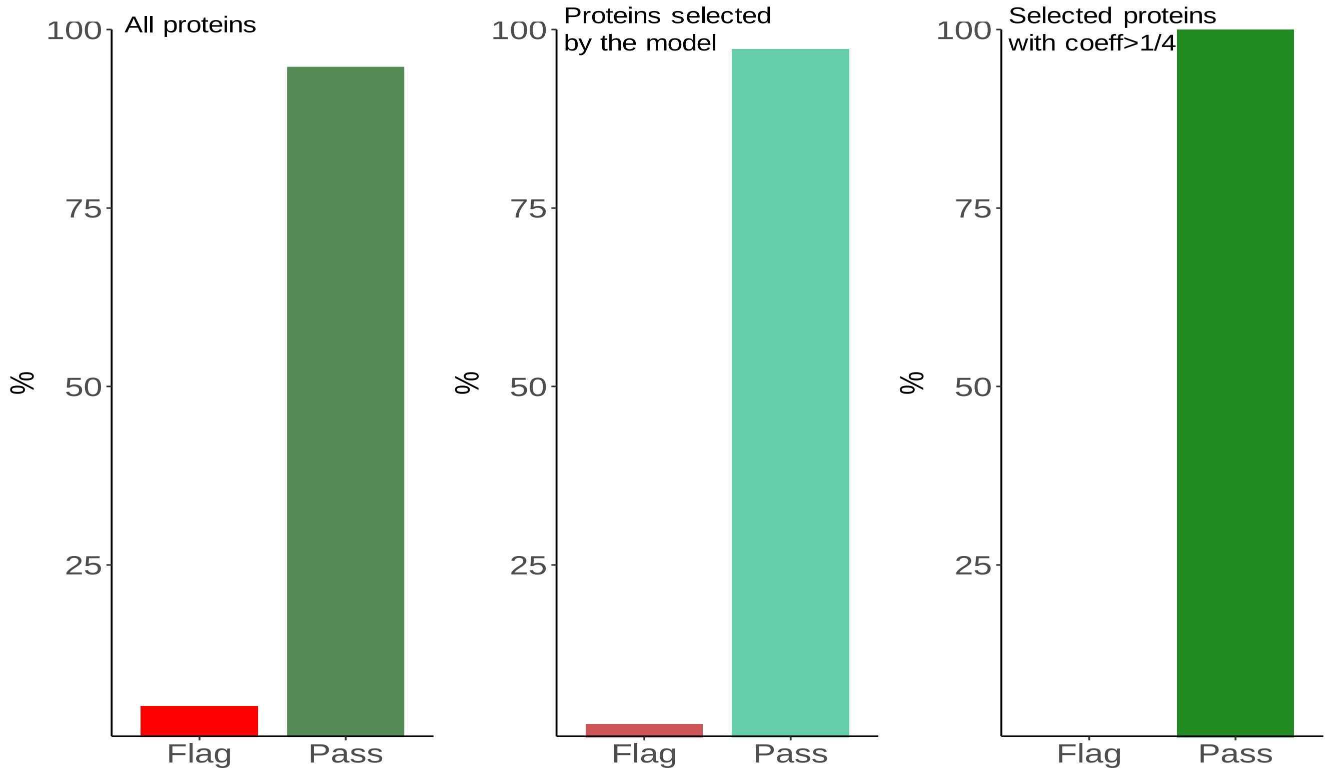

Figure S17. Quality analysis of proteome. Y-axis shows the percentage of proteins that passed the quality assessment.

We point out that SomaLogic aptamer technology used in this study have been previously extensively validated using orthogonal technologies (the enzyme-linked immunosorbent assay (ELISA) and Olink), multiple reaction monitoring mass spectrometry (MRM-MS), data dependent acquisition mass spectrometry (DDA-MS) and genetic strategies. Specifically,

studies that performed validation of proteins identified in our study are listed in Table S5 below.

**Table S5. Validation of aptamer assays for identified proteins.**			
**Protein**	**Orthogonal strategy**	**MS**	**Genetic Strategies**
LEP	Elisa[21]		
CXCL10	Elisa[22], Olink[23]		[23,24]
SELE	Olink[23]	MRM-MS[24]	[23,24]
SELL	Luminex[25], Olink[23]	DDA-MS[24]	[23,24]
APOB		MRM-MS[24], DDA-DS[24]	[24]
SPARCL1			[23,24]
PRSS2			[23]
ROR1			[24]

3. Microbiome

Self-sampling of the vagina was performed weekly by study participants. Sterile Catch-AllTM Sample Collection Swabs (Epicentre Biotechnologies, Madison, WI, USA) were used to obtain material from: vagina (midvaginal wall). All clinical specimens were placed immediately after collection at −20◦C until transport to the laboratory for storage at −80◦C until further processing. Whole genomic DNA was extracted from each vaginal swab by means of the PowerSoil DNA isolation kit (MO BIO Laboratories) according to the manufacturer's protocol except for the inclusion of a 10-min incubation at 65◦C immediately after the addition of solution C1. The V4 hypervariable region of the 16S rRNA gene was amplified by PCR. The forward PCR primer (50 AAT GAT ACG GCG ACC ACC GAG ATC TAC ACG CTN NNN NNN NNN NNT ATG GTA ATT GTG TGY CAG CMG CCG CGG TAA 30) was a 75-nucleotide (nt) fusion primer consisting of the 32-nt Illumina adapter (designated by bold), a unique 12-nt barcode to label each amplicon (designated by the N's), a 10-nt forward primer pad, a 2-nt linker (GT), and the 19-nt broad-range bacterial primer 515F (designated by underlining). The 56-nt reverse primer (5' CAA GCA GAA GAC GGC ATA CGA GAT AGT CAG CCA GCC GGA CTA

CNV GGG TWT CTA AT 30) consisted of the 24-nt Illumina adapter (designated by bold), a 10-nt reverse primer pad, a 2-nt reverse primer linker (CC), and the 20-nt broad-range bacterial primer 806R (designated by underlining). Triplicate 25-µL PCRs were carried out by using 1× HotMasterMix (5 PRIME), 0.4 µM concentrations of each commercially synthesized primer, and 3 µL of prepared DNA template. Thermal cycling conditions consisted of an initial denaturing step of 94◦C for 3 min, followed by 30 cycles of 94◦C for 45s, 50◦C for 60s, and 72◦C for 90s, with a final extension step of 72◦C for 10 minutes. Upon completion of the PCRs, the corresponding triplicate reaction mixtures were pooled and purified by using the Ultra-clean-htp 96-well PCR clean-up kit (Mo Bio Laboratories) according to the manufacturer's protocol. DNA concentrations from each triplicate pool were quantified using the QuantiT High-Sensitivity dsDNA Assay Kit (Invitrogen) and combined in equimolar
14 ratios into a single tube. The resulting amplicon mixture was concentrated by ethanol precipitation and resuspended in 100 µL of molecular biology-grade water (Life Technologies). The resuspended amplicon mixture was gel purified and recovered using a QIAquick gel extraction kit (Qiagen). Recovered PCR products were sequenced on an Illumina HiSeq 2500 instrument (Illumina) at the W. M. Keck Center for Comparative Functional Genomics at the University of Illinois, Urbana–Champaign, IL. Bioinformatics processing largely followed the DADA2 Workflow for Big Data (benjjneb.github.io/dada2/bigdata_paired.html). Forward/reverse read pairs were trimmed and filtered, with forward reads truncated at 245 nt and reverse reads at 235 nt, no ambiguous bases allowed, and each read required to have less than two expected errors based on their quality scores. The relationship between quality scores and error rates was estimated for each sequencing run to reduce batch effects arising from run-to-run variability. ASVs were

independently inferred from the forward and reverse of each sample using the run-specific error rates, and then read pairs were merged. Chimeras were identified in each sample, and ASVs were removed if identified as chimeric in a sufficient fraction of the samples in which they were present. Taxonomic assignment was performed against the Silva v123 database using the implementation of the RDP naive Bayesian classifier available in the dada2 R package[26]. Lactobacillus species were assigned by hand via BLAST against sequences from cultured Lactobacillus strains.

For the vaginal microbiome dataset, biodiversity coverage was nearly complete, as shown by rarefaction curves for each sample (Fig S18). The curves are asymptotic, suggesting that the sequencing depth was sufficient to exhaustively sample the biodiversity present, which was measured using amplicon sequence variants (ASVs). Note that it is common for vaginal microbiomes to have relatively low estimates of biodiversity in states of health, as shown in Figure S19, and for increased diversity to be associated with disease risk (e.g., preterm birth). Our reads have been submitted to SRA. The BioProject accession is PRJNA752652.

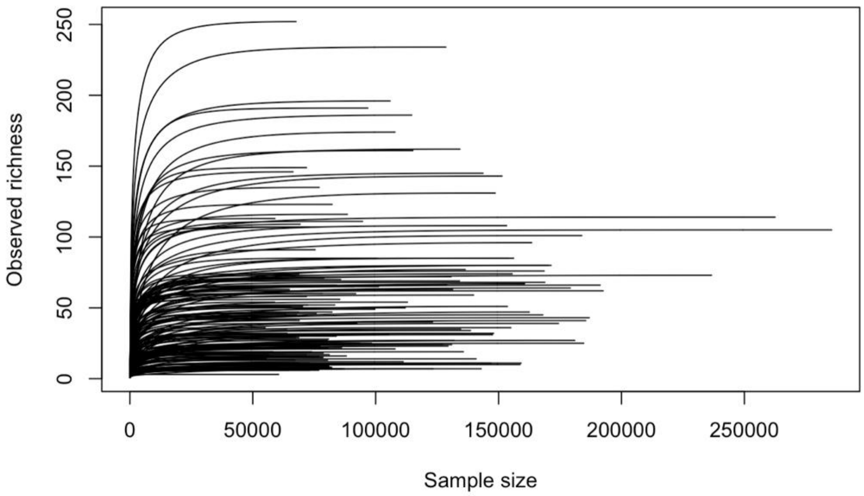

Figure S18. Rarefaction (coverage) curves for vaginal swabs analyzed for microbiome dataset, demonstrating nearly complete biodiversity coverage.

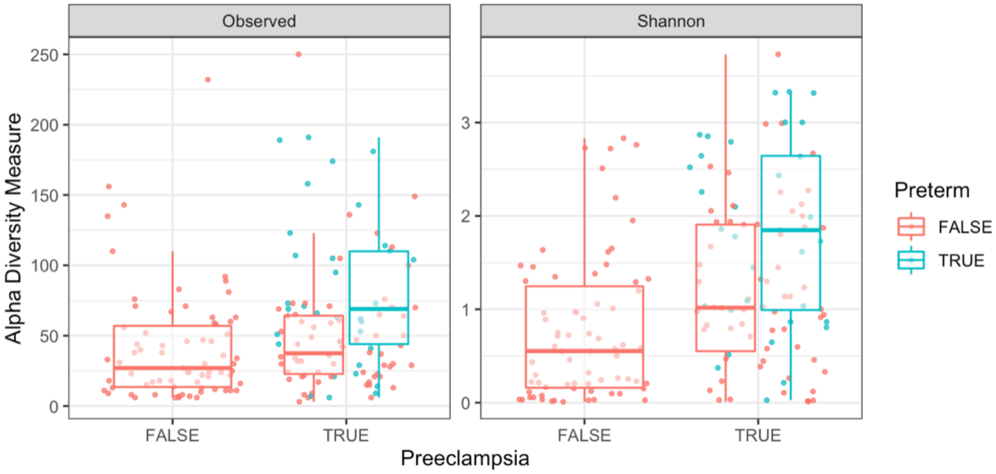

Figure S19. Alpha diversity estimates for vaginal swabs analyzed for microbiome dataset. These demonstrate that vaginal microbiomes have lower estimates of biodiversity in states of health and increased diversity to be associated with disease risk (e.g., preterm birth).

Principal Component Analysis (PCA) revealed that microbiome samples cluster together and no batch effects (Fig. S20).

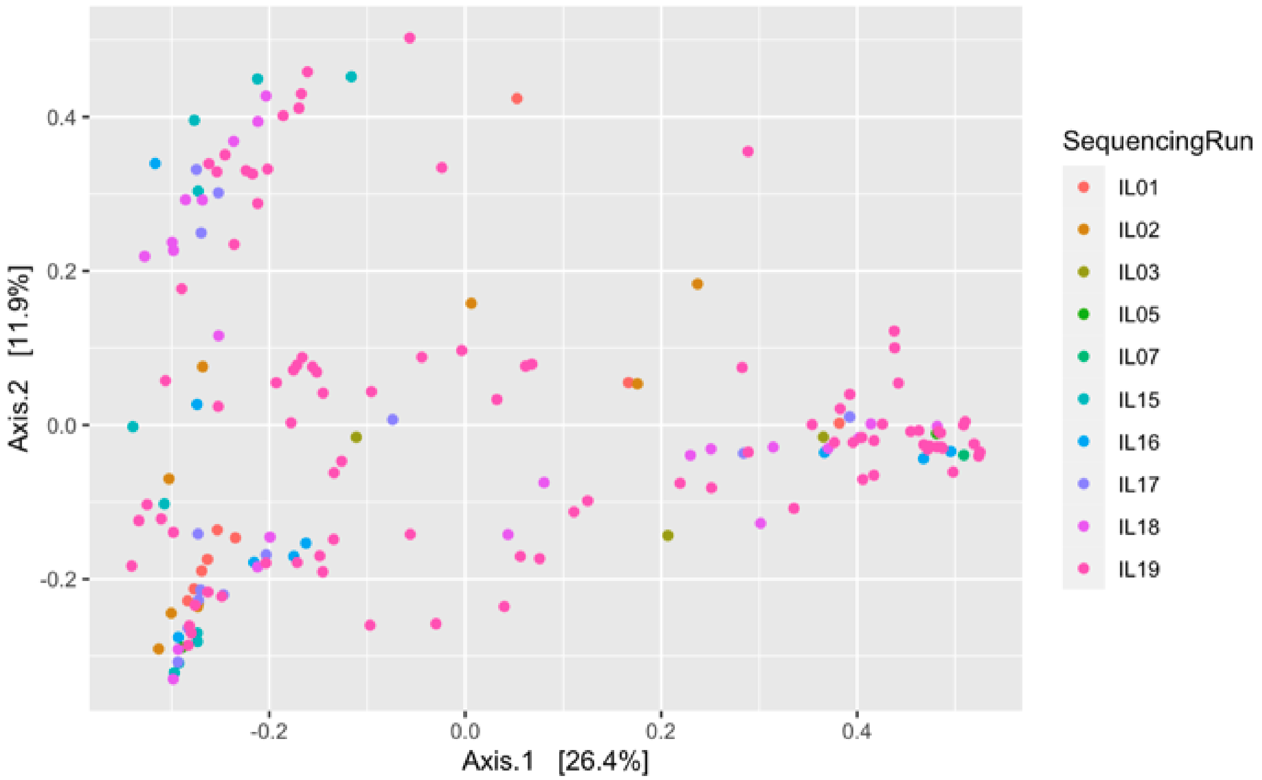

Figure S20: PCA of microbiome samples. Different sequencing runs are shown with different colors.

4. Immunome

Whole blood samples were stimulated for 15 min with either LPS, IFNα, a cocktail containing IL-2 and IL-6, or left unstimulated. Samples were then processed using a standardized protocol for fixation (SmartTube Inc), barcoding and antibody staining of whole blood samples for mass cytometry analysis[27]. For further details see[28]. Three categories of immune features were derived for integrative analysis: Cell frequency features: cell frequencies were expressed as a percentage of gated singlets in the case of neutrophils, and as a percentage of mononuclear cells (CD45+CD66−) in the case of all other cell types. Endogenous signaling immune features: Endogenous intracellular signaling

activities were derived from the analysis of unstimulated blood samples. The signal intensity of the following functional markers was simultaneously quantified per single cell: phospho (p) STAT1, pSTAT3, pSTAT5, pNFκB, total IκB, pMAPKAPK2, pP38, prpS6, pERK1/2, and pCREB. For each cell type, signaling immune features were calculated as the median signal intensity (arcsinh transformed value) of each signaling protein. Intracellular signaling response features: the signal intensity of all functionalmarkers was analyzed from samples stimulated with LPS, IFNα or IL. For each cell type, signaling responses were calculated as the difference in median signal intensity (arcsinh transformed value) of each signaling protein between the stimulated and unstimulated conditions.

5. Metabolomics and Lipidomics Analyses

While lipidome can be considered a part of the metabolome, in this study, we consider them separately because the datasets are generated using a very different workflow. Also, in this study lipidome refers to complex lipids, whereas small lipids such as fatty acids, oxylipins, etc. are part of our metabolome data.

Untargeted Metabolomics by Liquid Chromatography (LC)- Mass Spectrometry (MS)

LC-MS-grade solvents and mobile phase modifiers were obtained from Fisher Scientific (water, acetonitrile, methanol) and Sigma−Aldrich (acetic acid, ammonium acetate). Urine and plasma samples were analyzed using a broad-spectrum metabolomics platform consisting of hydrophilic interaction chromatography (HILIC) and reverse phase liquid chromatography (RPLC)–MS[29].

<u>Sample preparation</u>. Frozen urine samples were thawed on ice and centrifuged at 17,000g for 10 min at 4°C. Supernatants (25 µl) were then diluted 1:4 with 75% acetonitrile and 100% water for

HILIC- and RPLC-MS experiments, respectively. Samples for HILIC-MS experiments were further centrifuged at 21,000g for 10 min at 4°C to precipitate proteins. Frozen plasma samples were thawed on ice and metabolites were prepared from 100 µl of plasma using 1:1:1 acetone:acetonitrile:methanol, evaporated to dryness under nitrogen, and reconstituted in 1:1 methanol:water. Each sample was spiked-in with 15 analytical-grade internal standards (IS).

Data acquisition. Metabolic extracts were analyzed using HILIC and RPLC separations in both positive and negative ionization modes. Data were acquired on a Thermo Q Exactive HF mass spectrometer equipped with a Heated Electrospray Ionization probe (HESI-II) and operating in full MS scan mode. MS/MS data were acquired at different fragmentation energies (NCE 25, 35 and 50) on pooled samples consisting of an equimolar mixture of all the samples in the study. HILIC experiments were performed using a ZIC-HILIC column 2.1 x 100 mm, 3.5 µm, 200Å (Merck Millipore) and mobile phase solvents consisting of 10 mM ammonium acetate in 50/50 acetonitrile/water (A) and 10 mM ammonium acetate in 95/5 acetonitrile/water (B). RPLC experiments were performed using a Zorbax SBaq column 2.1 x 50 mm, 1.7 µm, 100Å (Agilent Technologies) and mobile phase solvents consisting of 0.06% acetic acid in water (A) and 0.06% acetic acid in methanol (B).

Data quality was ensured by: (1) sample randomization for metabolite extraction and data acquisition, (2) multiple injections of a pooled sample to equilibrate the LC-MS system prior to running the sequence (12 and 6 injections for HILIC and RPLC methods, respectively), (3) spike-in labeled IS during sample preparation to control for extraction efficiency and evaluate LC-MS performance, (4) checking mass accuracy, retention time and peak shape of the IS in each sample and (5) injection of a pooled sample every 10 injections to control for signal deviation over time.

Data processing. Data from each mode were independently processed using Progenesis QI software (v2.3) (Nonlinear Dynamics). Metabolic features from blanks and that did not show sufficient linearity upon dilution in QC samples ($r < 0.6$) were discarded. Only metabolic features present in > 2/3 of the samples were kept for further analysis. Inter- and intra-batch variations were corrected by applying locally estimated scatterplot smoothing local regression (LOESS) on pooled samples injected repetitively along the batches (span = 0.75). Dilution effects for urine samples were corrected using probabilistic quotient normalization (PQN). Missing values were imputed by drawing from a random distribution of low values in the corresponding sample. Data from each mode were then merged, producing a dataset containing 8718 and 3622 metabolic features for urine and plasma, respectively. Metabolite abundances were reported as spectral counts.

Metabolic feature annotation. Peak annotation was first performed by matching experimental m/z, retention time and MS/MS spectra to an in-house library of analytical-grade standards. Remaining peaks were identified by matching experimental m/z and fragmentation spectra to publicly available databases including HMDB (http://www.hmdb.ca/), MoNA (http://mona.fiehnlab.ucdavis.edu/) and MassBank (http://www.massbank.jp/) using the R package 'metID' (v0.2.0)[30]. Briefly, metabolic feature tables from Progenesis QI were matched to fragmentation spectra with a m/z and a retention time window of ± 15 ppm and ± 30 s (HILIC) and ± 20 s (RPLC), respectively. When multiple MS/MS spectra match a single metabolic feature, all matched MS/MS spectra were used for the identification. Next, MS1 and MS2 pairs were searched against public databases and a similarity score was calculated using the forward dot–product algorithm which considers both fragments and intensities. Metabolites were reported if the similarity score was above 0.4. We used the Metabolomics Standards Initiative (MSI) level of

confidence to grade metabolite annotation confidence (level 1 - level 4). Level 1 represents formal identifications where the biological signal matches accurate mass, retention time and fragmentation spectra of an authentic standard run on the same platform. For level 2 identification, the biological signal matches accurate mass and fragmentation spectra available in one of the public databases listed above. Level 3 represents putative identifications that are the most likely name based on previous knowledge of blood and urine composition. Level 4 consists in unknown metabolites. Annotated urine metabolites selected in the prediction models are reported in Table S3.

PCA revealed that samples cluster together and the absence of batch effects (Fig. S21), thereby confirming satisfactory quality control.

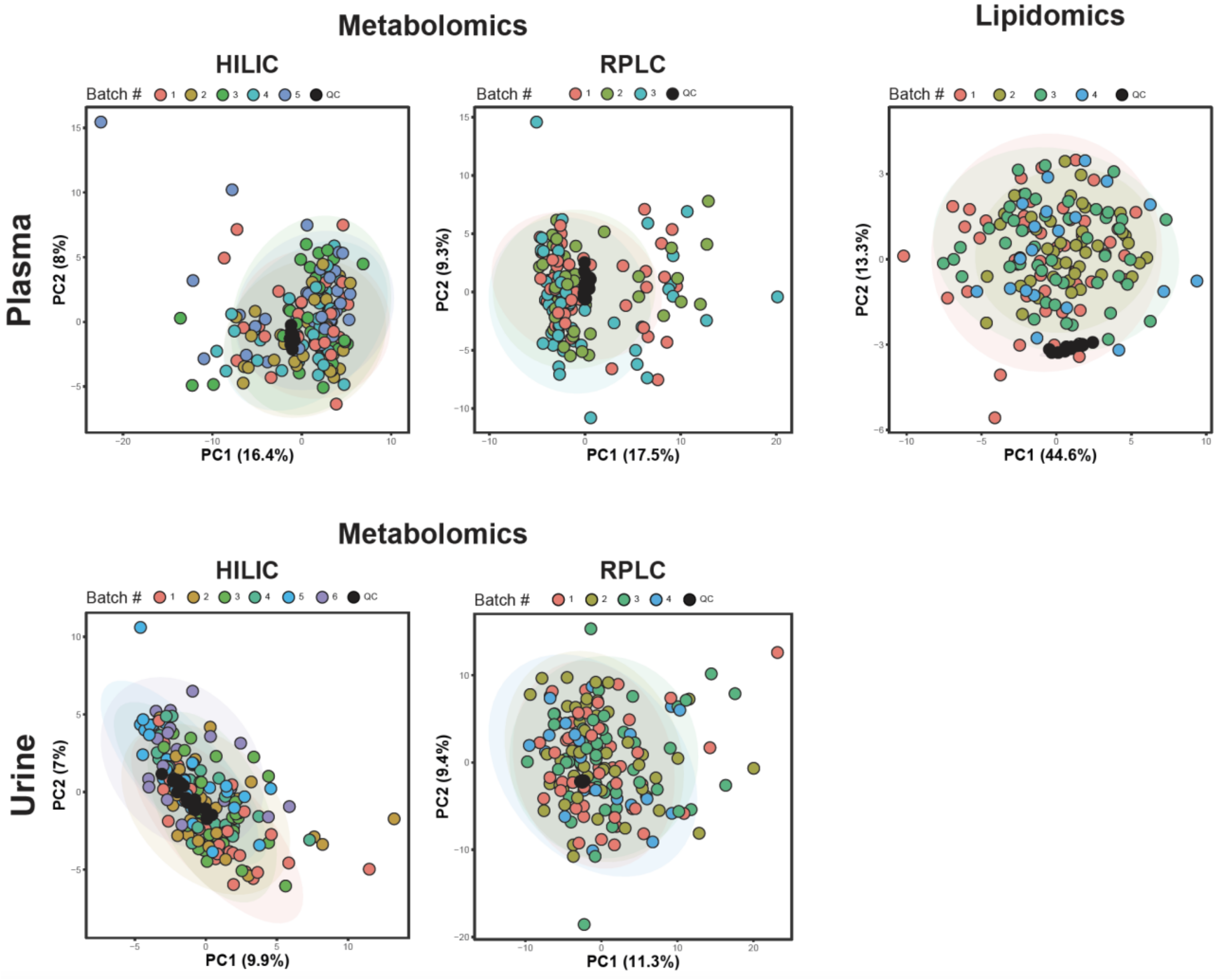

Figure S21: PCA of metabolome plasma, metabolome urine and lipidome samples. Different colors are assigned to different batches.

Comparison between enrichment factors and statistical significance of pathways enriched over gestation versus in early pregnancy is shown in Figure S22.

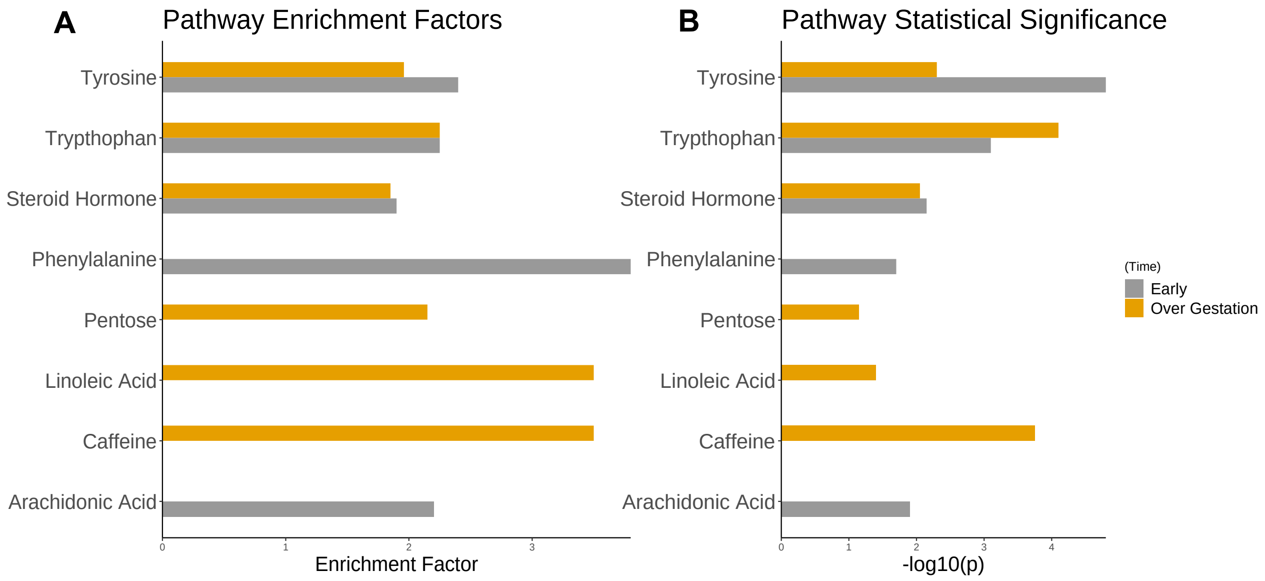

Figure S22. Pathways enriched over gestation (yellow) and early in pregnancy (grey). A. Pathway enrichment. **B.** Statistical significance.

References

1. Taylor BD, Ness RB, Olsen J, Hougaard DM, Skogstrand K, Roberts JM, Haggerty CL. leptin measured in early pregnancy is higher in women with preeclampsia compared with normotensive pregnant women. Hypertension 65, 594–599 (2015).
2. Pérez-Pérez A, Toro A, Vilariño-García T, Maymó J, Guadix P, Dueñas JL, Fernández-Sánchez M, Varone C, Sánchez-Margalet V.Leptin action in normal and pathological pregnancies. J. Cell Mol. Med. 22, 716–727 (2018).
3. Naylor, C. & Petri, W. A. Leptin regulation of immune responses. Trends Mol. Med. 22, 88–98 (2016).
4. Abella V, Scotece M, Conde J, Pino J, Gonzalez-Gay MA, Gómez-Reino JJ, Mera A, Lago F, Gómez R, Gualillo O. Leptin in the interplay of inflammation, metabolism and immune system disorders. Nat. Rev. Rheumatol. 13, 100–109 (2017).
5. Martín-Romero, C., Santos-Alvarez, J., Goberna, R. & Sánchez-Margalet, V. Human leptin enhances activation and proliferation of human circulating T lymphocytes. Cell Immunol. 199, 15–24 (2000).
6. Maynard, S. E. & Karumanchi, S. A. Angiogenic factors and preeclampsia. Semin Nephrol 31, 33–46 (2011).
7. Rath, G. & Tripathi, R. Angiogenic balance and diagnosis of pre-eclampsia: selecting the right VEGF receptor. J Hum Hypertens 26, 207–210 (2012).
8. Docheva N, Romero R, Chaemsaithong P, Tarca AL, Bhatti G, Pacora P, Panaitescu B, Chaiyasit N, Chaiworapongsa T, Maymon E, Hassan SS, Erez O.The profiles of soluble adhesion molecules in the "great obstetrical syndromes". J. Matern. Fetal Neonatal Med. 32, 2113–2136 (2019).
9. Ivetic, A., Hoskins Green, H. L. & Hart, S. J. L-selectin: A Major Regulator of Leukocyte Adhesion, Migration and Signaling. Front. Immunol. 10, 1068 (2019).
10. Seidelin, J. B., Nielsen, O. H. & Strøm, J. Soluble L-selectin levels predict survival in sepsis. Intensive Care Med. 28, 1613–1618 (2002).
11. Rainer, T. H. L-selectin in health and disease. Resuscitation 52, 127–141 (2002).
12. Chen J, Yue C, Xu J, Zhan Y, Zhao H, Li Y, Ye Y. Downregulation of receptor tyrosine kinase-like orphan receptor 1 in preeclampsia placenta inhibits human trophoblast cell proliferation, migration, and invasion by PI3K/AKT/mTOR pathway accommodation. Placenta 82, 17–24 (2019).
13. Gotsch F, Romero R, Friel L, Kusanovic JP, Espinoza J, Erez O, Than NG, Mittal P, Edwin S, Yoon BH, . et al. CXCL10/IP-10: a missing link between inflammation and anti-angiogenesis in preeclampsia? J. Matern. Fetal Neonatal Med. 20, 777–792 (2007).
14. Løset M, Mundal SB, Johnson MP, Fenstad MH, Freed KA, Lian IA, Eide IP, Bjørge L, Blangero J, Moses EK, Austgulen R. A transcriptional profile of the decidua in preeclampsia. Am. J. Obstet. Gynecol. 204, 84.e1-27 (2011).
15. Ma, H. Y., Cu, W., Sun, Y. H. & Chen, X. MiRNA-203a-3p inhibits inflammatory response in preeclampsia through regulating IL24. Eur Rev Med Pharmacol Sci 24, 5223–5230 (2020).
16. Zhang, Y., Cao L., Jia J., Ye L., Wang Y., Zhou B., Zhou R. CircHIPK3 is decreased in preeclampsia and affects migration, invasion, proliferation, and tube formation of human trophoblast cells. Placenta 85, 1–8 (2019).
17. Moufarrej M.N., Vorperian S.K., Wong R.J., Campos A.A., Quaintance C.C., Sit R.V., Tan M., Detweiler A.M., Mekonen H., Neff N.F., Baruch-Gravett C., Litch J.A., Druzin M.L., Winn V.D.,

Shaw G.M., Stevenson D.K., Quake S.R. Early prediction of preeclampsia in pregnancy with cell-free RNA. Nature. 2022 Feb;602(7898):689-694. doi: 10.1038/s41586-022-04410-z. Epub 2022 Feb 9. PMID: 35140405; PMCID: PMC8971130.

18. Pan W., Development of diagnostic methods using cell-free nucleic acids. Stanford University, 2016.
19. Moufarrej M., Wong R.J., Shaw G.M., Stevenson D.K., Quake S.R. Investigating Pregnancy and Its Complications Using Circulating Cell-Free RNA in Women's Blood During Gestation, Front. Pediatr., 2020.
20. Gold, L., Ayers D., Bertino J., Bock C., Bock A., Brody E.N., Carter J., Dalby A.B., Eaton B.E., Fitzwater T., *et al.* Aptamer-based multiplexed proteomic technology for biomarker discovery. *PLoS One* **5,** e15004 (2010).
21. Anderson, J., Seol H., Gordish-Dressman H., Hathout Y., Spurney C.F. Interleukin 1 Receptor-Like 1 Protein (ST2) is a Potential Biomarker for Cardiomyopathy in Duchenne Muscular Dystrophy. *Pediatr. Cardiol.* **38,** 1606–1612 (2017).
22. Bodewes, I. L. A., Van der Spek P.J., Leon L.G., Wijkhuijs A.J.M., Van Helden-Meeuwsen C.G., Tas L., Schreurs M.W.J., Van Daele P.L.A., Katsikis P.D., Versnel M.A. Fatigue in Sjögren's syndrome: A search for biomarkers and treatment targets. *Front. Immunol.* **10,** (2019).
23. Sun, B. B., Maranville J.C., Peters J.E., Stacey D., Staley J.R., Blackshaw J., Burgess S., Jiang T., Paige E., Surendran P., *et al.* Genomic atlas of the human plasma proteome. *Nature* **558,** 73–79 (2018).
24. Emilsson, V., Ilkov M., Lamb J.R., Finkel N., Gudmundsson E.F., Pitts R., Hoover H., Gudmundsdottir V., Horman S.R., Aspelund T. *et al.* Co-regulatory networks of human serum proteins link genetics to disease. *Science* **361,** 769–773 (2018).
25. Giudice, V., Biancotto A., Wu Z., Cheung F., Candia J., Fantoni G., Kajigaya S., Rios O., Townsley D., Feng X., Young N.S. Aptamer-based proteomics of serum and plasma in acquired aplastic anemia. *Exp. Hematol.* **68,** 38–50 (2018).
26. Wang Q., Garrity G.M., Tiedje J.M., Cole J.R. Naive Bayesian classifier for rapid assignment of rRNA sequences into the new bacterial taxonomy. Appl Environ Microbiol. 2007 Aug;73(16):5261-7. doi: 10.1128/AEM.00062-07. Epub 2007 Jun 22. PMID: 17586664; PMCID: PMC1950982.
27. Bodenmiller, B., Zunder, E., Finck, R., Chen T.J., Savig E.S., Bruggner R.V., Simonds E.F., Bendall S.C., Sachs K., Krutzik P.O., Nolan G.P. Multiplexed mass cytometry profiling of cellular states perturbed by small-molecule regulators. *Nat Biotechnol* **30**, 858–867 (2012). https://doi.org/10.1038/nbt.2317
28. Aghaeepour N., Ganio EA, Mcilwain D., Tsai A.S., Tingle M., Van Gassen S., Gaudilliere D.K., Baca Q., McNeil L., Okada R., Ghaemi M.S., Furman D., Wong R.J., Winn V.D., Druzin M.L., El-Sayed Y.Y. *et.al.*, B. An immune clock of human pregnancy. Sci Immunol. 2017 Sep 1;2(15):eaan2946. doi: 10.1126/sciimmunol.aan2946. PMID: 28864494; PMCID: PMC5701281.
29. Contrepois K., Jiang L., Snyder M. Optimized Analytical Procedures for the Untargeted Metabolomic Profiling of Human Urine and Plasma by Combining Hydrophilic Interaction (HILIC) and Reverse-Phase Liquid Chromatography (RPLC)-Mass Spectrometry. Mol Cell Proteomics. 2015 Jun;14(6):1684-95. doi: 10.1074/mcp.M114.046508. Epub 2015 Mar 18. PMID: 25787789; PMCID: PMC4458729.
30. Shen, X., Wang R., Xiong X., Yin Y., Cai Y., Ma Z., Liu N., Zhu Z-J. Metabolic reaction network-based recursive metabolite annotation for untargeted metabolomics. *Nat. Commun.* **10,**

1516 (2019).
