# Supplementary material for: Early prediction and longitudinal modeling of preeclampsia from multiomics
Source: Patterns (N Y). 2022 Dec 9;3(12):100655. doi: 10.1016/j.patter.2022.100655 (PMC9768681; doi:10.1016/j.patter.2022.100655)
Supplement: Document S2. Article plus supplemental information [file mmc2.pdf]

Patterns

Article

Early prediction and longitudinal modeling of preeclampsia from multiomics

Highlights

- Machine-learning models for prediction of preeclampsia were developed
- Six omics datasets from a longitudinal cohort of pregnant women were analyzed
- Prediction models from urine metabolome and from proteome had the best accuracy
- The prediction model from urine metabolites was validated on an independent cohort

Authors

Ivana Marić, Kévin Contrepois, Mira N. Moufarrej, ..., David K. Stevenson, Brice Gaudilliere, Nima Aghaeepour

Correspondence

ivanam@stanford.edu

In brief

Preeclampsia is one of the main complications of pregnancy, posing risk both to the mother and the baby. We developed machine-learning models for early prediction of preeclampsia (first 16 weeks of pregnancy) and over gestation by analyzing six omics datasets from a longitudinal cohort of pregnant women. If further validated, our findings could lead to a simple prediction test for use in both developed and developing parts of the world.

Marić et al., 2022, Patterns *3*, 100655
December 9, 2022 © 2022 The Authors.
https://doi.org/10.1016/j.patter.2022.100655

CellPress

Patterns

CellPress
OPEN ACCESS

Article

Early prediction and longitudinal modeling of preeclampsia from multiomics

Ivana Marić,[1,12,14,*] Kévin Contrepois,[2,12] Mira N. Moufarrej,[5] Ina A. Stelzer,[3] Dorien Feyaerts,[3] Xiaoyuan Han,[8] Andy Tang,[3] Natalie Stanley,[3] Ronald J. Wong,[1] Gavin M. Traber,[2] Mathew Ellenberger,[2] Alan L. Chang,[3] Ramin Fallahzadeh,[3] Huda Nassar,[3] Martin Becker,[3] Maria Xenochristou,[3] Camilo Espinosa,[3] Davide De Francesco,[3] Mohammad S. Ghaemi,[3,10] Elizabeth K. Costello,[9] Anthony Culos,[3] Xuefeng B. Ling,[7] Karl G. Sylvester,[7] Gary L. Darmstadt,[1] Virginia D. Winn,[4] Gary M. Shaw,[1] David A. Relman,[9,11,13] Stephen R. Quake,[5,13] Martin S. Angst,[3,13] Michael P. Snyder,[2,13] David K. Stevenson,[1,13] Brice Gaudilliere,[1,3,13] and Nima Aghaeepour[1,3,6,13]

[1]Department of Pediatrics, Division of Neonatal and Developmental Medicine, Stanford University School of Medicine, Stanford, CA 94305, USA
[2]Department of Genetics, Stanford University School of Medicine, Stanford, CA 94305, USA
[3]Department of Anesthesiology, Perioperative and Pain Medicine, Stanford University School of Medicine, Stanford, CA 94305, USA
[4]Department of Obstetrics and Gynecology, Stanford University School of Medicine, Stanford, CA 94305, USA
[5]Departments of Bioengineering and Applied Physics, Stanford University and Chan Zuckerberg Biohub, Stanford, CA 94305, USA
[6]Department of Biomedical Data Science, Stanford University, Stanford, CA 94305, USA
[7]Department of Surgery, Stanford University School of Medicine, Stanford, CA 94305, USA
[8]University of the Pacific, Arthur A. Dugoni School of Dentistry, San Francisco, CA 94103, USA
[9]Departments of Medicine, and of Microbiology & Immunology, Stanford University School of Medicine, Stanford, CA 94305, USA
[10]Digital Technologies Research Centre, National Research Council Canada, Toronto, Canada
[11]Infectious Diseases Section, Veterans Affairs Palo Alto Health Care System, Palo Alto, CA 94304, USA
[12]These authors contributed equally
[13]These authors contributed equally
[14]Lead contact
*Correspondence: ivanam@stanford.edu
https://doi.org/10.1016/j.patter.2022.100655

THE BIGGER PICTURE The World Health Organization estimates that more than 800 women worldwide die from pregnancy-related causes every day. One of the main causes is a hypertensive disorder, preeclampsia, for which the only treatment is to deliver, often too early. Preeclampsia affects 3%–5% of pregnancies in the United States and up to 8% globally. Machine-learning analyses of high-dimensional multiomics data could potentially capture complex dynamics involved in the preeclampsia pathogenesis. We developed machine-learning models for early prediction of preeclampsia (first 16 weeks of pregnancy) and over gestation by analyzing six omics datasets from a longitudinal cohort of pregnant women. A prediction model using nine urine metabolites had high accuracy and was validated on an independent cohort. While encouraging, our results need to be validated on a larger cohort. If generalizable, our findings could lead to a simple prediction test for use in both developed and developing parts of the world.

1 2 3 4 5 **Proof-of-Concept:** Data science output has been formulated, implemented, and tested for one domain/problem

SUMMARY

Preeclampsia is a complex disease of pregnancy whose physiopathology remains unclear. We developed machine-learning models for early prediction of preeclampsia (first 16 weeks of pregnancy) and over gestation by analyzing six omics datasets from a longitudinal cohort of pregnant women. For early pregnancy, a prediction model using nine urine metabolites had the highest accuracy and was validated on an independent cohort (area under the receiver-operating characteristic curve [AUC] = 0.88, 95% confidence interval [CI] [0.76, 0.99] cross-validated; AUC = 0.83, 95% CI [0.62,1] validated). Univariate analysis demonstrated statistical significance of identified metabolites. An integrated multiomics model further improved accuracy (AUC = 0.94). Several biological pathways were identified including tryptophan, caffeine, and arachidonic

This is an open access article under the CC BY license (http://creativecommons.org/licenses/by/4.0/).

acid metabolisms. Integration with immune cytometry data suggested novel associations between immune and proteomic dynamics. While further validation in a larger population is necessary, these encouraging results can serve as a basis for a simple, early diagnostic test for preeclampsia.

INTRODUCTION

The World Health Organization estimates that more than 800 women worldwide die from pregnancy-related causes every day, with the highest rates of maternal mortality and morbidity in low-income countries.[1] One of the main causes is a hypertensive disorder of pregnancy, preeclampsia, for which the only treatment is to deliver, often too early. Preeclampsia affects 3%–5% of pregnancies in the United States and up to 8% of all pregnancies globally,[1] and accounts for 10%–15% of maternal deaths[2] and 15%–20% of preterm births.[3]

The pathophysiology of preeclampsia is complex and is thought to be caused in part by abnormal placentation as well as a woman's genetic and immunologic predisposition.[4] It is believed that abnormal placentation leads to a maternal inflammatory response.[4] Placental ischemia, oxidative stress, and the presence of a maternal angiogenic imbalance are all characteristics of preeclampsia,[5,6] leading to endothelial and end-organ damage, and in some cases to stroke and even death.

Specific biological processes involved in the development of preeclampsia are not yet completely understood. Early prediction of preeclampsia has remained a clinical challenge, owing to incompletely understood causes, various risk factors, and likely multiple pathogenic phenotypes of preeclampsia.[7,8] The recent availability of high-throughput omics (e.g., genome, transcriptome, proteome, and metabolome) assays, where each can be performed on small sample volumes, has enabled joint analyses of the high-dimensional multidomain or "multiomics" data measured from the same biological sample.[4,9,10] An integrated analysis may capture complex dynamics involved in the preeclampsia which could ultimately lead to novel therapeutic interventions. Furthermore, applying machine-learning methods capable of extracting the most predictive features from high-dimensional multiomics data could lead to more accurate predictive models, discovery of biomarkers, and improved early detection of women at risk for developing preeclampsia.

In this study, we performed a multiomics analysis of the transcriptome, proteome, metabolome, lipidome, and microbiome from a coordinated set of biospecimens collected longitudinally from normotensive and preeclamptic pregnant women; we then integrated immune system mass spectrometry features that were available for a subset of the women; and finally, we combined the multiomics data with the available clinical/demographics data and performed a joint analysis. Our goals were to: (1) build an early prediction model of preeclampsia; (2) develop a simple and interpretable predictive model based on a small number of biomarkers that can lead to the development of a diagnostic test; (3) compare prediction capabilities of different omics sets; (4) build an integrated multiomics predictive model of preeclampsia to identify a signature of preeclampsia; and (5) gain insights into pathways involved in the pathogenesis of preeclampsia.

RESULTS

Study design and multiomics data collection

Our prospective study included 33 women in the discovery cohort (17 preeclamptic, 16 normotensive) and 16 women in the validation cohort (12 preeclamptic, 4 normotensive) (Figure 1A). The validation cohort was used to validate the metabolomics results. Among the preeclamptic women in the discovery cohort, severe and mild preeclampsia were observed in 10 and 7 women, respectively; early- and late-onset preeclampsia were observed in 5 and 12 women, respectively (Table S2). Maternal characteristics, demographics, and gestational ages at delivery are shown in Table S1. Both in discovery and validation cohorts there was a higher prevalence of chronic hypertension, high body mass index (BMI), and twin pregnancies—all known risks for preeclampsia—among preeclamptic compared with normotensive women (Table S1).

Blood, urine, and vaginal swabs were collected longitudinally at two or three time points during pregnancy: early, mid, and late (Figure 1). Across the gestation, we found no significant difference in sampling time between preeclamptic and normotensive groups ($p > 0.74$ first sample, $p > 0.6$ second sample, and $p > 0.3$ third sample; Wilcoxon rank-sum test). These samples were used for measurements of six omics assays: cell-free RNA (cfRNA)/transcriptome (plasma), proteome (plasma), metabolome (plasma and urine), lipidome (plasma), and microbiome (vaginal swab). In addition, immune-system-wide mass cytometry measurements of single cells were obtained on a subset of 19 women from the same cohorts (18 women from the discovery cohort and one woman from the validation cohort). The number of measurements differed markedly among omics datasets, with transcriptome containing the highest number of measurements (Figure S1A). In contrast, the number of principal components explaining 90% of the variance, which quantifies the internal correlation of a dataset, exhibited a smaller difference among datasets (Figure S1B). Thus, although the amount of data varied several orders of magnitude among datasets, their numbers of principal components were much more similar.

Prediction of preeclampsia in early pregnancy

From a clinical perspective, early prediction of preeclampsia, i.e., within the first 16 weeks of gestation, is of critical importance, as it would enable: early treatment of high-risk women (e.g., with low-dose aspirin[11]); closer monitoring of high-risk pregnancies; and the enrichment of preemptive interventional studies in women at risk for developing preeclampsia.[12] Identifying a small number of specific biomarkers that are predictive of preeclampsia early in pregnancy could ultimately facilitate the development of a simple and affordable diagnostic test for both high-income and low- and middle-income countries. To this end, we developed an early prediction model for preeclampsia using only samples collected from each omics dataset during the first 16 weeks of pregnancy. To agnostically examine all the

CellPress
OPEN ACCESS

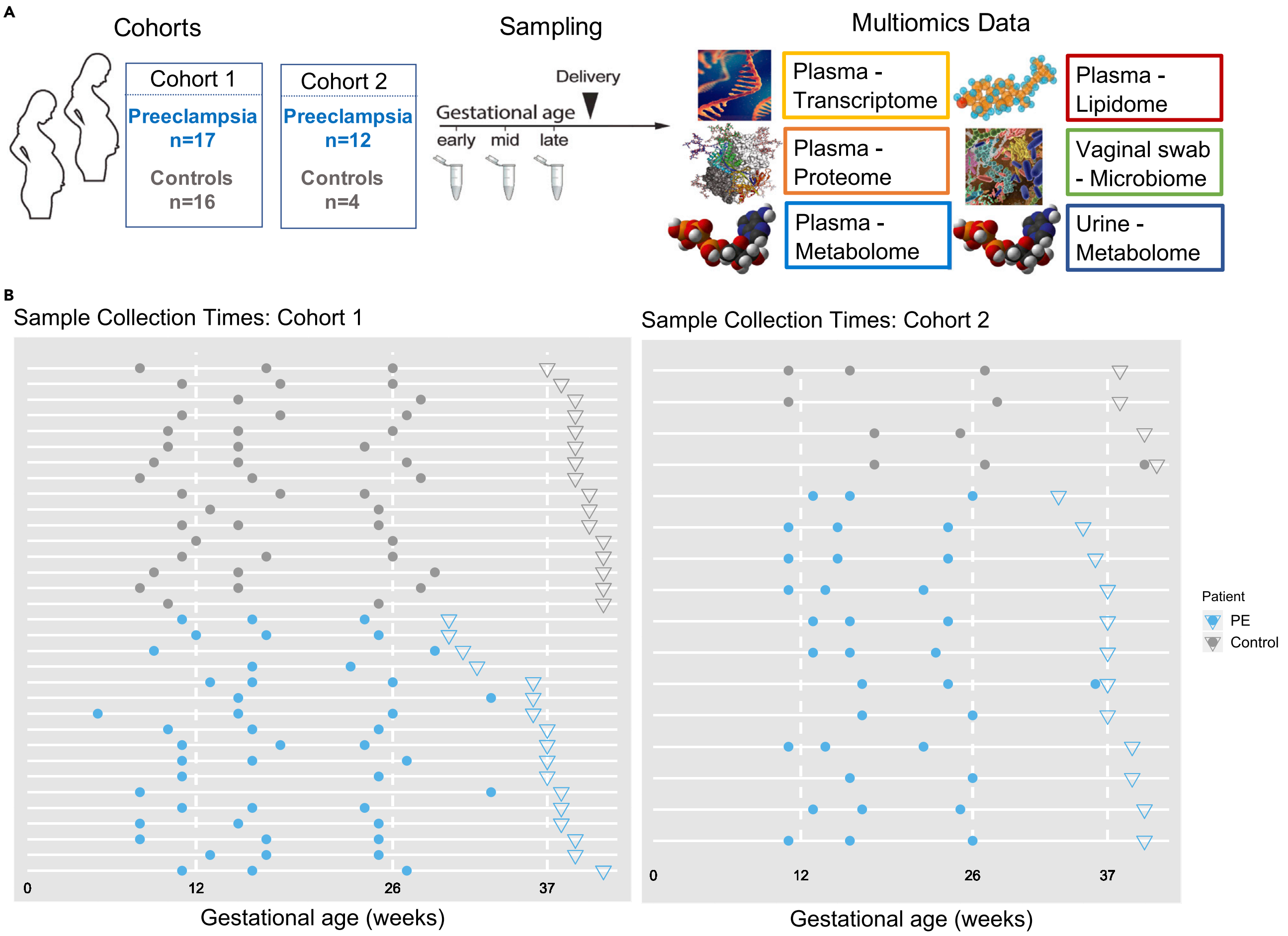

Figure 1. Overview of the study

(A) Two independent cohorts were analyzed using six different omics.

(B) Sample collection timeline for plasma in our discovery and validation cohorts. Circles indicate pre-delivery sample collection times, and inverted triangles indicate delivery dates for individual women (one per horizontal line).

measurements in our high-dimensional data, we used Elastic Net (EN), a regularized regression machine-learning method (see experimental procedures). EN was chosen for its ability to extract, from high-dimensional data, a handful of the most predictive features that can predict an outcome with high accuracy.[13] Performance was evaluated using the leave-one-out cross-validation method. Comparison of predictors demonstrated the highest performance of the urine metabolome predictive model (area under the receiver-operating characteristics curve [AUC] = 0.88, 95% confidence interval [CI] [0.76, 0.99]) followed by the proteome model (AUC = 0.87, 95% CI [0.75, 0.99]) and cfRNA models (AUC = 0.68, 95% CI [0.49, 0.87]) outperforming models from other omics (Figure 2A). Top identified proteins and genes are shown in Figure S7.

The heatmap of rank values of features selected by EN from all omics is shown in Figure 2B. Hierarchical clustering was used to separate preeclamptic from normotensive pregnancies.

We next focused on our top-performing model, which was trained from the urine metabolome dataset and consisted of nine metabolites, and evaluated its performance in the validation cohort. The model validated maintaining high performance with an AUC of 0.83 (95% CI [0.62, 1.0]) (Figure 3A), confirming identified metabolites (Figure 3B) as biomarkers of preeclampsia. Furthermore, p values obtained using a separate univariate analysis demonstrated the statistical significance of each of the identified metabolites (Figure 3B). The metabolites identified by EN as the biomarkers were dihydroxybenzoic acid, tetradecadiencarnitine, adenine, dihydroxyphenylglycol *O*-sulfate, methoxyhydroxyphenylethyleneglycol, and four uncharacterized molecules ($C_{23}H_{39}NO_{19}$, $C_{26}H_{32}F_2O_7$, $C_{15}H_{25}N_3O_8S$, $C_{12}H_{27}Sn$) (Figure 3B).

Machine-learning modeling of preeclampsia over gestation

We next analyzed longitudinal data that included all samples taken during pregnancy (Figure 1) in order to capture the changes that occur due to preeclampsia during pregnancy and possibly gain insights into the development of preeclampsia. As in the early pregnancy analysis, multivariate models of preeclampsia were trained for each omics using EN (see experimental procedures). To investigate whether a joint analysis of different omics can offer further gains, predictions from separate

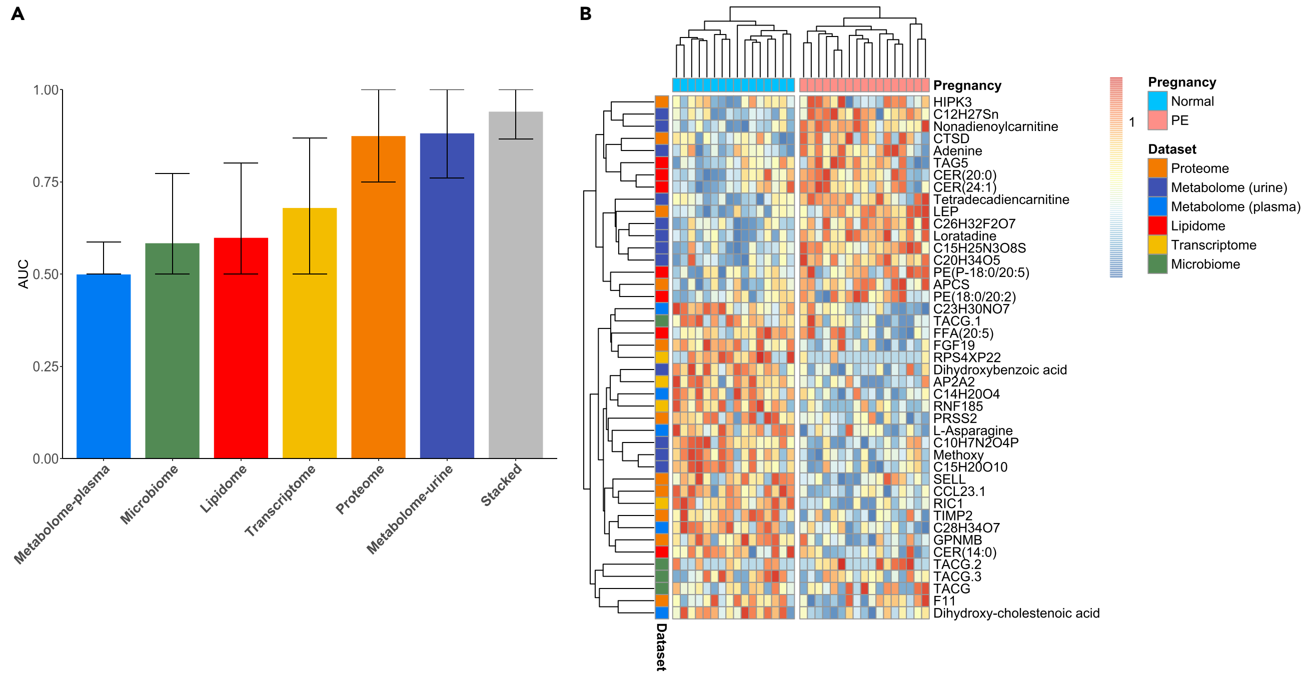

Figure 2. Prediction models in early pregnancy

Samples obtained in the first 16 weeks of pregnancy were used.

(A) Performance comparison of EN models derived from different omics in terms of the AUC. The integrated (stacked) model utilizing stacked regression exhibited the highest accuracy (AUC = 0.94, 95% CI [0.86, 1]). Among omics sets the urine metabolomic model (AUC = 0.88, 95% CI [0.76, 0.99]) and plasma proteome (AUC = 0.87, 95% CI of [0.75, 0.99]) performed best.

(B) Heatmap of ranked values of features identified by EN, perfectly distinguishing preeclamptic from normotensive women.

omics models were integrated in a joint model using stacked regression (see experimental procedures). The performance of all models was evaluated using the leave-one-out cross-validation method. The integrated model exhibited the highest prediction accuracy (AUC = 0.91, 95% CI [0.85, 0.97]), outperforming predictions from each separate model in terms of the point estimate (Figure 4A). EN models from the proteome and urine metabolome exhibited high performance (AUC = 0.89, 95% CI [0.83, 0.95]; AUC = 0.87, 95% CI [0.80, 0.94], respectively) outperforming other omics data, the same trend we observed in the early pregnancy performance. As before, the urine metabolite model was validated in an independent cohort, with an AUC of 0.87 (95% CI [0.76, 0.99]) (Figure 4B), confirming identified metabolites as true biomarkers of preeclampsia.

Top urine metabolites included adenine, isovalerylglutamic acid, uric acid ribonucleoside, 1,5-anhydroglucitol, dehydroepiandrosterone, sialyllactose, N^{ε}-acetyl-L-lysine, and nonanoylcarnitine. p values obtained using a separate univariate analysis show statistical significance of each of the identified metabolites (Figure 4C). One of the identified metabolites, uric acid ribonucleoside, is an end product in the same pathway as uric acid, whose increased concentration is typical of preeclampsia.[14] As an end product, uric acid ribonucleoside is more likely to be a sensitive biomarker. Interestingly, the uric acid levels in our data did not discriminate between controls and preeclamptic patients.

A model using top-scoring plasma proteins achieved an AUC of 0.83 (95% CI [0.73, 0.92]) (Figure 4A). The most predictive plasma proteins selected by EN included leptin (LEP), vascular endothelial growth factor A (VEGFA), L-selectin (SELL), E-selectin (SELE), interleukin-24 (IL-24), IL-22, tyrosine-protein kinase transmembrane receptor (ROR1), C-X-C motif chemokine ligand 10 (CXCL10), and SPARC-like 1 (SPARCL1) (Figure 4D), thereby confirming some of the established or indicated proteins associated with preeclampsia[15–17] (see Table S4 and discussion for further details), as well as establishing new associations. Also in this case, p values obtained using univariate analysis show that all proteins chosen by EN are statistically significant (Figure 4D).

We point out that, as expected, EN models varied slightly owing to variability of the chosen training set in each leave-one-out cross-validation step[18] and therefore, the features chosen by EN varied slightly across cross-validations. We recorded the frequency of occurrence for every feature across all cross-validation steps (shown for the proteome model in Figure S2). Having high frequency of occurrence indicates that the feature is relevant for all or a majority of samples, i.e., it is more stable.[18]

Because both in discovery and validation cohorts the prevalence of known preeclampsia risks including chronic hypertension, high BMI, and twin pregnancies was higher among preeclamptic compared with normotensive women (Table S1), we next investigated whether our multiomics model captures mostly these differences. We calculated Spearman correlation between prediction model scores and clinical variables (Figure S3). The first-trimester blood pressure was also included in the analysis. The highest correlation between the model and

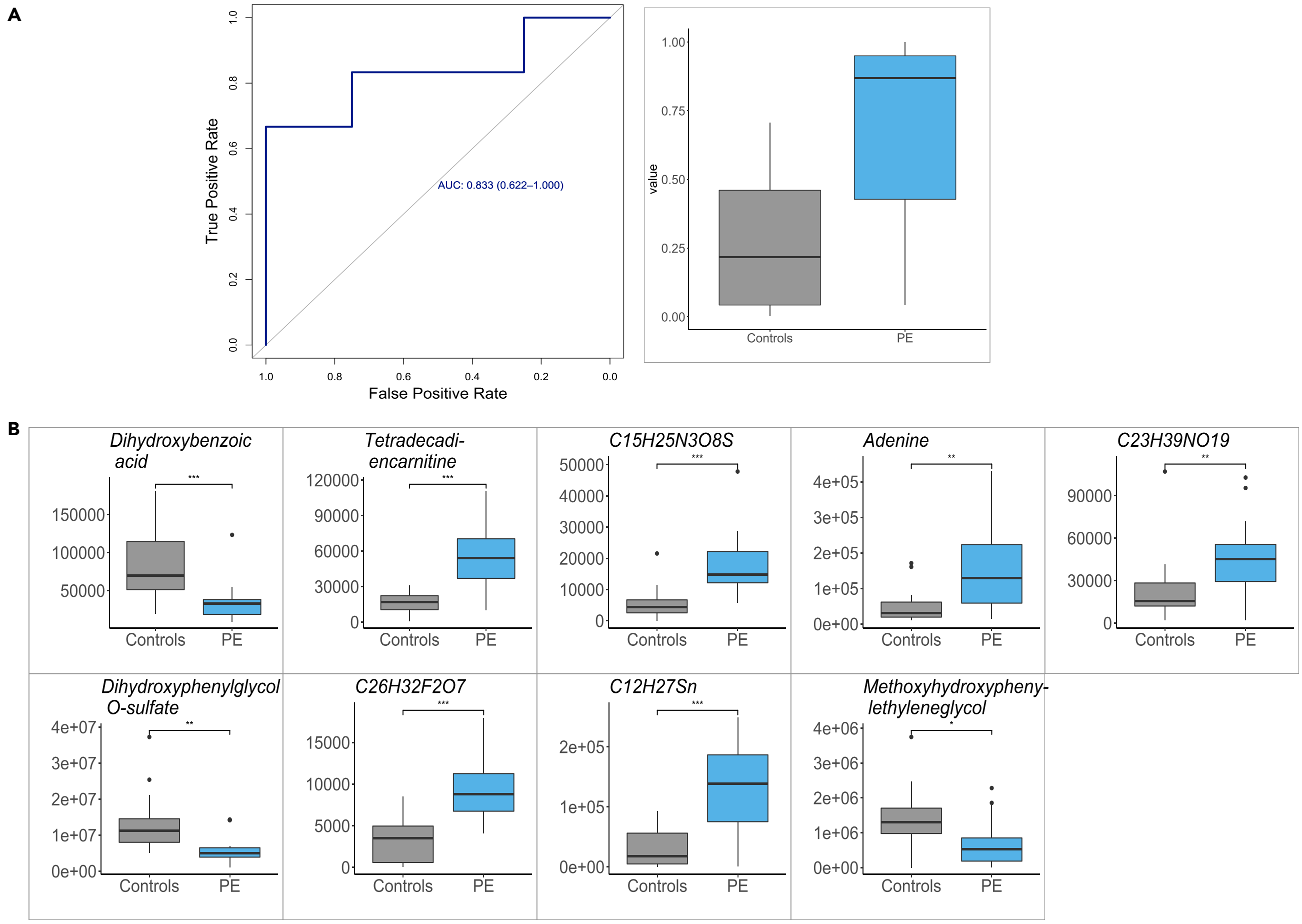

Figure 3. Urine metabolome prediction model using nine metabolites sampled early in gestation validates on the validation cohort

Samples obtained in the first 16 weeks of pregnancy are used.

(A) AUC = 0.83, 95% CI [0.62, 1] and prediction values (scores) obtained by EN for preeclamptic (PE) and normotensive women.

(B) Metabolites identified by EN as biomarkers of preeclampsia. y axis shows the value in early pregnancy stratified by normotensive (gray) and preeclamptic (light blue) pregnancies. p values obtained using Wilcoxon signed-rank univariate analysis show statistical significance of each protein (*$p \leq 0.05$, **$p \leq 0.01$, ***$p \leq 0.001$).

clinical variables, and the only one that was statistically significant, was found to be between BMI and the model ($p < 0.0086$). However, even in this case the BMI did not fully correlate with the model (Spearman correlation = 0.63) confirming that our model does not just capture differences in BMI to distinguish between preeclamptic and normotensive women. We observed a low correlation between the model (row labeled "prediction" in Figure S3) with other clinical variables, indicating that the omics model is not just describing differences in the available clinical/demographic characteristics of women but is capturing biological differences.

The correlation network of all features chosen by EN models for each of the omics sets was plotted using t-distributed stochastic neighbor embedding (t-SNE), revealing multiomics interaction of analytes associated with preeclampsia (Figure 5). Edges between features indicate a Spearman correlation >0.55. As expected, features from one omics set tend to group together, with higher correlation among them. In addition, we observe that correlation exists among different omics datasets. Exploiting these correlations may be a plausible explanation as to why the integrated prediction model outperforms individual models as shown in Figure 3. Pathway enrichment analysis revealed that three protein clusters observed in the plot were associated with different pathways: (1) pathways related to immune response (bottom cluster); (2) pathways related to neurodevelopment (middle cluster); and; (3) pathways related to intracellular signal transduction (top cluster) (Figure 5). Three metabolic pathways were enriched: (1) steroid biosynthesis; (2) tryptophan metabolism, as in the case of univariate analysis (Figure 7); and (3) β-oxidation of very long chain fatty acids whose role in preeclampsia has been previously observed.

Finally, we compared the most predictive features as identified by EN in early pregnancy versus during gestation in terms of their significance ($-\log_{10}$(p values)). Plasma proteins are shown in Figure S5A and urine metabolites in Figure S5B. We observe that a large number of proteins identified by EN stay statistically significant in both cases, including LEP, SELL, CCL23, ROR1, IL1RAP, SELL, SELE, VEGFA, IGFBP1, and SPARCL1

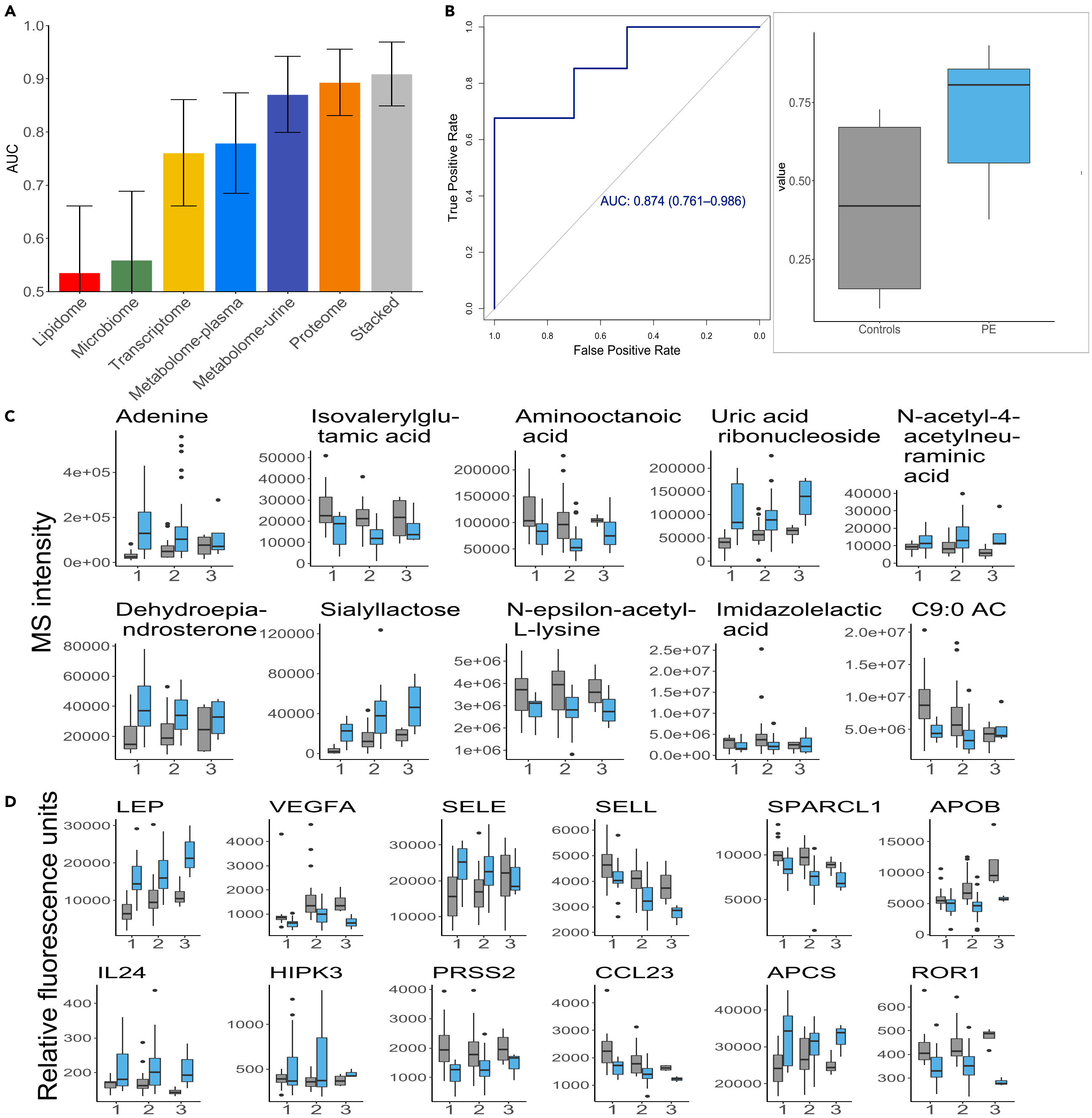

Figure 4. An integrated multiomics machine model outperforms single omics models for preeclampsia

(A) Cross-validated performance of machine-learning models in terms of the AUC is shown on the y axis. Each model was obtained using all available samples over gestation. The integrated (stacked) model utilizing stacked regression exhibited the highest accuracy (AUC = 0.91, 95% CI [0.85, 0.97]). Both proteome and metabolome (urine) had high prediction performance (AUC = 0.89, 95% CI [0.83, 0.95] proteome; AUC = 0.87, 95% CI [0.80, 0.94] urine metabolome).

(B) Urine metabolome prediction model using ten metabolites sampled over gestation validates on the validation cohort. AUC = 0.874, 95% CI [0.76, 0.99], and prediction values (scores) obtained by EN for normotensive and preeclamptic (PE) women.

(C) Metabolites identified by EN as biomarkers of preeclampsia over gestation. y axis shows values stratified by normotensive (gray) and preeclamptic (light blue) pregnancies. p values obtained using linear mixed-effects univariate analysis show statistical significance of each metabolite ($p < 0.05$).

(D) Proteins identified by EN as biomarkers of preeclampsia over gestation. y axis shows a protein value stratified by normotensive (gray) and preeclamptic (light blue) pregnancies. p values obtained using linear mixed-effects univariate analysis show statistical significance of each metabolite ($p < 0.05$).

CellPress
OPEN ACCESS

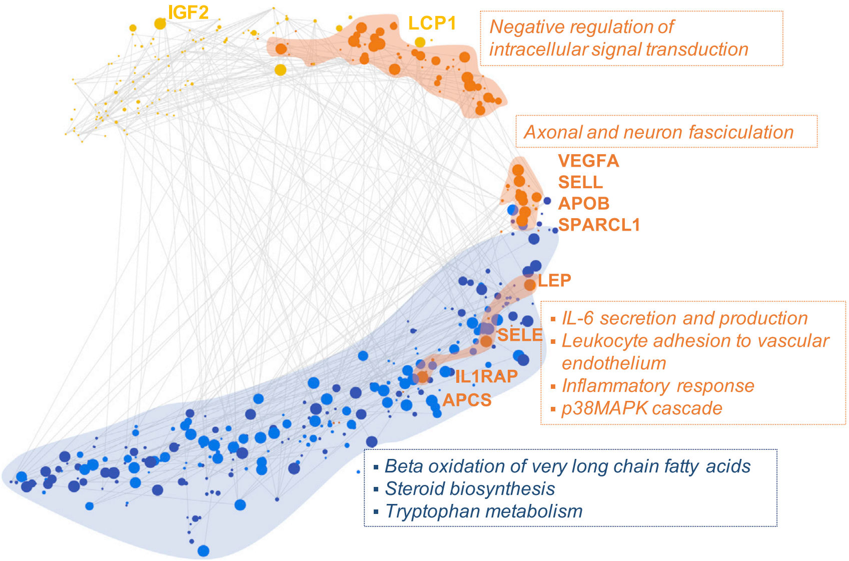

Figure 5. Visualization of predictive features of the transcriptome (yellow), proteome (orange), urine metabolome (dark blue), and plasma metabolome (light blue)

Features obtained using all available samples over gestation. Vertices represent features selected by EN laid out using t-SNE. Edges are drawn between features with Spearman correlation >0.55 clearly illustrating high correlations between different omics sets. Size of each node is proportional to the frequency at which it was chosen in prediction models during cross-validation. High frequency of occurrence indicates that a feature is relevant for all or majority of patients, resulting in a more stable model.

(Figure S4A). Some of the proteins are significant over gestation but not early in pregnancy (e.g., APOB) possibly due to a smaller number of samples. A similar trend is observed for urine metabolites (Figure S4B). Also noteworthy is that because EN uses sparsity, a feature that is associated with preeclampsia may be excluded from the final model if that model already includes another feature highly correlated with the original one. This is especially true in scenarios with a large number of features and high-dimensional regime such as in our study. This effect is illustrated in Figure S5, showing the difference in chosen features for prediction models over gestation and in early pregnancy.

Single-cell characterization of the immune system

Preeclampsia is strongly associated with inflammation and aberrant maternal immune system adaptations during pregnancy.[19] To assess immunity—which is complementary to pathways covered by proteins and metabolites—and connect differential abundances of plasma proteins and urine metabolites in preeclamptic pregnancies to biological changes, immune-system-wide mass cytometry measurements of single cells obtained in a subset of the same patient cohort were integrated with our plasma proteome and urine metabolome prediction models, as these two models had the best accuracy. Immune cell dynamics between first- and second-trimester blood samples obtained from high-dimensional mass cytometry were previously used to develop a prediction model of preeclampsia.[20] We found that seven (out of eight) of the immune features reported by Han et al.[20] correlated highly with the prediction based on our integrated algorithm (Spearman correlation $p < 0.05$) (Figure 6A, highlighted in orange), confirming the predictive value of immune cell features as well as plasma proteins and urine metabolites. To investigate whether this correlation between predictive features was biologically meaningful, we focused on the correlations of feature behavior between the eight earlier reported predictive immune features (Figure 6A) and the top 12 most informative plasma proteome features (Figure 4D) across pregnancy (Figure 6B). LEP and SELL levels were particularly strongly correlated with the eight immune cell features (Figure 6B). Interestingly, basal pSTAT5 signaling in T helper 1 (Th1) cells ($CD4^+Tbet^+$), the top immune feature to distinguish control from preeclamptic pregnancies,[20] correlated with LEP levels in both control and preeclamptic patients. Uniquely in preeclamptic cases, LEP levels were correlated with basal pSTAT1 signaling in intermediate myeloid cells (intMCs) (Spearman correlation $p = 0.002$) and basal STAT5 signaling in myeloid dendritic cells (mDCs) (Spearman correlation $p = 0.01$). Moreover, SELL levels were uniquely correlated with immune features in preeclamptic pregnancies and not with controls, i.e., correlated with basal pNFkB and pSTAT1 signaling in cMCs, basal pSTAT5 signaling in Th1 cells and mDCs, and basal pMAPKAPK2 signaling in naive CD4 T cells. Preeclamptic pregnancies were not characterized by—in other words, had potentially lost—concerted proteome/immune behavior, which was prominently observed in healthy pregnancies, i.e., correlations of leptin with basal pP38 signaling in T regulatory (Treg) and T cell receptor $\gamma\delta$ (TCR$\gamma\delta$) cells. These correlations exemplify the biological connection between responsiveness of immune cells and its plasma environment.

Relationship between clinical data and omics measurements

Clinical and demographics data contain maternal characteristics known to be associated with the risk of preeclampsia, e.g., preexisting hypertension, race, BMI, height, and gravida. We combined ten variables that were available in this dataset (Table S1) with the most predictive sets, (1) plasma proteome and (2) urine metabolome models, to better understand their mutual relationship. The ten clinical variables were included together with the top ten omics features, all combined in the single cross-validation step. Inclusion of clinical and demographics data improved the performance when combined with both the plasma proteome and the urine metabolome (urine metabolome AUC = 0.96, 95% CI [0.92, 0.99]; proteome AUC = 0.91, 95% CI [0.85, 0.97]) (Figure S6A). The most predictive clinical variables included maternal age, BMI, height, and preexisting hypertension. We observed several significant correlations (Spearman correlation $p < 0.05$) between clinical variables and plasma proteins/urine metabolites that were present only among preeclamptic women. These included: leptin with maternal BMI/weight, in agreement with existing literature;[21] CCL23 with

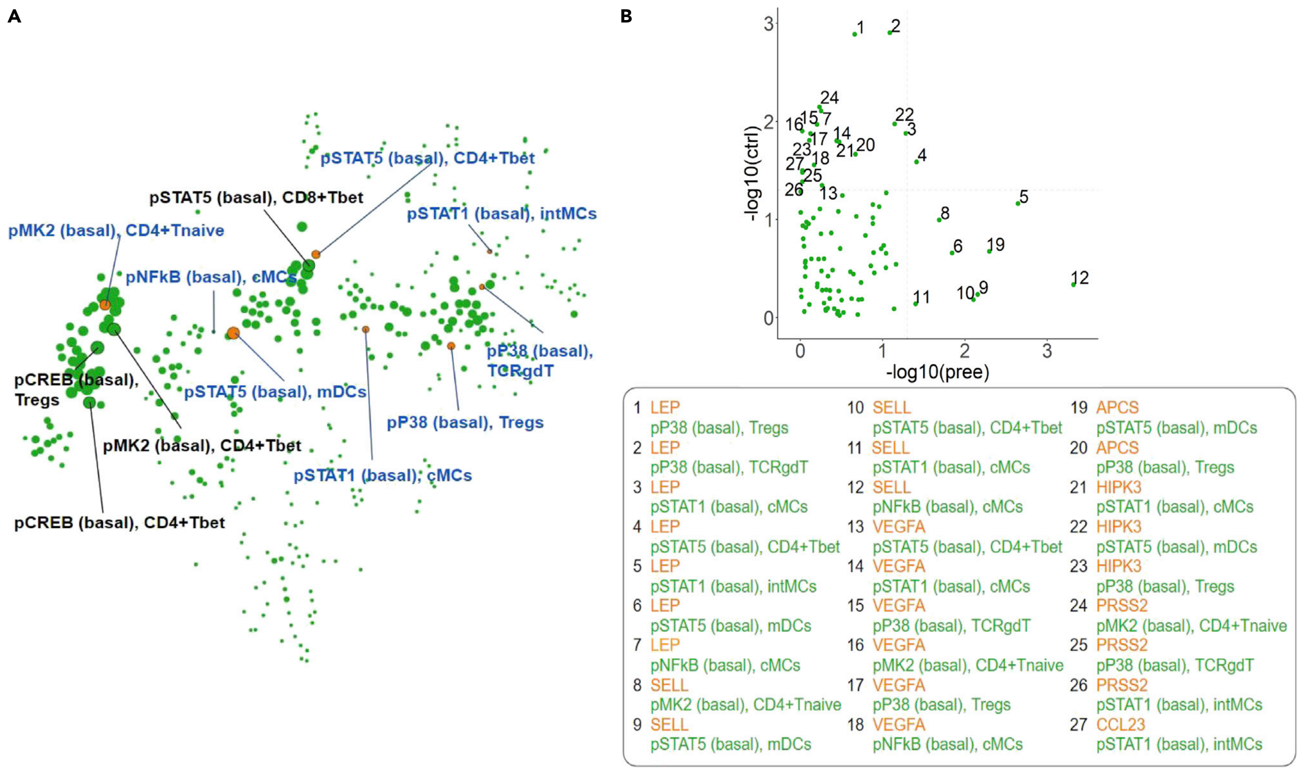

Figure 6. Correlation between predictive immune features and multiomics model

(A) Previously identified predictive immune features strongly correlate with the multiomics predictive model. Visualization shows features most correlated with the prediction of the stacked model. Features shown in orange are the seven most predictive immunome features that also highly correlate with the multiomics predictive model. Size of each node is proportional to the $-\log_{10}$(p value) of Spearman correlation.

(B) Comparison of p value of correlation for the top immune and top proteome features. Each node is a pair comprising an immune and a proteome feature.

height; SELL with gravida (Figure S6B); maternal age with adenine—previously observed[22]—and maternal age with isovalerylglutamic acid (Figure S6C).

In addition, to compare the above performance with the performance of a baseline model, we trained EN only on the available maternal and pregnancy characteristics. The obtained model still had a good accuracy, AUC = 0.85, 95% CI [0.66, 1.0], but the performance, as expected, was lower compared with the combined model. The most predictive variables in the model were maternal hypertension, BMI, race, age, and number of babies in the current pregnancy. This is in line with results in our previous work that developed a machine-learning model for early prediction of preeclampsia from electronic health record data.[23]

Univariate analysis of preeclampsia pathogenesis from multiomics measurements

We next present univariate analysis with the Benjamini-Hochberg procedure that identifies all features significantly associated with preeclampsia. We further used these features to perform pathway enrichment analysis.

Over the course of pregnancy

Changes over gestation of 1,215 metabolic features among 8,718 were significantly associated with preeclampsia outcome (false discovery rate [FDR] < 0.05, linear mixed-effects [LME] model with Benjamini-Hochberg procedure). Pathway enrichment analysis using these urine metabolites identified the following pathways ($p < 0.05$) (Figure 7A): (1) tryptophan metabolism; (2) caffeine metabolism; (3) tyrosine metabolism; (4) steroid hormone biosynthesis; (5) pentose and glucuronate interconversions; (6) linoleic acid metabolism. The steroid hormone biosynthesis pathway plays an important role in pregnancy progression.[24] Both the steroid hormone biosynthesis pathway and the caffeine metabolism pathways and caffeine metabolites have previously been associated with pregnancy,[25,26] and tryptophan metabolism with preeclampsia.[27] Metabolites in the steroid hormone biosynthesis pathway and in the caffeine metabolism pathway present in the data with high level of significance are respectively shown in Figures S8C and S8D. The comparison between enrichment factors and statistical significance of pathways enriched over gestation versus in early pregnancy is shown in Figure S22.

Among 1,305 proteins, 437 had changes that were significantly associated with preeclampsia outcome over gestation (FDR < 0.05, LME model with Benjamini-Hochberg procedure). The top 64 proteins at significance level $p < 5\times10^{-4}$ (LME model) showed markedly different values between normotensive and preeclamptic women (Figure S9). Top proteins included IL-1 receptor accessory protein (IL1RAP) and SELL, both known to play a role in the immune response.[28] Enriched pathways grouped into ten biological processes, the most prevalent being positive regulation of cellular processes (including biological, cellular,

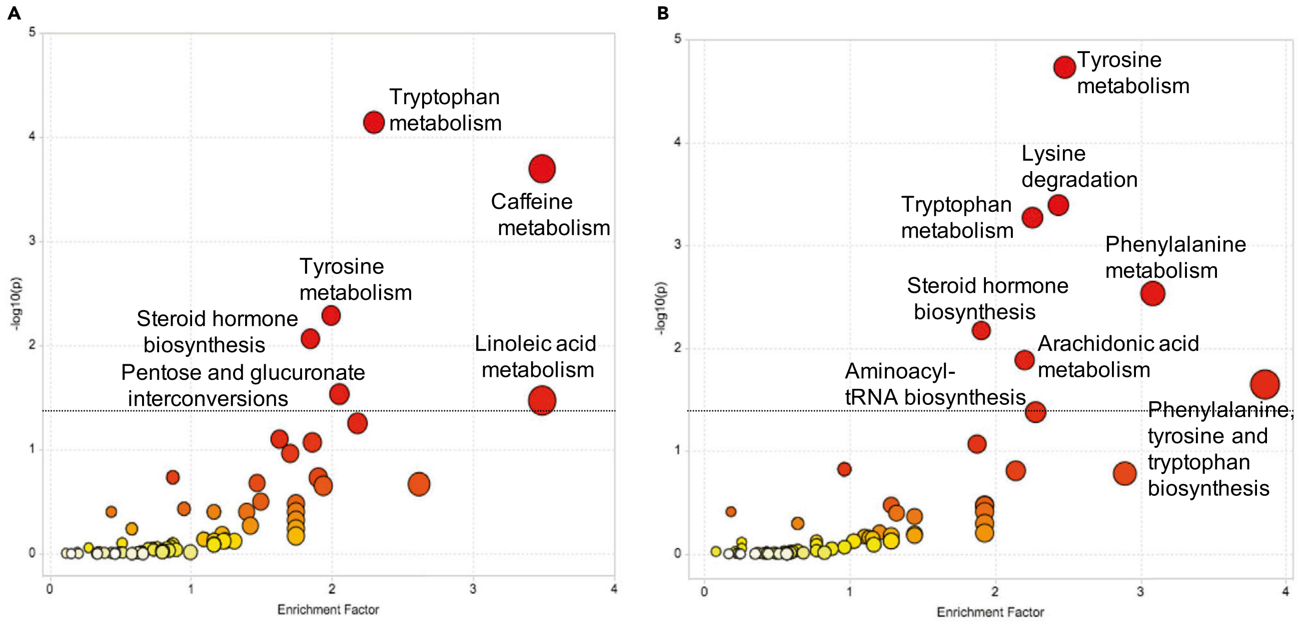

Figure 7. Identified enriched pathways from urine metabolome urine over gestation and in early pregnancy

(A) Pathway enrichment analysis over gestation using metabolites from urine that were significant (FDR < 0.05, Wilcoxon signed-rank test with Benjamini-Hochberg procedure). Pathways shown above the dotted line were significant ($p < 0.05$).

(B) Pathway enrichment analysis for early pregnancy using metabolites from urine that were significant (FDR < 0.05, linear mixed-effects model with Benjamini-Hochberg procedure). The color and the size of a circle are proportional to the $-\log(p)$ and pathway impact value, respectively, where p denotes a p value.

protein metabolic, immune system, and apoptotic processes among others) (46.4%) (Figure S10). In the cfRNA set, 306 features were significantly associated with preeclampsia outcome over gestation (FDR < 0.05, LME model with Benjamini-Hochberg procedure). Enriched pathways grouped into 11 biological processes, the most prevalent being RNA splicing (37.3%) (Figure S11). Top features included YOD1 (known to be related to developmental processes[29]), BIRC2, CEP63, and LCP1 (also previously implicated with preeclampsia[30]). A network of top proteome, transcriptome, and urine and plasma metabolome features is shown in Figure S12.

Early pregnancy

In early pregnancy, 497 out of 8,718 urine metabolic features had changes significantly associated with preeclampsia when compared with normotensive controls (FDR < 0.05, Wilcoxon signed-rank test with Benjamini-Hochberg procedure). Pathway enrichment analysis on these urine metabolites identified the following pathways ($p < 0.05$) (Figure 7B): (1) tyrosine metabolism; (2) lysine degradation; (3) tryptophan metabolism; (4) phenylalanine metabolism; (5) steroid hormone biosynthesis; (6) arachidonic acid metabolism; (7) phenylalanine, tyrosine, and tryptophan biosynthesis; (8) aminoacyl-tRNA biosynthesis. Arachidonic acid metabolism is a central regulator of the inflammatory response and has a known role in the pathogenesis of preeclampsia.[31] Similarly, tryptophan metabolism has an important role in pregnancy, providing increased protein synthesis by the mother, fetal growth development; and serotonin for signaling pathways.[32] Individual metabolites from these two pathways are shown in Figures S8A and S8B.

In the proteome set containing 1,305 proteins, three proteins—LEP, CCL23, and FAM3D—were significantly associated with preeclampsia outcome (FDR < 0.05, Wilcoxon signed-rank test with Benjamini-Hochberg procedure) identifying one significantly enriched pathway, negative regulation of glucagon secretion (Fisher's exact test with Benjamini-Hochberg procedure, FDR < 0.05). The reason we did not adjust for covariates, specifically BMI—the only covariate with statistically significant correlation with the model predictions (Figure S3)—is that we wanted to capture the underlying biology including the mechanism under which the existing factors such as BMI are associated with preeclampsia. By adjusting for BMI, we would potentially remove pathways otherwise enriched and involved in preeclampsia.

Outlier analysis

We observed that a few women in our cohort were consistently misclassified by our prediction algorithm (Figure S13). A few normotensive control women resembled those with preeclampsia on a molecular level in some of the top predictive features across omics sets. Vice versa, there were some preeclamptic women whose top molecular features more closely resembled those of controls. Reexamination of the clinical charts revealed that one of the preeclamptic women, while clearly hypertensive, had proteinuria in the context of gross hematuria, obscuring whether proteinuria was related to preeclampsia. Therefore, she may have been misdiagnosed with preeclampsia but rather only had gestational hypertension. This highlights that the predictive model can pick up discrepancies within the clinical chart. For the other women whose clinical diagnosis held, this implies that their phenotypic features that classified them in either normotensive or preeclampsia group did not match their molecular phenotypes. Of interest, one preeclamptic woman, which the prediction classified as control, developed HELLP syndrome very late in gestation at 41 + 3 weeks. Therefore, if she had

delivered closer to the due date she would have been considered a control. Thus, if others in the control group have a similar molecular phenotype, this may represent a late-onset preeclampsia related to placental aging in the post-term period.

DISCUSSION

Recent omics studies of preeclampsia typically included up to two omics datasets.[10,33,34] Our study presents an integrated analysis of six high-throughput omics datasets obtained on the same biological sample, containing more than 50,000 measurements per sample. This multiomics analysis enabled uniform comparison of omics sets and revealed improved predictive ability for preeclampsia status relative to individual biological modalities, and indications of biological processes associated with the disease across multiple modalities. The first part of the analysis focused on early prediction of preeclampsia with the goal of comparing the six omics and identifying the best biomarkers. We then used a multiomics approach to integrate six omics datasets into one integrated (stacked) prediction model. The multiomics analysis demonstrated that the plasma protein and urine metabolome had the highest accuracy, both early in pregnancy (Figure 2A) and over gestation (Figure 4A). For that reason, we followed this with a more targeted analysis of plasma proteins and urine metabolites that were identified as having the highest accuracy. Ultimately, our goal is to develop a simple diagnostic test with high accuracy, and these two omics datasets were identified as most promising from our analysis.

One of the main strengths of our study is that, in our cohort, biological samples were not only collected longitudinally from each woman, but each individual sample was also simultaneously measured for proteome, transcriptome, metabolome, lipidome, and vaginal swab for microbiome, thereby providing a unique opportunity to systematically study changes attributable to preeclampsia over gestation, and compare the capability of each of these omics sets to predict and characterize preeclampsia. All 50,000 measurements were used in the prediction algorithm to agnostically identify the best biomarkers of preeclampsia.

Among our six omics, urine metabolomic and plasma proteomic datasets demonstrated the highest prediction accuracies, both over gestation and early in pregnancy. A prediction model using a small number of urine metabolites provided high accuracy over gestation (AUC = 0.88, cross-validated) and early in pregnancy (AUC = 0.875, cross-validated). The prediction model was validated on an independent cohort (AUC = 0.83 in early pregnancy; AUC = 0.87 over gestation), confirming identified metabolites as true biomarkers. Univariate analysis demonstrated the statistical significance of these biomarkers.

The EN prediction model with plasma proteins achieved AUC of 0.83 over gestation and of 0.88 in early pregnancy. Several of the proteins identified by our model as the most predictive have previously been well established as biomarkers of preeclampsia (VEGFA,[15] LEP,[16] SELL,[35] CXCL10,[36] ROR1,[37] and IL1RAP[38]), further validating our results. Some of the identified proteins have been previously indicated in individual studies but have not yet been confirmed (IL-24,[17] HIPK3,[39] and SPARCL1[40]), and some have been identified for the first time (IL-22). While these biomarkers were previously examined in typically more targeted studies examining a single or small group of these proteins (e.g., IL-24[17]), our agnostic approach demonstrates that put together, their combined measurements result in an accurate model of preeclampsia. One of identified proteins in our model was VEGFA. Reduced levels of VEGFA have previously been described in preeclamptic pregnancies, owing to increased levels of placental soluble FMS-like tyrosine kinase-1 (sFLT-1), which validate our study.[15,41,42] Among the other known biomarkers of preeclampsia—sFLT-1, pregnancy-associated plasma protein A (PAPP-A), placental growth factor (PIGF), and endoglin (ENG)—PIGF and PAPP-A were indeed significantly different between normotensive and preeclamptic women (Figure S15). The fact that ENG and sFLT-1 were not significant may be in part due to the small size of our cohort. In addition, we point out that sFLT-1 is a good predictor of preeclampsia later in pregnancy and once suggestive clinical features are observed,[43] whereas the majority of our samples were taken before that point. Clinically, it is the sFLT-1/PIGF ratio that is used as a biomarker and not individual levels (Figure S14B). Throughout pregnancy PIGF levels are increasing until sFLT-1 levels start to increase, which is consistent with what is observed in our data (Figure S14) and suggests a variety of new hypotheses for testing. We did not include PP13 (galectin13) measurements, another known biomarker of preeclampsia.

Preeclampsia is accompanied by a dysregulated maternal immune adaptation to pregnancy, which is already detectable in early pregnancy.[19,20] This aberrant signature was previously identified in women who developed preeclampsia later.[20] Here we report that the intricate functional capacities of immune cells are coevolving with their environment throughout the course of pregnancy, showing that top informative immune feature levels are highly correlated with top informative plasma proteins. This interconnectedness supports both prediction approaches, confirming their individual usefulness while complementing the validity of each approach. The results highlight the known pathophysiology of preeclampsia and suggest novel associations between immunological and proteomic dynamics. In preeclamptic pregnancies, immune responses were uniquely correlated with levels of LEP and SELL.

LEP, known to be elevated in the plasma of preeclamptic women,[16] is an immune regulatory hormone produced by adipose tissue and the placenta.[16,44] LEP activates the JAK/STAT and MAPK pathways directly through binding to the leptin receptor expressed on leukocytes, and thereby modulates both innate and adaptive immune responses,[45,46] including skewing of CD4 T cells toward Th1 polarization[47] and inhibiting Treg proliferation.[46] Accordingly, we observed that LEP levels in preeclamptic and control pregnancies correlated with STAT and MAPK pathway signaling both in innate and adaptive immune cells, suggesting that dysregulated leptin levels in preeclamptic pregnancies might contribute to the aberrant immune signature while, reciprocally, inflammation itself might enhance plasma leptin levels.[44,45] Moreover, while in healthy pregnancies LEP levels correlated with p38 signaling in Treg and TCR$\gamma\delta$, this correlation was lost in preeclamptic pregnancies, suggesting that regulation of immune tolerance might be disrupted in preeclamptic pregnancies.

Furthermore, we reported decreased SELL levels in preeclamptic pregnancies that correlated with basal pSTAT,

pNFkB, and pMAPKAP2 signaling in innate (mDC and cMC) and adaptive immune cells (Th1 and naive CD4 T cells). SELL is shed from leukocytes during activation and migration, and soluble L-selectin can be used as a surrogate marker for inflammation.[48] Notably, a drop in soluble SELL levels is observed during sepsis.[49] Previous studies reported conflicting results for circulating soluble SELL levels in preeclampsia,[35,50,51] including low soluble SELL levels at 20 weeks of gestation, prior to onset of preeclampsia.[50] Preeclampsia-associated enhanced ectodomain shedding of cell-adhesion molecules could be directly linked to changes in signaling responses in circulating immune cells by shedding-mediated activation of intracellular pathways.[48] Alternatively, the correlation could reflect independent inflammatory mechanisms, as decreased levels of circulating SELL have been proposed to be due to its adsorption to luminal vascular ligands, which are upregulated by an activated endothelium, a feature of preeclampsia.[6,50,52]

The model from urine metabolites predicted preeclampsia with highest accuracy. Enrichment analysis identified discriminant biological pathways associated with preeclampsia when considering early and all time points. The steroid hormone biosynthesis pathway was significant ($p < 0.05$) in both models while arachidonic acid metabolism was significant in early pregnancy. Arachidonic acid is a precursor to a myriad of bioactive lipids including prostaglandins (PGs), prostacyclin, thromboxane, hydroperoxyeicosatetraenoic acid, leukotrienes, lipoxins, hypoxins, anandamide, and epoxyeicosatrienoic acids, which play key roles in inflammatory, vascular, and coagulation processes.[53] As early as the 1960s the role of the eicosanoids in preeclampsia pathogenesis was proposed, and by the 1970s evidence supported that an increase in thromboxane (TXA2; produced by platelets) over prostacyclin (PGI2; produced by endothelium) associated with preeclampsia.[54] This is one of the biological underpinnings for the use of low-dose aspirin for the prevention of preeclampsia. Mills et al.[55] reported longitudinal measurements of the urinary metabolites of thromboxane and PGI2 throughout gestation. Although they did not find a significant increase in the urinary concentrations of TXA2, they did find a significant decrease in PGI2 as early as 13–16 weeks of gestation and a significant elevation in the ratio of thromboxane to PGI2 as early as 17–20 weeks of gestation in women destined to develop preeclampsia. While this PG imbalance is noted both prior to and at the time of clinical presentation (after 20 weeks), the fact that arachidonic acid metabolism was only observed in early pregnancy may explain why clinical studies note that low-dose aspirin initiation prior to 16 weeks is needed for significant prevention of preeclampsia.[56]

The tryptophan pathway was identified as highly associated with preeclampsia over gestation (Figure 7). Indolea mine-2,3-dioxygenase (IDO) is the first and rate-limiting enzyme in this pathway producing kynurenine, which then is converted into a number of bioactive metabolites. IDO is an intracellular enzyme produced by many cell types and while not secreted, impacts neighboring cells by tryptophan depletion and production of bioactive metabolites. The role of IDO in both normal and abnormal pregnancies, including preeclampsia, has been recently reviewed.[57] IDO expression increases with pregnancy, and tryptophan depletion in the placenta inhibits T cell-mediated rejection of semi-allogeneic fetal tissues.[58] Kynurenine is an endogenous ligand that activates the aryl hydrocarbon receptor (AhR).[59] This activation skews the differentiation of T cells to immunosuppressive Tregs rather than proinflammatory Th17 cells after exposure to transforming growth factor β.[60,61] Notably, kynurenic acid and xanthurenic acid, two metabolites of kynurenine, can also activate AhR signaling and may participate in immune regulation.[62,63] Therefore, deficiency of IDO impacts Treg development. Notably, IDO knockout mice, when pregnant, develop a preeclampsia-like phenotype.[64] The metabolic signal related to tryptophan metabolism in the model over gestation may be related to the immune signature of preeclampsia, highlighting the importance of immune alterations occurring in the later stages of preeclampsia. Caffeine metabolism was also identified as highly associated with preeclampsia over gestation. This pathway and caffeine metabolites have previously been associated with pregnancy.[25,26]

Models to predict preeclampsia early in pregnancy were previously based on maternal characteristics (demographics and medical history), followed by addition of uterine artery Doppler measurements and specific biomarkers.[65–70] Levels of angiogenic and/or antiangiogenic proteins (PlGF, sFlt-1, and ENG), or their ratios, have been established as biomarkers with high prediction accuracy later in pregnancy.[15,41,71] More recently, analysis of omics datasets have been successfully applied to identify various biomarkers related to preeclampsia.[10,33,72] Most of these studies were based on measurements from one or at most two omics datasets, and often from samples taken only at one time point during pregnancy. Here we show that clinical and demographic characteristics (i.e., weight, height, race) were complementary to omics measurements and improved prediction models.

Another important problem would be to develop a more specific model to predict severe preeclampsia. Among existing models for prediction of preeclampsia based on maternal characteristics and specific biomarkers, there are fewer that predict specifically severe preeclampsia,[73–75] and a prediction model from multiomics assays would be an important contribution to this literature. Given the small number of women with severe preeclampsia in our cohorts, we plan to address this topic in future studies by analyzing cohorts richer in this pregnancy outcome.

Our study is limited by a small sample size—a typical limitation when high-cost multiomics analysis is conducted—and consideration of a cohort from a single hospital. Another limitation comes from the fact that targeted assays (and untargeted assays that rely on a reference database) need to be carefully validated for the samples to which they are applied. For example, our targeted aptamer-based proteomics assay has been carefully validated in human plasma samples but cannot be readily applied to vaginal swabs without careful validation studies. Inherently to the machine-learning approach, developing a prediction model depends on the underlying sample distribution of the data used. Distribution shift, caused by differences among various cohorts, can impact the performance of a machine-learning algorithm.[76] For this reason, we a took special care in obtaining our results by: (1) performing careful machine-learning analysis to avoid overfitting; (2) validating our model on an independent cohort; (3) demonstrating that features identified by machine learning are statistically significant when analyzed by a separate, univariate analysis; and (4) examining our prediction model in relation

to a previously established model from immunological data. In this study, the mass cytometry data were not included in the multiomics prediction model because these data were not available for 14 out of 33 women. However, integrative analysis of the restricted set of common samples revealed important connections between our model and key immune features.

While encouraging, our results need to be validated on a larger, more diverse set of women. If the results prove generalizable, our findings demonstrating high predictive power from a small number of urine metabolites and proteins could lead to a simple prediction test based on a small number of urine metabolites, suitable for use in both developed and developing parts of the world.

EXPERIMENTAL PROCEDURES

Resource availability

Lead contact

Further information and requests for resources should be directed to and will be fulfilled by the lead contact, Ivana Marić (ivanam@stanford.edu).

Materials availability

This study did not generate new unique reagents.

Data and code availability

Raw and processed untargeted metabolomics data were deposited to the Metabolomics Workbench with the following study IDs: ST001889 for plasma and ST001890 for urine. The Project DOI for these studies is https://doi.org/10.21228/M8WD84.

Our microbiome reads have been submitted to SRA. The BioProject accession is PRJNA752652.

https://dataview.ncbi.nlm.nih.gov/object/PRJNA752652?reviewer=aofjjbr2j556u6vckeolc1i2t2.

Transcriptome data are available at:

https://drive.google.com/file/d/12JXm30he5psipz6iCiIUtZxWsy-IwG08/view?usp=sharing.

The data that support the findings of this study have also been deposited on GitHub in the form of csv files at https://github.com/ivanam5/Multiomics_Preeclampsia. All data except for the clinical variables have been made available. Clinical variables cannot be shared due to the HIPAA constraints.

Code to reproduce main analyses in the manuscript is available on GitHub at https://github.com/ivanam5/Multiomics_Preeclampsia. R software is needed to run the code.

Study design

We performed a longitudinal, prospective study of a cohort of pregnant women receiving routine ante- and postpartum care at the Lucile Packard Children's Hospital at Stanford University, California, as previously described.[20,77] Women were eligible for the study if they were at least 18 years of age and were in their first trimester of pregnancy. The study was approved by the Institutional Review Board of Stanford University (#21956), and all participants signed an informed consent form.

Peripheral blood samples (for mass cytometry analysis), plasma samples (for proteomic, transcriptomic [cfRNA], metabolomic, and lipidomic analyses), urine samples (for metabolomic analysis), and vaginal swabs (for microbiome analysis) were collected from each woman at two or three time points during pregnancy. Sample collection, their analyses, and quality assessment for some of them was previously described,[9] and are presented in the supplemental information. The validation cohort included 16 women from the same hospital, for which longitudinal samples with only metabolomic analyses were available. Metabolomic analyses were performed following the same methodology as for the discovery cohort.

Definition of preeclampsia

Preeclampsia was defined using the American College of Obstetrics and Gynecology classification[3] as follows: hypertension that develops after 20 weeks of gestation (systolic or diastolic blood pressure of 140 and/or 90 mmHg, respectively, measured on at least two occasions, 4 h to 1 week apart) and proteinuria (300 mg in a 24-h urine collection, a protein/creatinine ratio of at least 0.3 [each measured as mg/dL] or, if these were not readily available, a random urine specimen containing 1+ protein by dipstick). In the absence of proteinuria, preeclampsia was diagnosed if the presence of thrombocytopenia (platelet count less than 100,000/μL), impaired liver function (elevated blood levels of liver transaminases to twice the normal concentration), the new development of renal insufficiency (elevated serum creatinine greater than 1.1 mg/dL), pulmonary edema, or new-onset cerebral or visual disturbances. Early-onset and late-onset preeclampsia were distinguished based on whether diagnosis was before or after 34 weeks of gestation.

Machine-learning analyses

Prediction models for each omics dataset were developed for each omics set using an EN model.[13] Given $N \times p$ matrix of predictors (measurements) $X = (x_1, \ldots x_p)$ and a vector of responses $y = (y_1, \ldots, y_N)$, regression coefficients $\beta = (\beta_1, \ldots, \beta_p)$ and an intercept term β_0 in the EN model are obtained by maximizing the likelihood, or equivalently minimizing the negative log likelihood together with L_1 and L_2 penalty:

$$\left[\frac{1}{N}\sum_{i=1}^{N} L(\beta_0, \beta; y, X) + \lambda((1-\alpha)\|\beta\|_2 + \alpha\|\beta\|)\right]. \qquad \text{(Equation 1)}$$

Logistic regression was used, for which the negative log likelihood evaluates to

$$L(\beta_0, \beta; y, X) = \sum_{i=1}^{N} y_i\left(\beta_0 + x_i^T\beta\right) - log\left(1 + e^{\beta_0 + x_i^T\beta}\right).$$

For the high-dimensional setting ($p \gg N$) considered here, EN, which performs both shrinkage and automatic selection of predictors, can provide both high accuracy and facilitate interpretability. Prior to training a model, low-variance measurements from transcriptome and microbiome were filtered out. Other omics sets did not have near-zero variance measurements.

For integration of omics datasets (Figure 4), a nested (two-level) cross-validation approach was used to train predictive models to estimate the risk of preeclampsia (Figure S15). At the first level, the EN model was used as described above (Equation 1). At the second level, predictions of EN models were integrated using stacked regression.[78–80] Specifically, to use EN models in the two-level approach, for each modality k, $k = 1, \ldots K$ and data $X^k = (x_1^k, \ldots x_{p_k}^k)$, a leave-one-out EN model, denoted $c_{-i}^k(x_i)$, was repeatedly fitted and evaluated at patient i. At the second level, stacked regression with non-negative coefficients[14] was used, so that the regression coefficients of the final model $(\gamma_1, \ldots, \gamma_K)$ were determined by

$$\min \sum_{i=1}^{N}\left(y_i - \sum_{k=1}^{K}\gamma_k c_{-i}^k\left(x_i^k\right)\right)^2 s.t.\ \gamma_i \geq 0.$$

Note that the leave-one-out approach used in stacked regression has a purpose to form an unbiased linear combination of EN models.[79] In contrast to the original stacking approach in which different prediction models fit on the same data are stacked, here, we use the same model (EN) but fit to different omics to obtain different estimators which are then stacked. A stacked regression model can be regarded as a special case of a two-layer neural network; its special construction provides for an easier interpretation.

We point out that the nested cross-validation is done where in each step of cross-validation, EN models for each omics set are first trained and then the stacked model is trained in the same step. After the stacked model is built, it is tested on the test patient who was left out in the outer cross-validation loop. Therefore, no leakage of information between training and test data occurred (see detailed flowchart in Figure S15). In addition, the manuscript is accompanied by data and source code to enable both independent reproduction of our results and evaluation of the machine-learning techniques used. Furthermore, tuning of two parameters of EN algorithm, λ,α shown in Equation 1 above was also performed without using the test set data: function cv.glmnet in R package glmnet was used for implementation on EN that internally performs a separate cross-validation using the training set to choose λ. The value of α was not optimized and it was set to α = 0.9.

One of our main goals was to identify a small subset of biomarkers that can predict preeclampsia with high accuracy and could thereby be used as a simple diagnostic test. For these reasons, performance of the refitted EN model for each omics set was next evaluated by treating the EN model as a model-selection procedure and performing a refitting step on the selected support (features) in the same cross-validation step.[81] The refitted model is then tested on the test patient who was left out in the cross-validation loop (see detailed flowchart in Figure S16). It is known that L_1-penalization used in EN performs excessive shrinkage of the large coefficients of the prediction model.[82] Refitting can resolve this problem and obtain a model with a smaller number of features.

Finally, to investigate a possible gain from integration of available clinical and demographic characteristics, a prediction model that takes omics (from a specific multiomics set) and clinical and demographics variables as an input to an EN model was fit and evaluated.

To build the model over gestation, multiple (2–3) samples available from the same patient were treated as independent inputs to the algorithm. Once prediction scores were obtained for each sample, scores for a same patient were averaged into the final risk score for that patient. Performance was estimated using a leave-one-out cross-validation procedure, such that in each cross-validation step all measurements of one patient are left out from the training set and are used for testing. In addition, urine metabolome prediction models, with and without clinical/demographics variables, were validated on a separate validation cohort. This dataset was produced independently of the initial dataset and was only used once at the end. Specifically, a prediction model was trained and its parameters determined using the discovery cohort and was then tested only once on the validation cohort. The prediction accuracy of the model in terms of the area under receiver-operating characteristics curve was evaluated. t-SNE[83] was used for network visualization in Figure 5. For network visualization in Figure S13, a k-nearest-neighbor graph (with $k = 2$) was constructed between features. The network layout was computed with the LargeVis algorithm.[84] The analysis was performed using R software (version 3.6.1).

Pathway enrichment analysis

Univariate analysis was performed to identify features with significant associations between each feature and the pregnancy outcome, both in early pregnancy (Wilcoxon signed-rank test) and over gestation (LME model). The Benjamini-Hochberg procedure was used to control the FDR.[85] Metabolome pathway enrichment analysis on identified metabolites was performed using MetaboAnalyst.[86] The hypergeometric test was used for over-representation analysis in MetaboAnalyst. Proteome pathway enrichment analysis was performed using GeneOntology[87,88] and topology-based Gene Ontology scoring (topGo), an R software package. Circular Gene Ontology (CirGO) software for visualizing two-level hierarchically structured gene ontology terms[89] was used to visualize proteome and transcriptome pathway enrichment.

SUPPLEMENTAL INFORMATION

Supplemental information can be found online at https://doi.org/10.1016/j.patter.2022.100655.

ACKNOWLEDGMENTS

This study was supported by the March of Dimes Prematurity Research Center at Stanford University School of Medicine, Stanford Maternal & Child Health Research Institute, the Christopher Hess Research Fund, the National Institutes of Health grants 1R01HL139844 and R35GM138353, Burroughs Wellcome Fund, the Alfred E. Mann Foundation, and grants from the Bill & Melinda Gates Foundation OPP1112382, OPP1113682, and INV037517. M.P.S. was supported by National Institutes of Health, grant 5RM1HG00773507. D.A.R. was supported by the Thomas C. and Joan M. Merigan Endowment at Stanford University and the Chan Zuckerberg Biohub Microbiome Initiative.

AUTHOR CONTRIBUTIONS

I.M. and N.A. designed machine-learning experiments and performed interpretation of results; I.M. performed computational analysis and wrote the paper; N.A. provided guidance and feedback and contributed to writing the paper; K.C. performed mass spectrometric metabolomic analysis and contributed to metabolomic pathway analysis and writing of the paper; M.N.M. performed cfRNA transcriptome analysis; I.A.S., D.F., and X.H. performed immunome analysis and contributed to writing the paper; A.T. performed part of the computational analysis; N.S. assisted with the computational analysis; R.J.W. supervised the study data collection and edited the manuscript; G.M.T. and M.E. performed MS metabolomic analysis; N.S., A.L.C., R.F., H.N., M.B., M.X., C.E., D.D.F., M.S.G., and A.C. helped with editing the manuscript; E.K.C. performed microbiome analysis; X.B.L., K.G.S., G.L.D., D.A.R., S.R.Q., and M.S.A. provided critical feedback on the study and edited the manuscript; D.A.R. was involved with the microbiome analysis; M.P.S. was involved in the MS metabolome analysis; V.D.W. wrote a part of the paper and contributed to interpretation of results; G.M.S. provided the study data, provided guidance and feedback, contributed to interpretation of results, and edited the manuscript; B.G. designed the immunome part of the analysis, performed interpretation of the results, and edited the manuscript; D.K.S. provided the study data, provided guidance, and supervised the research. All authors discussed results and contributed to the final manuscript.

DECLARATION OF INTERESTS

S.R.Q. is a founder, consultant, and shareholder of Mirvie. M.N.M. is a shareholder of Mirvie. D.A.R. is a shareholder of Blue Willow Biologics, Cantata Bio, Evelo Biosciences, Karius, Proderm IQ, and Second Genome. D.A.R. is an advisor to Cantata Bio and Visby Medical. K.G.S. is paid advisor of Mission Biocapital and Infinant Health; equity holder and paid advisor of Avexegen; and equity holder of mProbe. M.P.S. is a co-founder and scientific advisor of Personalis, SensOmics, Qbio, January AI, Fodsel, Filtricine, Protos, RTHM, Iollo, Marble Therapeutics, and Mirvie, and a scientific advisor of Genapsys, Jupiter, Neuvivo, Swaza, and Mitrix.

Received: September 27, 2022
Revised: September 28, 2022
Accepted: November 11, 2022
Published: December 9, 2022

REFERENCES

1. WHO; UNICEF; UNFPA; World Bank Group and the United Nations Population Division (2019). Maternal Mortality: Levels and Trends, 2000 to 2017 (World Health Organization).
2. Duley, L. (2009). The global impact of pre-eclampsia and eclampsia. Semin. Perinatol. *33*, 130–137.
3. Jeyabalan, A. (2013). Epidemiology of preeclampsia: impact of obesity. Nutr. Rev. *71* (*Suppl 1*), S18–S25.
4. Than, N.G., Romero, R., Tarca, A.L., Kekesi, K.A., Xu, Y., Xu, Z., Juhasz, K., Bhatti, G., Leavitt, R.J., Gelencser, Z., et al. (2018). Integrated systems biology approach identifies novel maternal and placental pathways of preeclampsia. Front. Immunol. *9*, 1661.
5. Phipps, E.A., Thadhani, R., Benzing, T., and Karumanchi, S.A. (2019). Pre-eclampsia: pathogenesis, novel diagnostics and therapies. Nat. Rev. Nephrol. *15*, 275–289.
6. Chaiworapongsa, T., Chaemsaithong, P., Yeo, L., and Romero, R. (2014). Pre-eclampsia part 1: current understanding of its pathophysiology. Nat. Rev. Nephrol. *10*, 466–480.
7. Duckitt, K., and Harrington, D. (2005). Risk factors for pre-eclampsia at antenatal booking: systematic review of controlled studies. BMJ *330*, 565.
8. Tranquilli, A.L., Brown, M.A., Zeeman, G.G., Dekker, G., and Sibai, B.M. (2013). The definition of severe and early-onset preeclampsia. Statements from the international society for the study of hypertension in pregnancy (ISSHP). Pregnancy Hypertens. *3*, 44–47.
9. Ghaemi, M.S., DiGiulio, D.B., Contrepois, K., Callahan, B., Ngo, T.T.M., Lee-McMullen, B., Lehallier, B., Robaczewska, A., Mcilwain, D., Rosenberg-Hasson, Y., et al. (2019). Multiomics modeling of the

immunome, transcriptome, microbiome, proteome and metabolome adaptations during human pregnancy. Bioinformatics *35*, 95–103.

10. Benny, P.A., Alakwaa, F.M., Schlueter, R.J., Lassiter, C.B., and Garmire, L.X. (2020). A review of omics approaches to study preeclampsia. Placenta *92*, 17–27.

11. Roberge, S., Nicolaides, K., Demers, S., Hyett, J., Chaillet, N., and Bujold, E. (2017). The role of aspirin dose on the prevention of preeclampsia and fetal growth restriction: systematic review and meta-analysis. Am. J. Obstet. Gynecol. *216*, 110–120.e6.

12. American College of Obstetricians and Gynecologists; Task Force on Hypertension in Pregnancy (2013). Hypertension in pregnancy. Report of the American College of obstetricians and gynecologists' task force on hypertension in pregnancy. Obstet. Gynecol. *122*, 1122–1131.

13. Zou, H., and Hastie, T. (2005). Regularization and variable selection via the elastic net. J. Roy. Stat. Soc. B *67*, 301–320.

14. Lam, C., Lim, K.-H., Kang, D.-H., and Karumanchi, S.A. (2005). Uric acid and preeclampsia. Semin. Nephrol. *25*, 56–60.

15. Maynard, S.E., and Karumanchi, S.A. (2011). Angiogenic factors and preeclampsia. Semin. Nephrol. *31*, 33–46.

16. Taylor, B.D., Ness, R.B., Olsen, J., Hougaard, D.M., Skogstrand, K., Roberts, J.M., and Haggerty, C.L. (2015). Serum leptin measured in early pregnancy is higher in women with preeclampsia compared with normotensive pregnant women. Hypertension *65*, 594–599.

17. Ma, H.Y., Cu, W., Sun, Y.H., and Chen, X. (2020). MiRNA-203a-3p inhibits inflammatory response in preeclampsia through regulating IL24. Eur. Rev. Med. Pharmacol. Sci. *24*, 5223–5230.

18. Meinshausen, N., and Bühlmann, P. (2010). Stability selection. J. Roy. Stat. Soc. B *72*, 417–473.

19. Redman, C.W.G., and Sargent, I.L. (2010). Immunology of pre-eclampsia. Am. J. Reprod. Immunol. *63*, 534–543.

20. Han, X., Ghaemi, M.S., Ando, K., Peterson, L.S., Ganio, E.A., Tsai, A.S., Gaudilliere, D.K., Stelzer, I.A., Einhaus, J., Bertrand, B., et al. (2019). Differential dynamics of the maternal immune system in healthy pregnancy and preeclampsia. Front. Immunol. *10*, 1305.

21. Samolis, S., Papastefanou, I., Panagopoulos, P., Galazios, G., Kouskoukis, A., and Maroulis, G. (2010). Relation between first trimester maternal serum leptin levels and body mass index in normotensive and pre-eclamptic pregnancies–role of leptin as a marker of pre-eclampsia: a prospective case-control study. Gynecol. Endocrinol. *26*, 338–343.

22. Hashimoto, M., Shinozuka, K., Bjur, R.A., Westfall, D.P., Hattori, K., and Masumura, S. (1995). The effects of age on the release of adenine nucleosides and nucleotides from rat caudal artery. J. Physiol. (Lond.) *489*, 841–848.

23. Marić, I., Tsur, A., Aghaeepour, N., Montanari, A., Stevenson, D.K., Shaw, G.M., and Winn, V.D. (2020). Early prediction of preeclampsia via machine learning. Am. J. Obstet. Gynecol. MFM *2*, 100100. https://doi.org/10.1016/j.ajogmf.2020.100100.

24. Chatuphonprasert, W., Jarukamjorn, K., and Ellinger, I. (2018). Physiology and pathophysiology of steroid biosynthesis, transport and metabolism in the human placenta. Front. Pharmacol. *9*, 1027.

25. Liang, L., Rasmussen, M.H., Piening, B., Shen, X., Chen, S., Röst, H., Snyder, J.K., Tibshirani, R., Skotte, L., Lee, N.C., et al. (2020). Metabolic dynamics and prediction of gestational age and time to delivery in pregnant women. Cell *181*, 1680–1692.e15.

26. Handelman, S.K., Romero, R., Tarca, A.L., Pacora, P., Ingram, B., Maymon, E., Chaiworapongsa, T., Hassan, S.S., and Erez, O. (2019). The plasma metabolome of women in early pregnancy differs from that of non-pregnant women. PLoS One *14*, e0224682. https://doi.org/10.1371/journal.pone.0224682.

27. Nilsen, R.M., Bjørke-Monsen, A.L., Midttun, O., Nygård, O., Pedersen, E.R., Ulvik, A., Magnus, P., Gjessing, H.K., Vollset, S.E., and Ueland, P.M. (2012). Maternal tryptophan and kynurenine pathway metabolites and risk of preeclampsia. Obstet. Gynecol. *119*, 1243–1250.

28. Luppi, P., Tse, H., Lain, K.Y., Markovic, N., Piganelli, J.D., and DeLoia, J.A. (2006). Preeclampsia activates circulating immune cells with engagement of the NF-kappaB pathway. Am. J. Reprod. Immunol. *56*, 135–144.

29. Rumpf, S., and Jentsch, S. (2006). Functional division of substrate processing cofactors of the ubiquitin-selective Cdc48 chaperone. Mol. Cell *21*, 261–269.

30. Trifonova, E.A., Gabidulina, T.V., Ershov, N.I., Serebrova, V.N., Vorozhishcheva, A.Y., and Stepanov, V.A. (2014). Analysis of the placental tissue transcriptome of normal and preeclampsia complicated pregnancies. Acta Naturae *6*, 71–83.

31. Massobrio, M., Alexander, B.T., Bennett, W.A., and Khalil, R.A. (1988). Arachidonic acid derivatives in the pathophysiology of pregnancy-induced hypertension. Clin. Exp. Hypertens. - Part B Hypertens. Pregnancy *7*, 43–55.

32. Badawy, A.A.-B. (2015). Tryptophan metabolism, disposition and utilization in pregnancy. Biosci. Rep. *35*.

33. Tarca, A.L., Romero, R., Benshalom-Tirosh, N., Than, N.G., Gudicha, D.W., Done, B., Pacora, P., Chaiworapongsa, T., Panaitescu, B., Tirosh, D., et al. (2019). The prediction of early preeclampsia: results from a longitudinal proteomics study. PLoS One *14*, e0217273.

34. Austdal, M., Tangerås, L.H., Skråstad, R.B., Salvesen, K., Austgulen, R., Iversen, A.C., and Bathen, T.F. (2015). First trimester urine and serum metabolomics for prediction of preeclampsia and gestational hypertension: a prospective screening study. Int. J. Mol. Sci. *16*, 21520–21538.

35. Docheva, N., Romero, R., Chaemsaithong, P., Tarca, A.L., Bhatti, G., Pacora, P., Panaitescu, B., Chaiyasit, N., Chaiworapongsa, T., Maymon, E., et al. (2019). The profiles of soluble adhesion molecules in the "great obstetrical syndromes". J. Matern. Fetal Neonatal Med. *32*, 2113–2136.

36. Gotsch, F., Romero, R., Friel, L., Kusanovic, J.P., Espinoza, J., Erez, O., Than, N.G., Mittal, P., Edwin, S., Yoon, B.H., et al. (2007). CXCL10/IP-10: a missing link between inflammation and anti-angiogenesis in preeclampsia? J. Matern. Fetal Neonatal Med. *20*, 777–792.

37. Chen, J., Yue, C., Xu, J., Zhan, Y., Zhao, H., Li, Y., and Ye, Y. (2019). Downregulation of receptor tyrosine kinase-like orphan receptor 1 in preeclampsia placenta inhibits human trophoblast cell proliferation, migration, and invasion by PI3K/AKT/mTOR pathway accommodation. Placenta *82*, 17–24.

38. Wang, N., Li, R., and Xue, M. (2019). Potential regulatory network in the PSG10P/miR-19a-3p/IL1RAP pathway is possibly involved in preeclampsia pathogenesis. J. Cell Mol. Med. *23*, 852–864.

39. Zhang, Y., Cao, L., Jia, J., Ye, L., Wang, Y., Zhou, B., and Zhou, R. (2019). CircHIPK3 is decreased in preeclampsia and affects migration, invasion, proliferation, and tube formation of human trophoblast cells. Placenta *85*, 1–8.

40. Løset, M., Mundal, S.B., Johnson, M.P., Fenstad, M.H., Freed, K.A., Lian, I.A., Eide, I.P., Bjørge, L., Blangero, J., Moses, E.K., and Austgulen, R. (2011). A transcriptional profile of the decidua in preeclampsia. Am. J. Obstet. Gynecol. *204* 84.e1-27.

41. Maynard, S.E., Min, J.Y., Merchan, J., Lim, K.H., Li, J., Mondal, S., Libermann, T.A., Morgan, J.P., Sellke, F.W., Stillman, I.E., et al. (2003). Excess placental soluble fms-like tyrosine kinase 1 (sFlt1) may contribute to endothelial dysfunction, hypertension, and proteinuria in preeclampsia. J. Clin. Invest. *111*, 649–658.

42. Rath, G., and Tripathi, R. (2012). Angiogenic balance and diagnosis of preeclampsia: selecting the right VEGF receptor. J. Hum. Hypertens. *26*, 207–210.

43. Verlohren, S., Herraiz, I., Lapaire, O., Schlembach, D., Zeisler, H., Calda, P., Sabria, J., Markfeld-Erol, F., Galindo, A., Schoofs, K., et al. (2014). New gestational phase-specific cutoff values for the use of the soluble fms-like tyrosine kinase-1/placental growth factor ratio as a diagnostic test for preeclampsia. Hypertension *63*, 346–352.

44. Pérez-Pérez, A., Toro, A., Vilariño-García, T., Maymó, J., Guadix, P., Dueñas, J.L., Fernández-Sánchez, M., Varone, C., and Sánchez-

CellPress
OPEN ACCESS

Margalet, V. (2018). Leptin action in normal and pathological pregnancies. J. Cell Mol. Med. *22*, 716–727.

45. Naylor, C., and Petri, W.A. (2016). Leptin regulation of immune responses. Trends Mol. Med. *22*, 88–98.

46. Abella, V., Scotece, M., Conde, J., Pino, J., Gonzalez-Gay, M.A., Gómez-Reino, J.J., Mera, A., Lago, F., Gómez, R., Gualillo, O., et al. (2017). Leptin in the interplay of inflammation, metabolism and immune system disorders. Nat. Rev. Rheumatol. *13*, 100–109.

47. Martín-Romero, C., Santos-Alvarez, J., Goberna, R., and Sánchez-Margalet, V. (2000). Human leptin enhances activation and proliferation of human circulating T lymphocytes. Cell. Immunol. *199*, 15–24.

48. Ivetic, A., Hoskins Green, H.L., and Hart, S. (2019). J. L-selectin: a major regulator of leukocyte adhesion, migration and signaling. Front. Immunol. *10*, 1068.

49. Seidelin, J.B., Nielsen, O.H., and Strøm, J. (2002). Soluble L-selectin levels predict survival in sepsis. Intensive Care Med. *28*, 1613–1618.

50. Chavarría, M.E., Lara-González, L., García-Paleta, Y., Vital-Reyes, V.S., and Reyes, A. (2008). Adhesion molecules changes at 20 gestation weeks in pregnancies complicated by preeclampsia. Eur. J. Obstet. Gynecol. Reprod. Biol. *137*, 157–164.

51. Sabatier, F., Bretelle, F., D'ercole, C., Boubli, L., Sampol, J., and Dignat-George, F. (2000). Neutrophil activation in preeclampsia and isolated intrauterine growth restriction. Am. J. Obstet. Gynecol. *183*, 1558–1563.

52. Rainer, T.H. (2002). L-selectin in health and disease. Resuscitation *52*, 127–141.

53. Sonnweber, T., Pizzini, A., Nairz, M., Weiss, G., and Tancevski, I. (2018). Arachidonic acid metabolites in cardiovascular and metabolic diseases. Int. J. Mol. Sci. *19*, 3285.

54. Walsh, S.W. (2004). Eicosanoids in preeclampsia. Prostaglandins Leukot. Essent. Fatty Acids *70*, 223–232.

55. Mills, J.L., DerSimonian, R., Raymond, E., Morrow, J.D., Roberts, L.J., 2nd, Clemens, J.D., Hauth, J.C., Catalano, P., Sibai, B., Curet, L.B., and Levine, R.J. (1999). Prostacyclin and thromboxane changes predating clinical onset of preeclampsia: a multicenter prospective study. JAMA *282*, 356–362.

56. Cui, Y., Zhu, B., and Zheng, F. (2018). Low-dose aspirin at ≤16 weeks of gestation for preventing preeclampsia and its maternal and neonatal adverse outcomes: a systematic review and meta-analysis. Exp. Ther. Med. *15*, 4361–4369.

57. Chang, R.-Q., Li, D.-J., and Li, M.-Q. (2018). The role of indoleamine-2,3-dioxygenase in normal and pathological pregnancies. Am. J. Reprod. Immunol. *79*, e12786.

58. Munn, D.H., Zhou, M., Attwood, J.T., Bondarev, I., Conway, S.J., Marshall, B., Brown, C., and Mellor, A.L. (1998). Prevention of allogeneic fetal rejection by tryptophan catabolism. Science *281*, 1191–1193.

59. DiNatale, B.C., Murray, I.A., Schroeder, J.C., Flaveny, C.A., Lahoti, T.S., Laurenzana, E.M., Omiecinski, C.J., and Perdew, G.H. (2010). Kynurenic acid is a potent endogenous aryl hydrocarbon receptor ligand that synergistically induces interleukin-6 in the presence of inflammatory signaling. Toxicol. Sci. *115*, 89–97.

60. Nguyen, N.T., Kimura, A., Nakahama, T., Chinen, I., Masuda, K., Nohara, K., Fujii-Kuriyama, Y., and Kishimoto, T. (2010). Aryl hydrocarbon receptor negatively regulates dendritic cell immunogenicity via a kynurenine-dependent mechanism. Proc. Natl. Acad. Sci. USA *107*, 19961–19966.

61. Mezrich, J.D., Fechner, J.H., Zhang, X., Johnson, B.P., Burlingham, W.J., and Bradfield, C.A. (2010). An interaction between kynurenine and the aryl hydrocarbon receptor can generate regulatory T cells. J. Immunol. *185*, 3190–3198.

62. Jaronen, M., and Quintana, F.J. (2014). Immunological relevance of the coevolution of Ido1 and AHR. Front. Immunol. *5*, 521.

63. Fazio, F., Lionetto, L., Curto, M., Iacovelli, L., Copeland, C.S., Neale, S.A., Bruno, V., Battaglia, G., Salt, T.E., and Nicoletti, F. (2017). Cinnabarinic acid and xanthurenic acid: two kynurenine metabolites that interact with metabotropic glutamate receptors. Neuropharmacology *112*, 365–372.

64. Santillan, M.K., Pelham, C.J., Ketsawatsomkron, P., Santillan, D.A., Davis, D.R., Devor, E.J., Gibson-Corley, K.N., Scroggins, S.M., Grobe, J.L., Yang, B., et al. (2015). Pregnant mice lacking indoleamine 2,3-dioxygenase exhibit preeclampsia phenotypes. Physiol. Rep. *3*, e12257.

65. Wright, D., Syngelaki, A., Akolekar, R., Poon, L.C., and Nicolaides, K.H. (2015). Competing risks model in screening for preeclampsia by maternal characteristics and medical history. Am. J. Obstet. Gynecol. *213*, 62.e1–62.e10.

66. Odibo, A.O., Zhong, Y., Goetzinger, K.R., Odibo, L., Bick, J.L., Bower, C.R., and Nelson, D.M. (2011). First-trimester placental protein 13, PAPP-A, uterine artery Doppler and maternal characteristics in the prediction of pre-eclampsia. Placenta *32*, 598–602.

67. Yu, C.K.H., Smith, G.C., Papageorghiou, A.T., Cacho, A.M., and Nicolaides, K.H. (2005). An integrated model for the prediction of pre-eclampsia using maternal factors and uterine artery Doppler velocimetry in unselected low-risk women. Am. J. Obstet. Gynecol. *193*, 429–436.

68. Audibert, F., Boucoiran, I., An, N., Aleksandrov, N., Delvin, E., Bujold, E., and Rey, E. (2010). Screening for preeclampsia using first-trimester serum markers and uterine artery Doppler in nulliparous women. Am. J. Obstet. Gynecol. *203*, 383.e1–383.e8.

69. Wright, D., Wright, A., and Nicolaides, K.H. (2019). The competing risk approach for prediction of preeclampsia. Am. J. Obstet. Gynecol. *223*, 12–23.e7. https://doi.org/10.1016/j.ajog.2019.11.1247.

70. North, R.A., McCowan, L.M., Dekker, G.A., Poston, L., Chan, E.H., Stewart, A.W., Black, M.A., Taylor, R.S., Walker, J.J., Baker, P.N., and Kenny, L.C. (2011). Clinical risk prediction for pre-eclampsia in nulliparous women: development of model in international prospective cohort. BMJ *342*, d1875.

71. Parra-Cordero, M., Rodrigo, R., Barja, P., Bosco, C., Rencoret, G., Sepúlveda-Martinez, A., and Quezada, S. (2013). Prediction of early and late pre-eclampsia from maternal characteristics, uterine artery Doppler and markers of vasculogenesis during first trimester of pregnancy. Ultrasound Obstet. Gynecol. *41*, 538–544.

72. Kelly, R.S., Croteau-Chonka, D.C., Dahlin, A., Mirzakhani, H., Wu, A.C., Wan, E.S., McGeachie, M.J., Qiu, W., Sordillo, J.E., Al-Garawi, A., et al. (2017). Integration of metabolomic and transcriptomic networks in pregnant women reveals biological pathways and predictive signatures associated with preeclampsia. Metabolomics *13*.

73. De Kat, A.C., Hirst, J., Woodward, M., Kennedy, S., and Peters, S.A. (2019). Prediction models for preeclampsia: a systematic review. Pregnancy Hypertens. *16*, 48–66.

74. Stamilio, D.M., Sehdev, H.M., Morgan, M.A., Propert, K., and Macones, G.A. (2000). Can antenatal clinical and biochemical markers predict the development of severe preeclampsia? Am. J. Obstet. Gynecol. *182*, 589–594.

75. Chaiworapongsa, T., Romero, R., Korzeniewski, S.J., Kusanovic, J.P., Soto, E., Lam, J., Dong, Z., Than, N.G., Yeo, L., Hernandez-Andrade, E., et al. (2013). Maternal plasma concentrations of angiogenic/antiangiogenic factors in the third trimester of pregnancy to identify the patient at risk for stillbirth at or near term and severe late preeclampsia. Am. J. Obstet. Gynecol. *208*, 287.e1–287.e15.

76. Recht, B., Roelofs, R., Schmidt, L., and Shankar, V. (2019). Do ImageNet classifiers generalize to ImageNet?. Preprint at arXiv. https://doi.org/10.48550/arXiv.1902.10811.

77. Aghaeepour, N., Ganio, E.A., Mcilwain, D., Tsai, A.S., Tingle, M., Van Gassen, S., Gaudilliere, D.K., Baca, Q., McNeil, L., Okada, R., et al. (2017). An immune clock of human pregnancy. Sci. Immunol. *2*, eaan2946.

78. Breiman, L. (1996). Stacked regressions. Mach. Learn. *24*, 49–64.

79. Wolpert, D.H. (1992). Stacked generalization. Neural Network *5*, 241–259.

80. Stone, M. (1974). Cross-validatory choice and assessment of statistical predictions. J. Roy. Stat. Soc. B *36*, 111–133.

81. Chzhen, E., Hebiri, M., and Salmon, J. (2019). On Lasso refitting strategies. Bernoulli *25*, 3175–3200.

82. Hastie, T., Tibshirani, R., and Wainwright, M. (2015). In Statistical Learning with Sparsity: The Lasso and Generalizations (Chapman and Hall/CRC), pp. 155–182. https://doi.org/10.1201/b18401-8.

83. van der Maaten, L. (2008). Visualizing high-dimensional data using t-SNE. J. Mach. Learn. Res.

84. Tang, J., Liu, J., Zhang, M., and Mei, Q. (2016). Visualizing large-scale and high-dimensional data. In Proceedings of the 25th International Conference on World Wide Web - WWW '16 (ACM Press), pp. 287–297. https://doi.org/10.1145/2872427.2883041.

85. Benjamini, Y., and Hochberg, Y. (1995). Controlling the false discovery rate: a practical and powerful approach to multiple testing. J. Roy. Stat. Soc. B *57*, 289–300.

86. Pang, Z., Chong, J., Li, S., and Xia, J. (2020). Metaboanalystr 3.0: toward an optimized workflow for global metabolomics. Metabolites *10*, 186.

87. Ashburner, M., Ball, C.A., Blake, J.A., Botstein, D., Butler, H., Cherry, J.M., Davis, A.P., Dolinski, K., Dwight, S.S., Eppig, J.T., et al. (2000). Gene Ontology: tool for the unification of biology. Nat. Genet. *25*, 25–29.

88. The Gene Ontology Consortium (2019). The gene ontology resource: 20 years and still GOing strong. Nucleic Acids Res. *47*, D330–D338.

89. Kuznetsova, I., Lugmayr, A., Siira, S.J., Rackham, O., and Filipovska, A. (2019). CirGO: an alternative circular way of visualising gene ontology terms. BMC Bioinf. *20*, 84.

Patterns, Volume *3*

Supplemental information

Early prediction and longitudinal modeling of preeclampsia from multiomics

Ivana Marić, Kévin Contrepois, Mira N. Moufarrej, Ina A. Stelzer, Dorien Feyaerts, Xiaoyuan Han, Andy Tang, Natalie Stanley, Ronald J. Wong, Gavin M. Traber, Mathew Ellenberger, Alan L. Chang, Ramin Fallahzadeh, Huda Nassar, Martin Becker, Maria Xenochristou, Camilo Espinosa, Davide De Francesco, Mohammad S. Ghaemi, Elizabeth K. Costello, Anthony Culos, Xuefeng B. Ling, Karl G. Sylvester, Gary L. Darmstadt, Virginia D. Winn, Gary M. Shaw, David A. Relman, Stephen R. Quake, Martin S. Angst, Michael P. Snyder, David K. Stevenson, Brice Gaudilliere, and Nima Aghaeepour

Supplemental Experimental Procedures

Content:

Tables S1 to S5

Figures S1 to S22

Supplemental Experimental Procedures

Table S1. Patient and pregnancy characteristics

	Discovery Cohort 1 (N=33)		Validation Cohort (N=16)	
**Demographics**	*Controls (N=16)*	*Preeclampsia (N=17)*	*Controls (N=4)*	*Preeclampsia (N=12)*
**Maternal age at enrollment**				
(years, mean ± SD)	32.1 ± 4.9	31.1 ± 6.3	30.7 ± 4.8	32.3 ± 4.5
**Gravida** (N, % nulliparous)	7 (43.7)	6 (35.3)	2 (50)	5 (41.7)
**Ethnicity** (N, %)				
Hispanic	0 (0)	8 (47)	0 (0)	2 (16.7)
Non-Hispanic	16 (100)	9 (53)	4 (100)	9 (75)
Unknown	0 (0)	0 (0)	0 (0)	1 (8.3)
**Race (N, %)**				
White	16 (100)	9 (52.9)	4 (100)	5 (41.7)
African-American	0 (0)	1 (6.0)	0 (0)	1 (8.3)
Asian	0 (0)	4 (23.5)	0 (0)	4 (33.3)
Unknown	0 (0)	3 (17.6)	0 (0)	1 (8.3)
Other	0 (0)	0 (0)	0 (0)	1 (8.3)
**Preexisting hypertension**	0 (0)	4 (23.5)	0 (0)	4 (33.3)
**Height**				
(cm, mean ± SD)	166.9 ± 7.4	158.8 ± 6.2	163 ± 3.5	163.8 ± 7.7
**Weight**				
(kg, mean ± SD)	61.9 ± 9.1	74.0 ± 20.3	62.6 ± 8.1	79.6 ± 25.9
**BMI** (mean ± SD)	22.8 ± 3.3	29.4 ± 7.9	23.5 ± 2.5	29.4 ± 7.7
**Multiple gestation** (N, %)	0 (0)	2	0 (0)	0 (0)
**Baby gender (male N, %)**	8 (50)	11 (64.7)	4 (100)	6 (50)

Table S2. Preeclampsia patient characteristics.		
	**Cohort 1 (n=17)**	**Validation Cohort (n=12)**
**Gestational age at the onset of preeclampsia** (mean ± SD)	35.8 ± 3.8	36.6 ± 3.7
**Early Onset** (N, %)	5 (29.4)	1 (8.3)
**Severe preeclampsia** (N, %)	10 (58.8)	7 (58.3)

Table S3. List of annotated urine metabolites selected in EN models.

Compound ID	Mode	Molecular Ion	Metabolite	Formula	KEGG	HMDB	MSI annotation level
368.2789_8.5	pRPLC	[M+H]+	C14:2 AC (Tetradecadiencarnitine)	C21H37NO4		HMDB 13331	2
249.0074_2.8	nRPLC	[M-H]-	Dihydroxyphenylglycol O-sulfate	C8H10O7S		HMDB 01474	3
153.0193_1.5	nRPLC	[M-H]-	Dihydroxybenzoic acid	C7H6O4		HMDB 13676	2
298.2009_5.1	pRPLC	[M+H]+	C9:2 AC (Nonadienoylcarnitine)	C16H27NO4			2
632.2045_0.8	nRPLC	[M-H]-	Sialyllactose	C23H39NO19		HMDB 00825	2
263.0231_4.8	nRPLC	[M-H]-	Methoxy hydroxyphenylethyleneglycol sulfate	C9H12O7S		HMDB 00559	3
359.0984_3.9	nRPLC	[M-H]-	Methoxy hydroxyphenylglycol glucuronide	C15H20O10	C03033	HMDB 00496	3
136.0614_0.9	pRPLC	[M+H]+	Adenine	C5H5N5	C00147	HMDB 00034	1
385.2366_8.1	pRPLC	[M+H-H2O]+	Dehydrocholic acid	C24H34O5			2
189.1598_17.5	pHILIC	[M+H]+	N6,N6,N6-Trimethyl-L-lysine	C9H20N2O2	C03793	HMDB 01325	1
131.0713_2.3	nRPLC	[M-H]-	C6:0,OH FA (Hydroxyhexanoic acid)	C6H12O3		HMDB 00409	2
425.0804_12.3	nHILIC	[M-H]-	Cysteineglutathione disulfide	C13H22N4O8S2		HMDB 00656	3

232.1178_3.5	pRPLC	[M+H]+	Isovalerylglutamic acid	C10H17NO5		HMDB 00726	3
352.1246_3.4	pRPLC	[M+H]+	N-Acetyl-O-acetylneuraminic acid	C13H21NO10		HMDB 60492	3
289.2159_8.8	pRPLC	[M-H]-	Dehydroepiandrosterone	C19H28O2	C01227	HMDB 00077	3
169.1235_7.9	nRPLC	[M-H]-	C10:1 FA (Decenoic acid)	C10H18O2		HMDB 41012	2
189.0767_3.1	nRPLC	[M-H]-	C8:0, OH DC FA (Hydroxysuberic acid)	C8H14O5		HMDB 00325	3
176.1029_0.5	pRPLC	[M+H]+	Citrulline	C6H13N3O3	C00327	HMDB 00904	1
299.0631_2	nRPLC	[M-H]-	Uric acid ribonucleoside	C10H12N4O7	C05513	HMDB 29920	3
189.1234_8.2	pHILIC	[M+H]+	N-epsilon-acetyl-L-lysine	C8H16N2O3	C02727	HMDB 00206	1
202.1437_6.8	pRPLC	[M+H]+	N-Acetylaminooctanoic acid	C10H19NO3		HMDB 59745	3
302.2323_6.2	pRPLC	[M+H]+	C9:0 AC (Nonanoylcarnitine)	C16H31NO4		HMDB 13288	2
157.0602_8.2	pHILIC	[M+H]+	Imidazolelactic acid	C6H8N2O3	C05132	HMDB 02320	3
139.0497_0.7	pRPLC	[M+H]+	Nicotinamide N-oxide	C6H6N2O2		HMDB 02730	1
263.023_1.7	nRPLC	[M-H]-	Methoxy hydroxyphenylethyleneglycol sulfate	C9H12O7S		HMDB 00559	3
209.0665_8.5	nHILIC	[M-H]-	1,5-anhydroglucitol (1,5-AG)	C7H14O7	C07326	HMDB 02712	3
314.2324_5.5	pHILIC	[M+H]+	C10:1 AC (Decenoylcarnitine)	C17H31NO4		HMDB 13205	2
230.1034_3.5	nRPLC	[M-H]-	Isovalerylglutamic acid	C10H17NO5		HMDB 00726	3
284.1854_3.9	pRPLC	[M+H]+	C8:2 AC (Octadienoylcarnitine)	C15H25NO4			2
281.1494_2	pHILIC	[M+H]+	Tyr-Val	C14H20N2O4		HMDB 29118	2
342.2634_8	pRPLC	[M+H]+	C12:1 AC (Dodecenoylcarnitine)	C19H35NO4		HMDB 13326	1
314.2323_6.7	pRPLC	[M+H]+	C10:1 AC (Decenoylcarnitine)	C17H31NO4		HMDB 13205	2
153.0547_5	pRPLC	[M+H]+	2-Hydroxyphenylacetic acid	C8H8O3	C05852	HMDB 00669	2
448.3065_4.7	nHILIC	[M-H]-	Glycoursodeoxycholic acid	C26H43NO5		HMDB 00708	3
166.0862_10.4	pHILIC	[M+H]+	Pyridinebutanoic acid	C9H11NO2		HMDB 01007	3

330.227_5.7	pRPLC	[M+H]+	C10:1, OH AC (Hydroxydecenoylcarnitine)	C17H31NO5			2
263.1289_6.1	nRPLC	[M-H]-	gamma-CEHC	C15H20O4		HMDB 01931	2
565.3016_9.4	nRPLC	[M-H-H2O]-	Cholic acid glucuronide	C30H46O10		HMDB 02577	3
286.1396_11.1	pHILIC	[M+H]+	Glycylprolylhydroxyproline	C12H19N3O5		HMDB 02171	3
467.2655_9.8	nRPLC	[M-H]-	5alpha-Androstan-3alpha,17beta-diol 17-glucuronide	C25H40O8			3
258.1698_3.1	pRPLC	[M+H]+	C6:1 AC (Hexenoylcarnitine)	C13H23NO4		HMDB 13161	2
302.2325_5.5	pHILIC	[M+H]+	C9:0 AC (Nonanoylcarnitine)	C16H31NO4		HMDB 13288	2
229.1545_0.5	pRPLC	[M+H]+	N,N,N-trimethyl-alanylproline betaine (TMAP)	C11H20N2O3		HMDB 0240365	2
230.1031_8.4	nHILIC	[M-H]-	Isovalerylglutamic acid	C10H17NO5		HMDB 00726	3
455.2473_12	nRPLC	[M-H]-	Sulfolithocholic acid	C24H40O6S		HMDB 00907	2
360.2744_5.5	pHILIC	[M+H]+	C12:0,OH AC (Hydroxydodecanoylcarnitine)	C19H37NO5		HMDB 13164	2
448.307_9.3	nRPLC	[M-H]-	Glycoursodeoxycholic acid	C26H43NO5		HMDB 00708	3
514.284_4.8	nHILIC	[M-H]-	Taurocholic acid	C26H45NO7S	C05122	HMDB 00036	3
176.103_9.1	pHILIC	[M+H]+	Citrulline	C6H13N3O3	C00327	HMDB 00904	1
129.0658_8.5	pHILIC	[M+H]+	Dihydrothymine	C5H8N2O2	C00906	HMDB 00079	1
100.0757_1.2	pRPLC	[M+H]+	2-Piperidinone	C5H9NO		HMDB 11749	2
375.2888_11	pRPLC	[M+H]+	Hydroxycholenoic acid	C24H38O3		HMDB 00308	3
144.0301_5.9	nHILIC	[M-H]-	Keto-glutaramic acid	C5H7NO4	C00940	HMDB 01552	3

Table S4. Related references to proteins identified by our prediction model.		
**Protein**	**Function**	**Mechanism**
LEP[1-5]	Immune regulatory hormone	Possibly contributes to the aberrant immune signature
VEGFA[6,7]	Angiogenic factor	Lack of VEGF causes endothelial cell dysfunction
SELE[8]	Adhesion molecule	
SELL[9-11]	Marker for inflammation	Several mechanisms possible (conflicting results reported)
ROR1[12]	Tyrosine kinase receptor	Downregulation inhibits human trophoblast cell proliferation, migration, and invasion
CXCL10[13]	Pro-inflammatory and anti-angiogenic chemokine	May reflect enhanced systemic inflammatory response
SPARCL1[14]	Impedes trophoblast migration and invasion	Transcriptional profile revealed downregulation in preeclampsia
IL-24[15]	Cytokine	MiRNA-203a-3p inhibits inflammatory response in preeclampsia by regulating IL24
HIPK3[16]	Impacts biological behavior of trophoblast cells	Affects migration, invasion and proliferation of trophoblast cells

Figures:

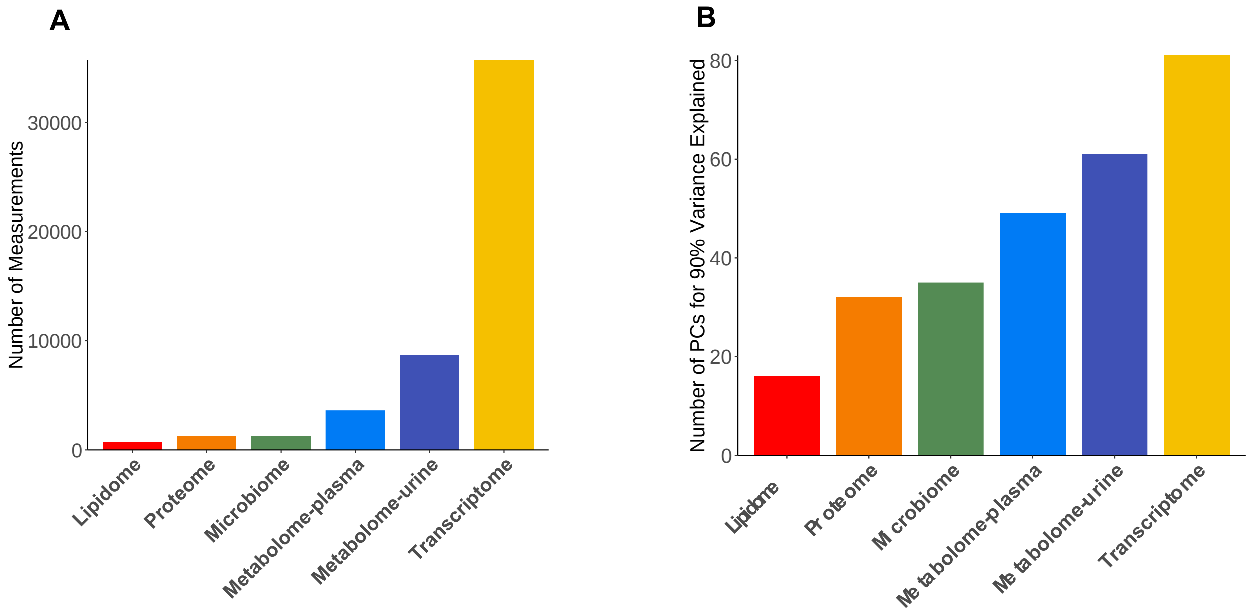

Figure S1. Features of six omics datasets. A. Number of measurements in each dataset; **B.** Number of principal components to account for 90% of the variance. Datasets containing more strongly correlated features yield fewer principal components.

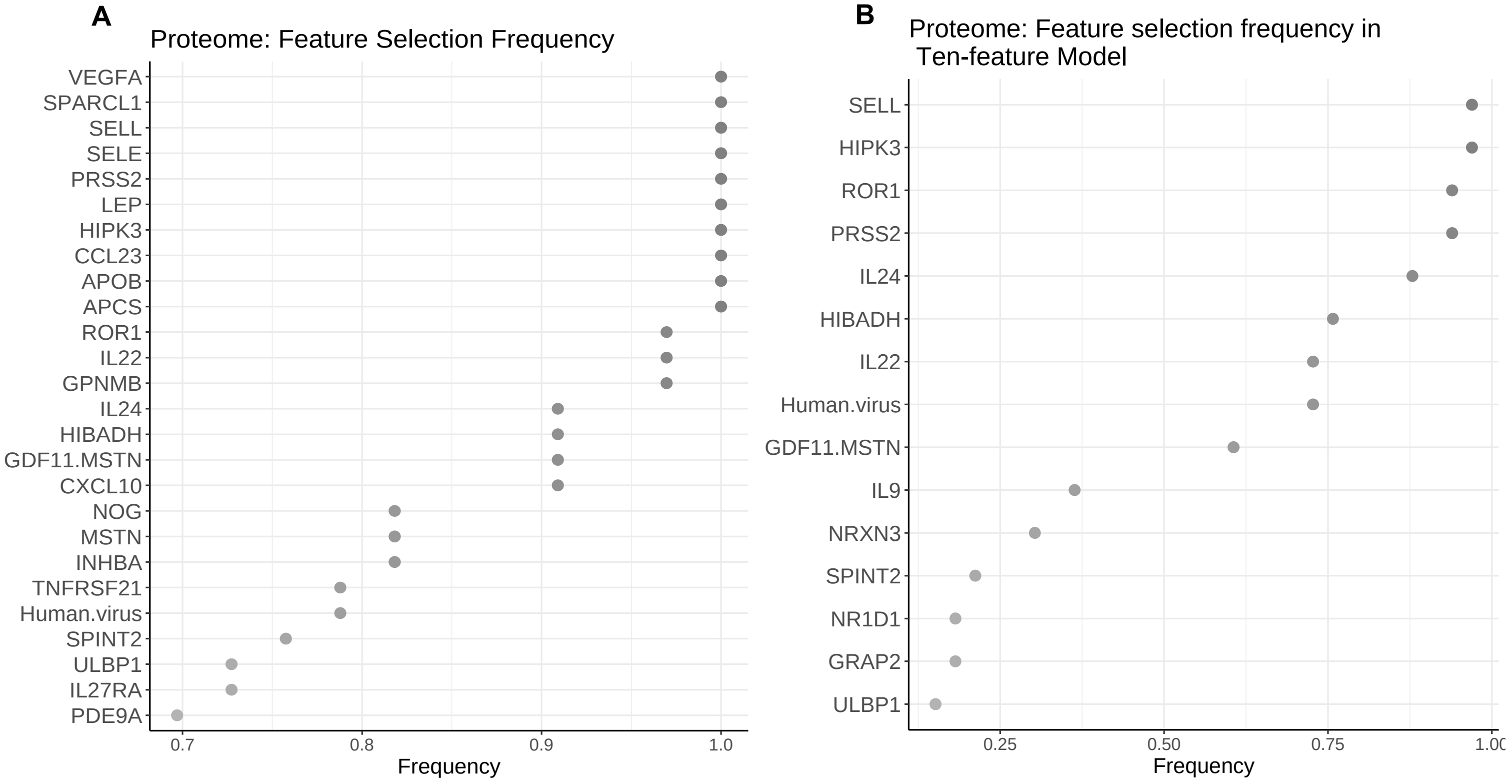

Figure S2. Proteomics feature selection frequency in prediction model over gestation. Each model was obtained using all available samples over gestation. **A. Elastic net model. B. Elastic net model with ten features.** Y-axis shows proteins chosen with the highest frequency across all EN prediction models, where one EN prediction model is built in each cross-validation step. X-axis shows the frequency with which each protein is chosen across all models.

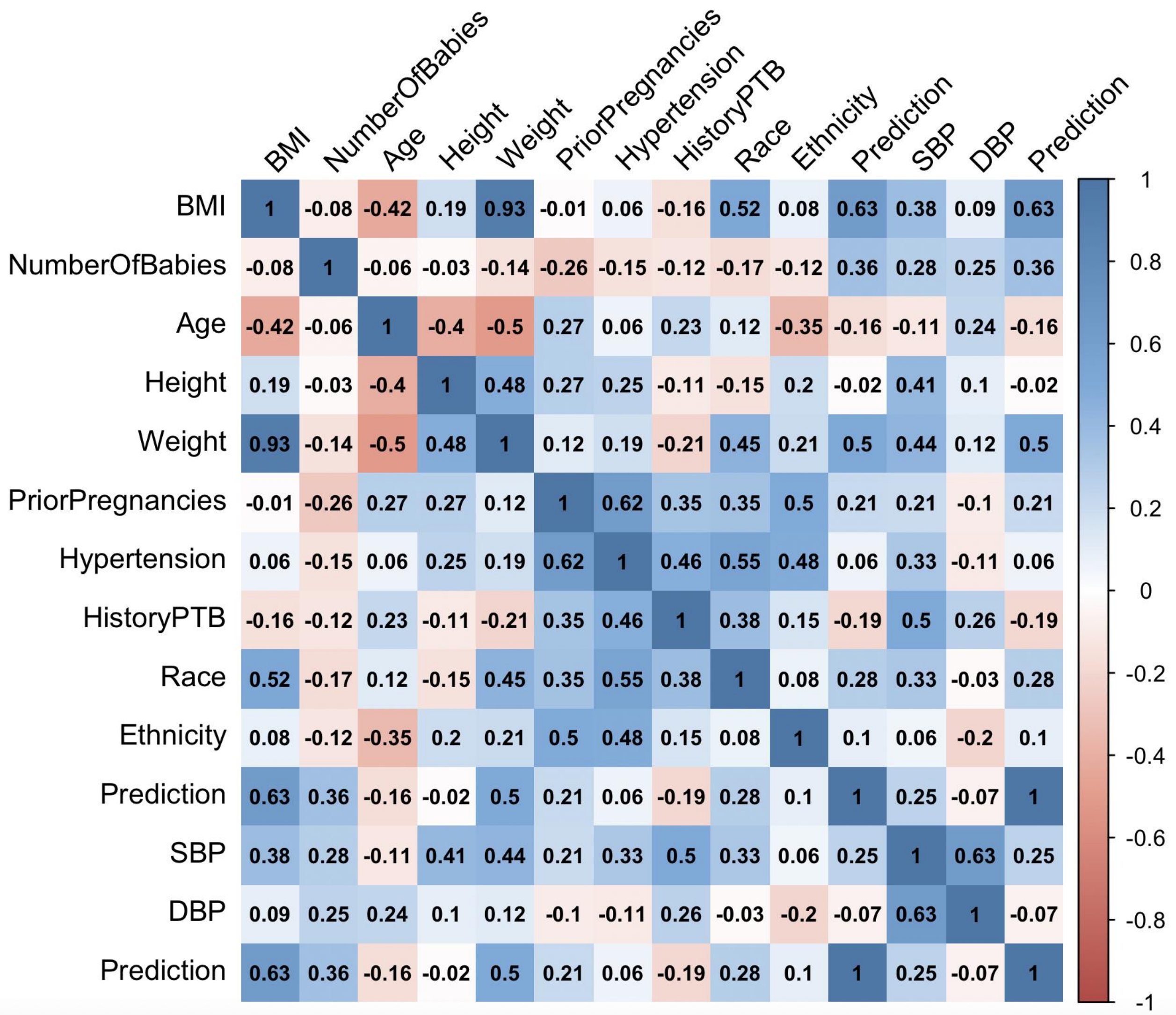

Figure S3. Spearman correlation between predictions obtained from EN model for urine metabolome using available samples over gestation and available clinical variables. The highest correlation, and the only one that was statistically significant was with BMI ($p < 0.0086$).

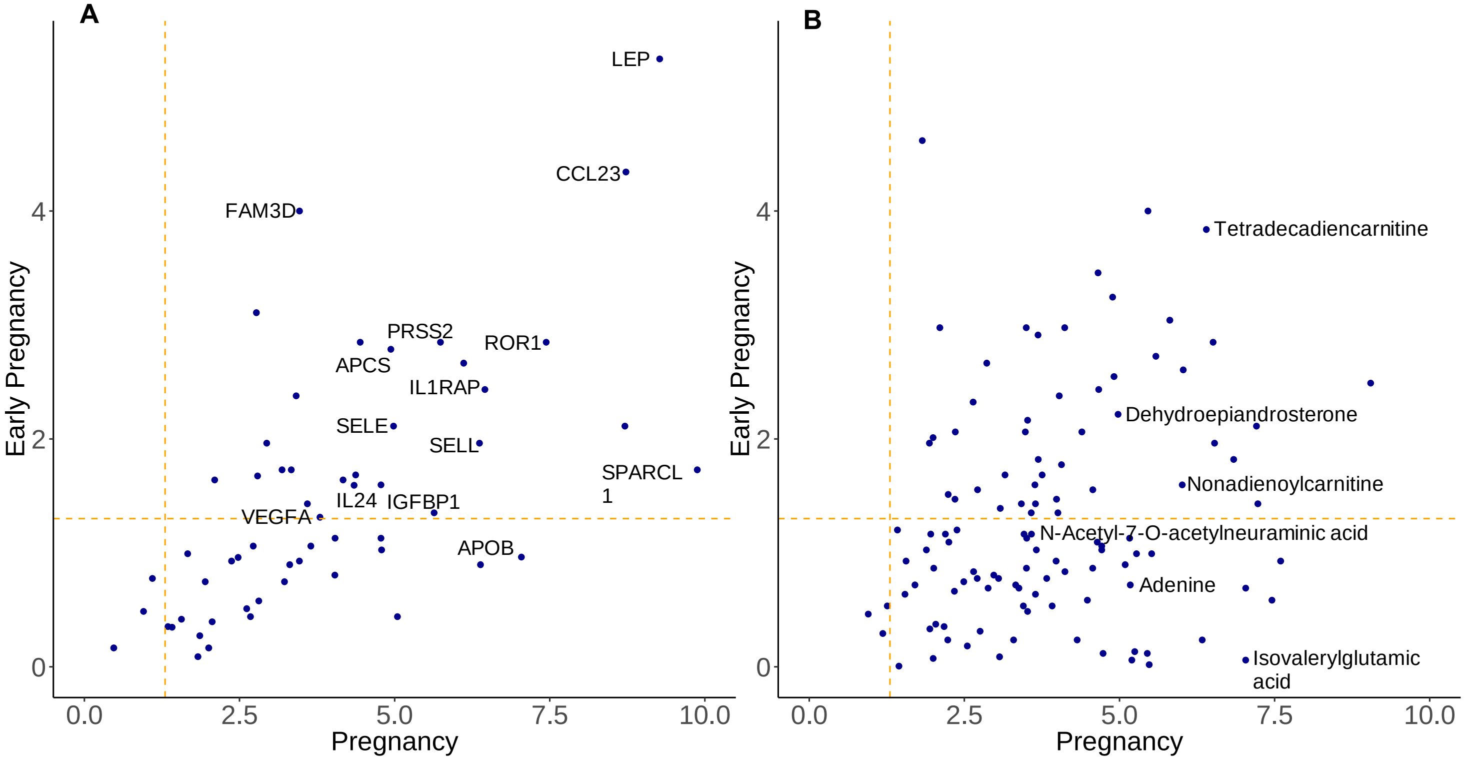

Figure S4. Biomarker comparison: entire pregnancy vs. early pregnancy. X-axis and Y-axis show -log(p-value) of each biomarker in early pregnancy and over gestation. **A.** Most predictive proteins. **B.** Most predictive urine metabolites. All values higher than -log(0.05) indicated by the orange line are significant. We observe that most of the biomarkers regardless of the prediction model are statistically significant over gestation. Less biomarkers are statistically significant in early pregnancy which is expected due to a smaller number of samples.

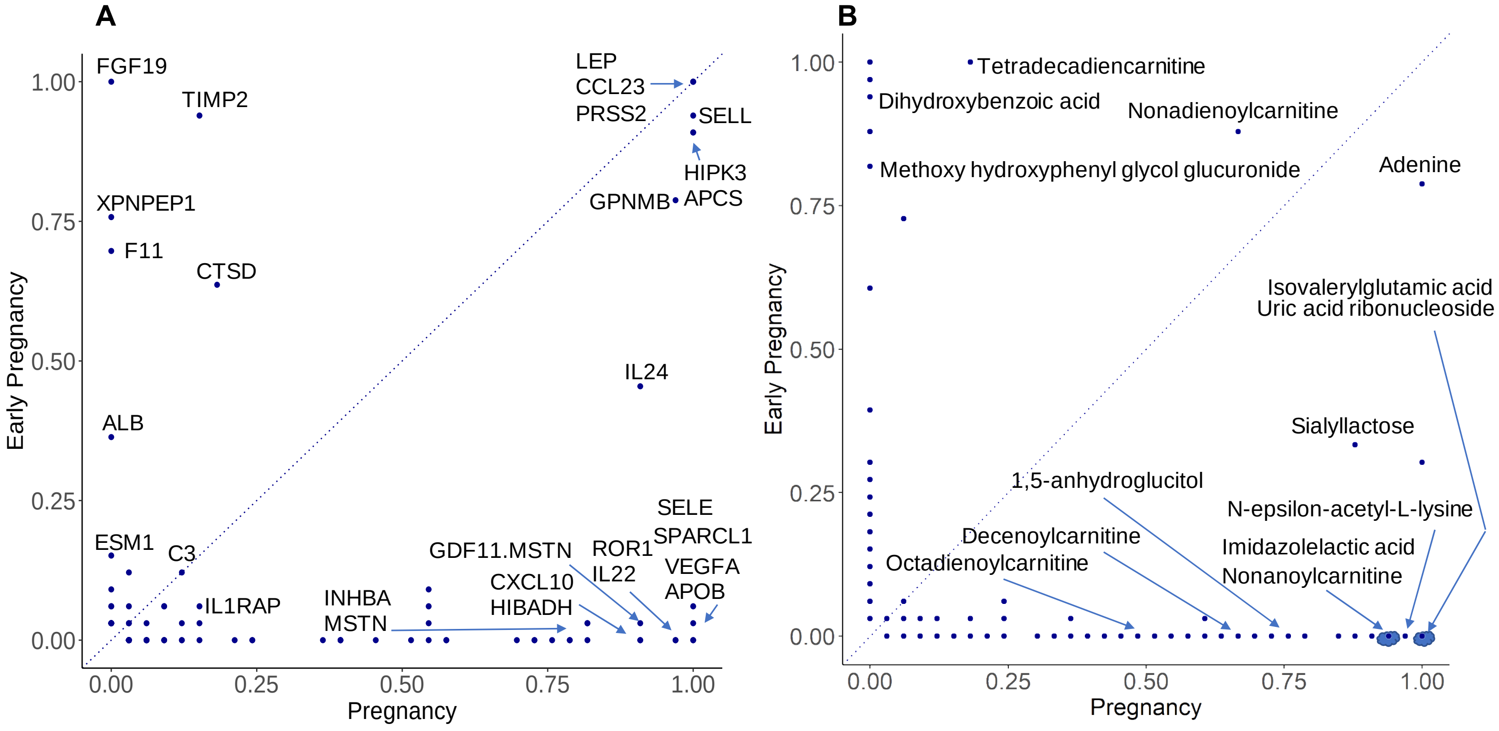

Figure S5. Biomarker comparison: entire pregnancy vs. early pregnancy. X-axis and Y-axis show the respective frequency of each biomarker in early pregnancy and over gestation. **A.** Most predictive proteins. **B.** Most predictive urine metabolites. Blue circles around dots imply the same position for more than one protein/urine metabolite.

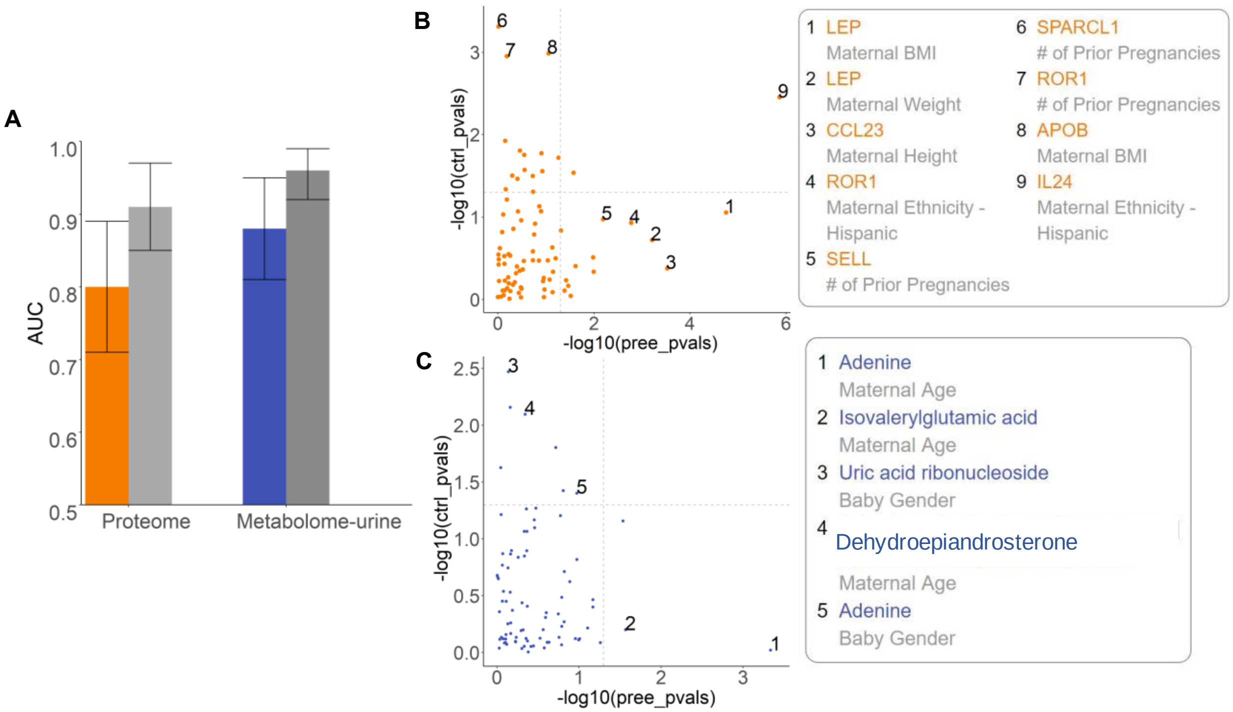

Figure S6. Relationship between urine metabolome and proteome with clinical features over gestation. A. Prediction accuracy of urine metabolome and plasma proteome. Dark blue (for urine metabolome) and orange (for proteome) bars show performance without clinical data (proteome: AUC = 0.83, 95% CI: [0.73, 0.92); urine metabolome: AUC = 0.88, 95% CI [0.81, 0.95]). Grey bars show performance with clinical data (proteome AUC=0.91, 95% CI: [0.85, 0.97]; urine metabolome AUC=0.96, 95% CI: [0.92, 0.99]). **B**. Comparison of P-value of correlations of the top proteome and clinical features. Value of $-\log_{10} P$ for preeclamptic patients and controls is shown on x-axis and y-axis, respectively. Each node is a pair of a proteome and a clinical feature. **C.** Comparison of P-value of correlations of the top urine metabolites and EHR features. Each node is a pair of a proteome/urine metabolome and a clinical feature.

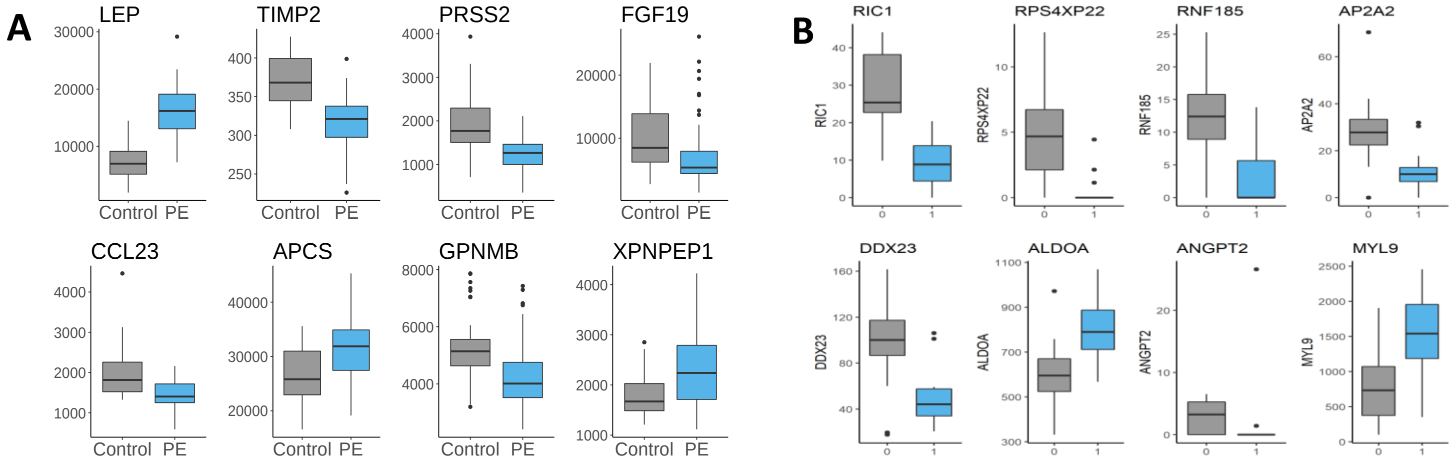

Figure S7. Top ranking proteins and genes identified by prediction models in early pregnancy. **A**. Top-ranking proteins. **B.** Top-ranking genes. Y-axis shows the value in early pregnancy stratified by normal (grey) versus preeclamptic pregnancy (light-blue).

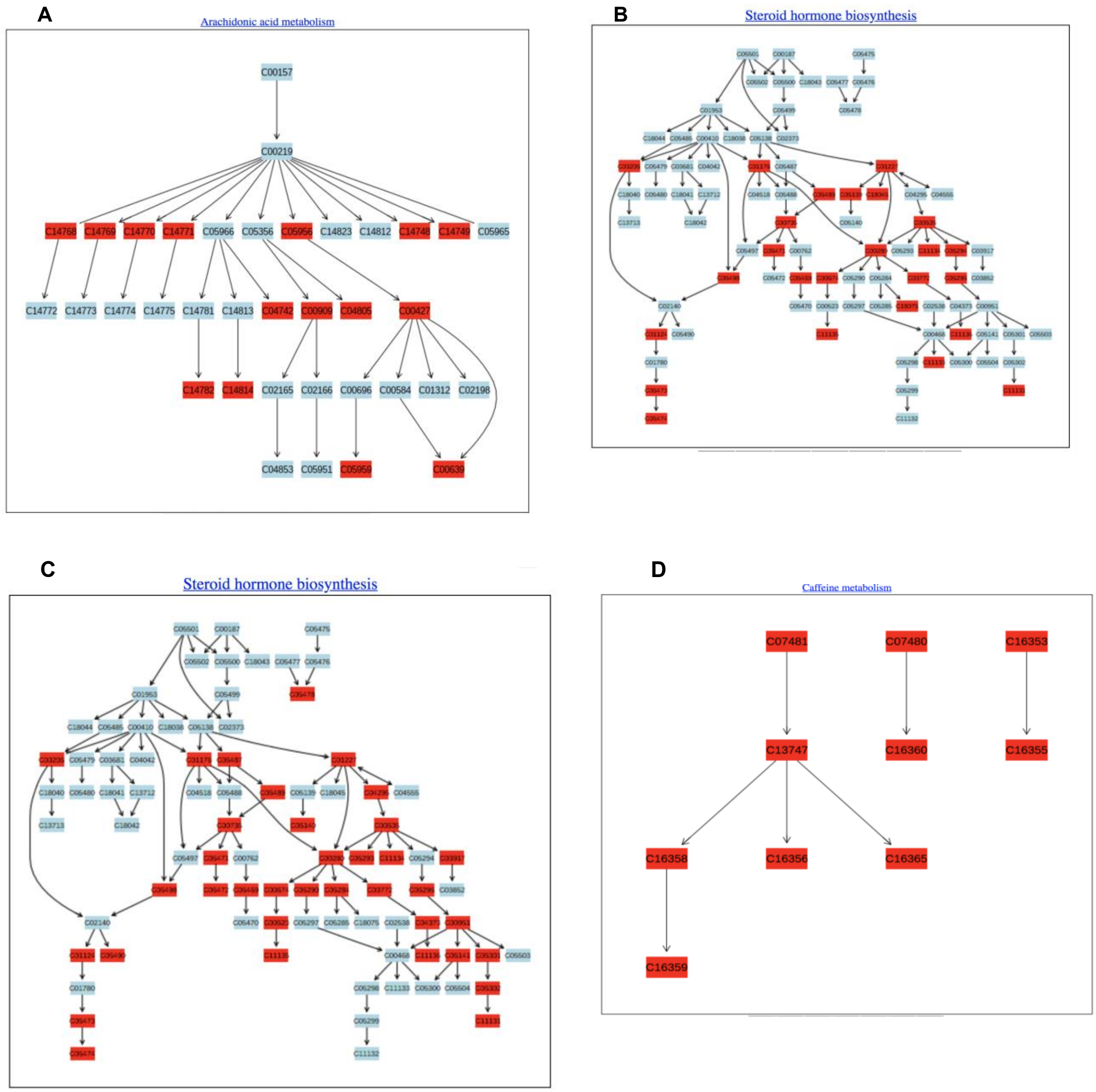

Figure S8. Identified pathways enriched in urine metabolomic data obtained in early pregnancy and over gestation. A. Metabolites in the arachidonic acid pathway in early pregnancy. Metabolites present in the data with high level of significance are shown in red. Metabolites not present in the data are shown in light blue. **B.** Metabolites in the steroid hormone biosynthesis pathway in early pregnancy. **C.** Metabolites in the steroid hormone biosynthesis pathway over gestation. **D**. Metabolites in the caffeine metabolism pathway over gestation.

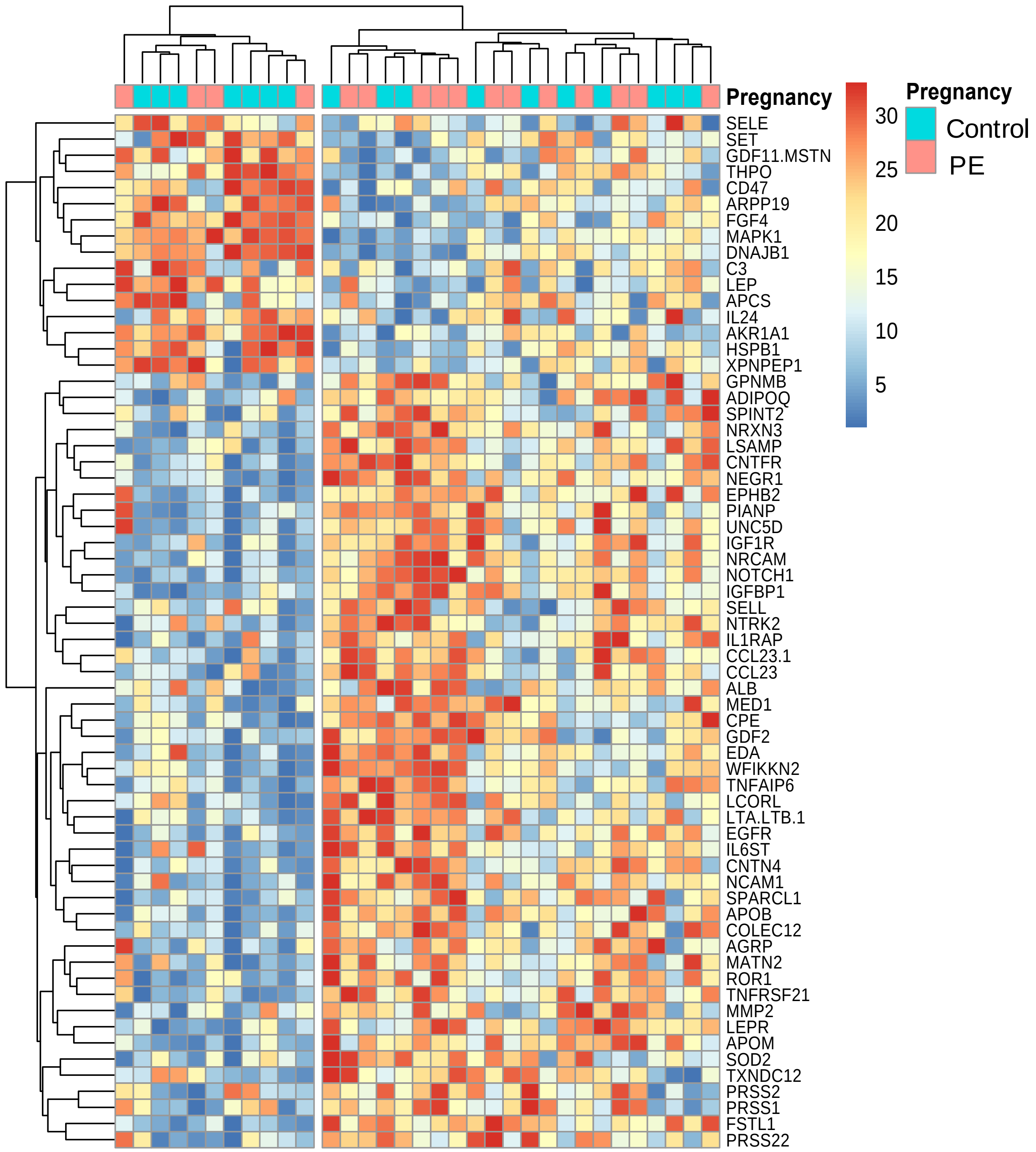

Figure S9. Univariate analysis of proteomic data collected over gestation. Heatmap of the ranked average value of the protein over three trimesters. Changes over gestation of 437 proteins were significantly associated with preeclampsia outcome (Benjamini-Hochberg, FDR < 0.05); 64 proteins with the smallest p-value ($p < 5 \cdot 10^{-5}$, Linear Mixed-Effects Model) are shown.

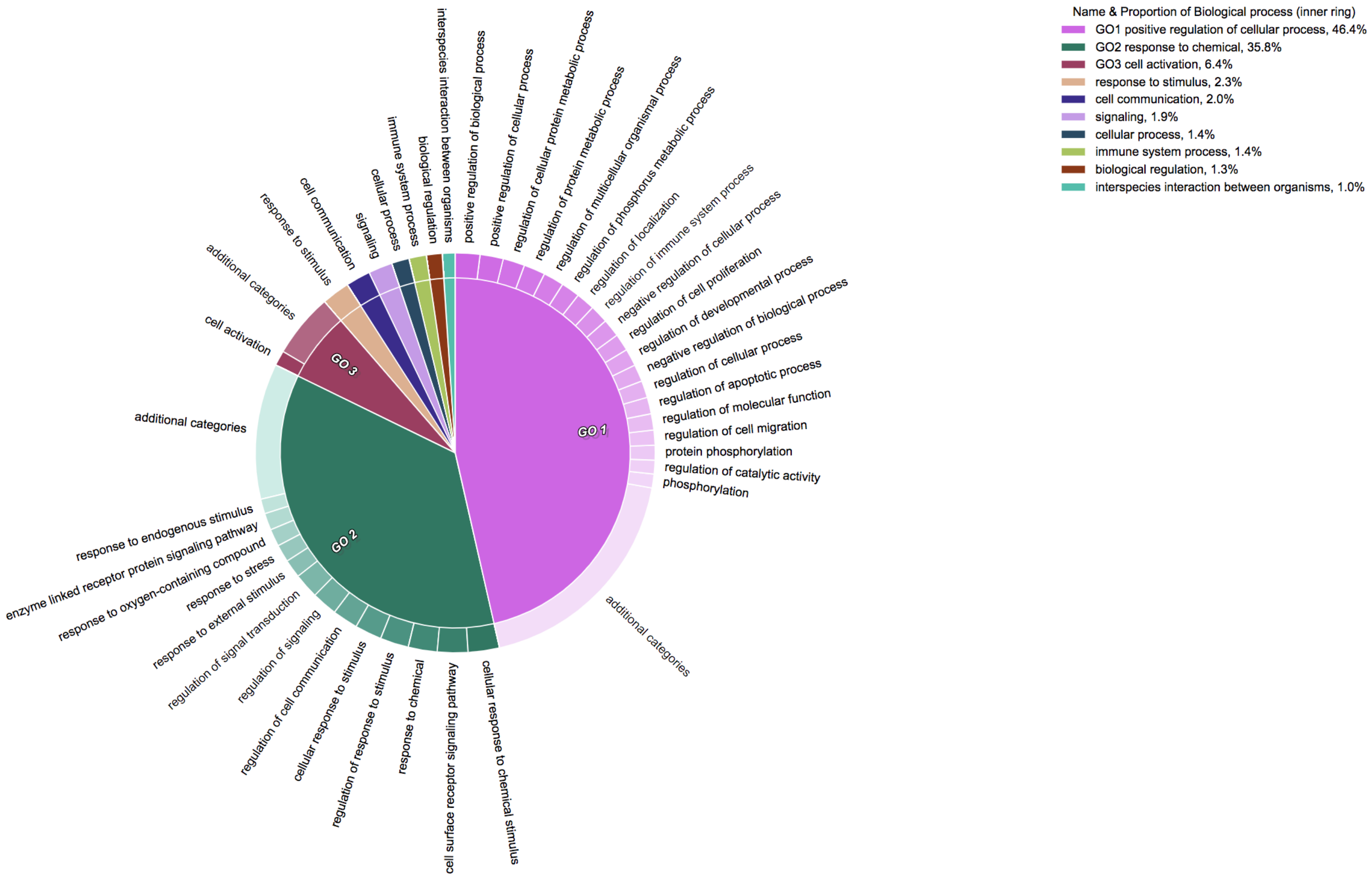

Figure S10. Enriched protein pathways grouped into ten biological processes. Enriched pathways were obtained using all available samples over gestation. The most prevalent biological process was positive regulation of cellular process was (46.4%).

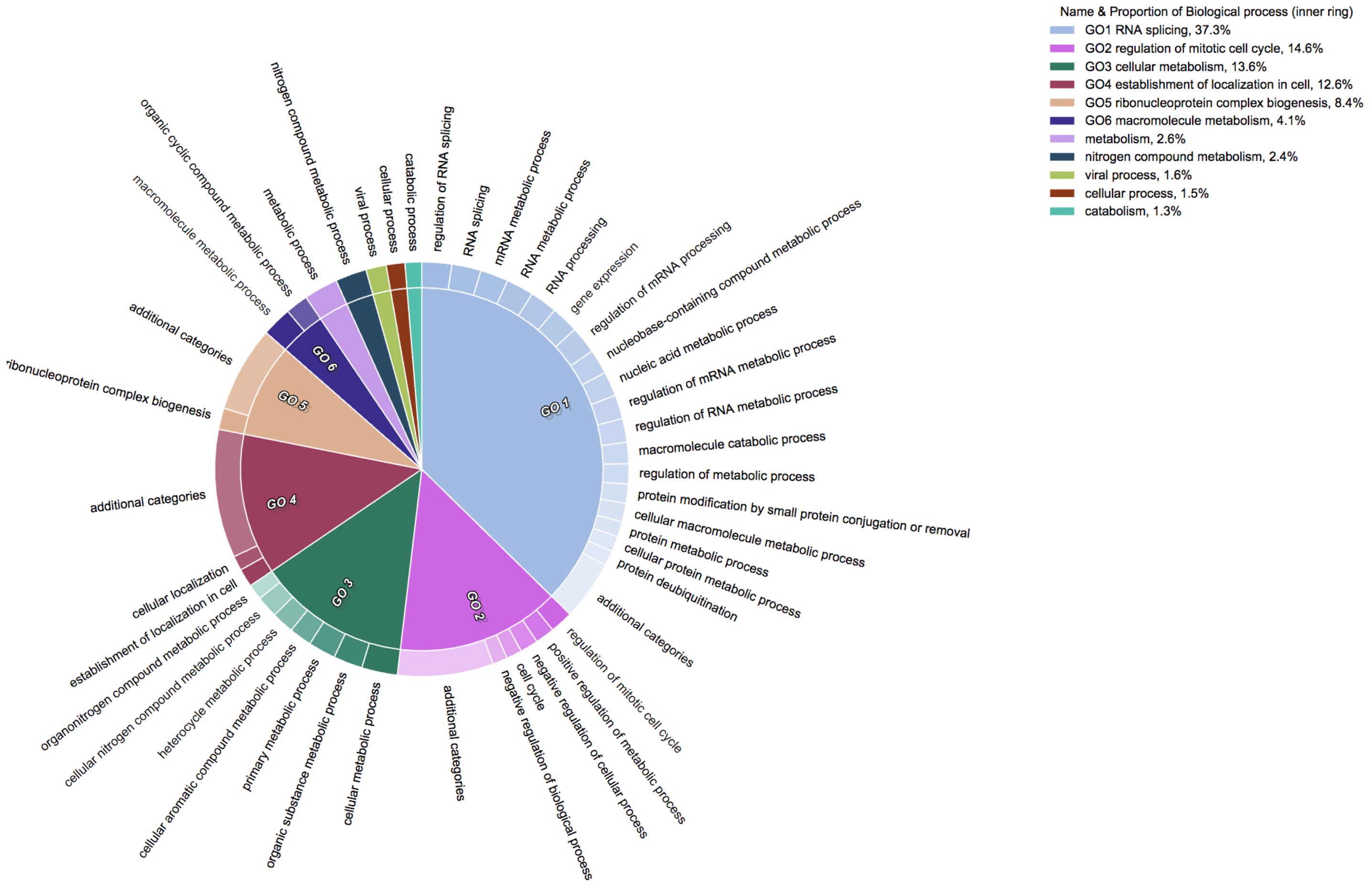

Figure S11. Enriched cfrna pathways in two-level hierarchical structure grouped into eleven biological processes. Enriched pathways were obtained using all available samples over gestation. The most prevalent biological process was RNA splicing (37.3%).

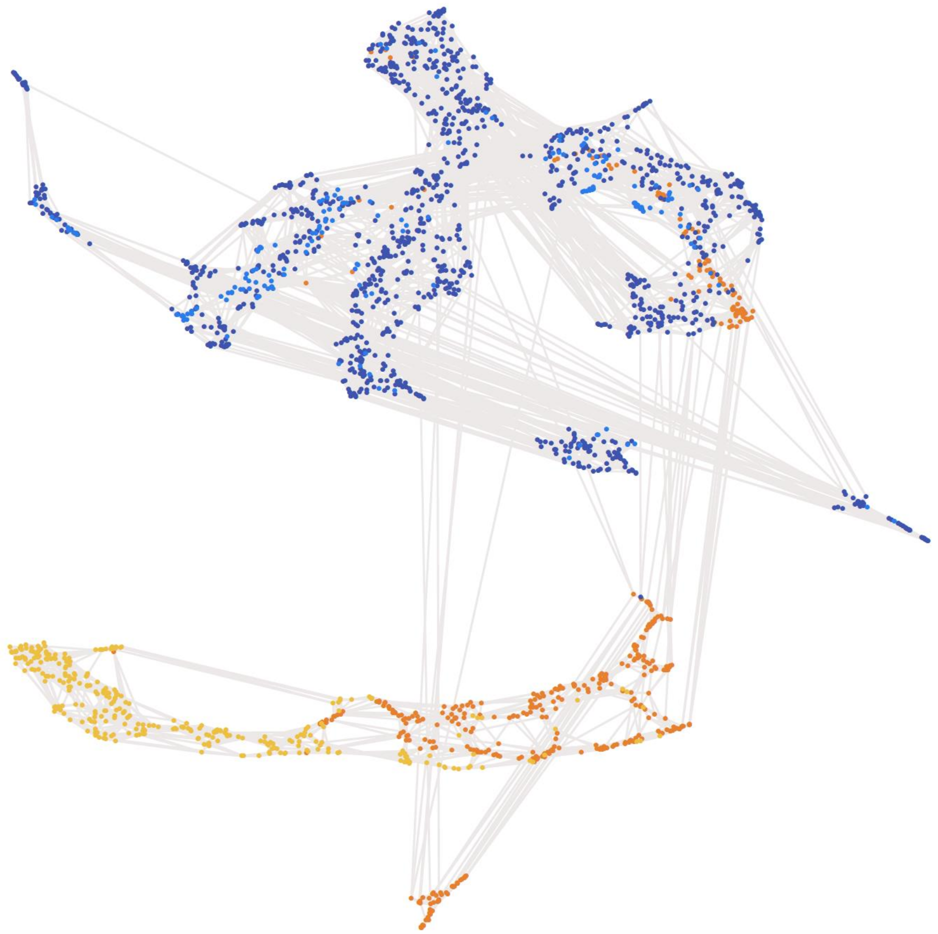

Figure S12. Network of features from different omics sets over gestation. Features with significant association with preeclampsia are shown (FRD<0.05, Linear Mixed-Effects Model with Bonferroni-Hochberg correction). Proteome, urine metabolome, plasma metabolome and transcriptome are shown respectively in orange, dark blue, light blue and yellow. 17 distinct communities were identified.

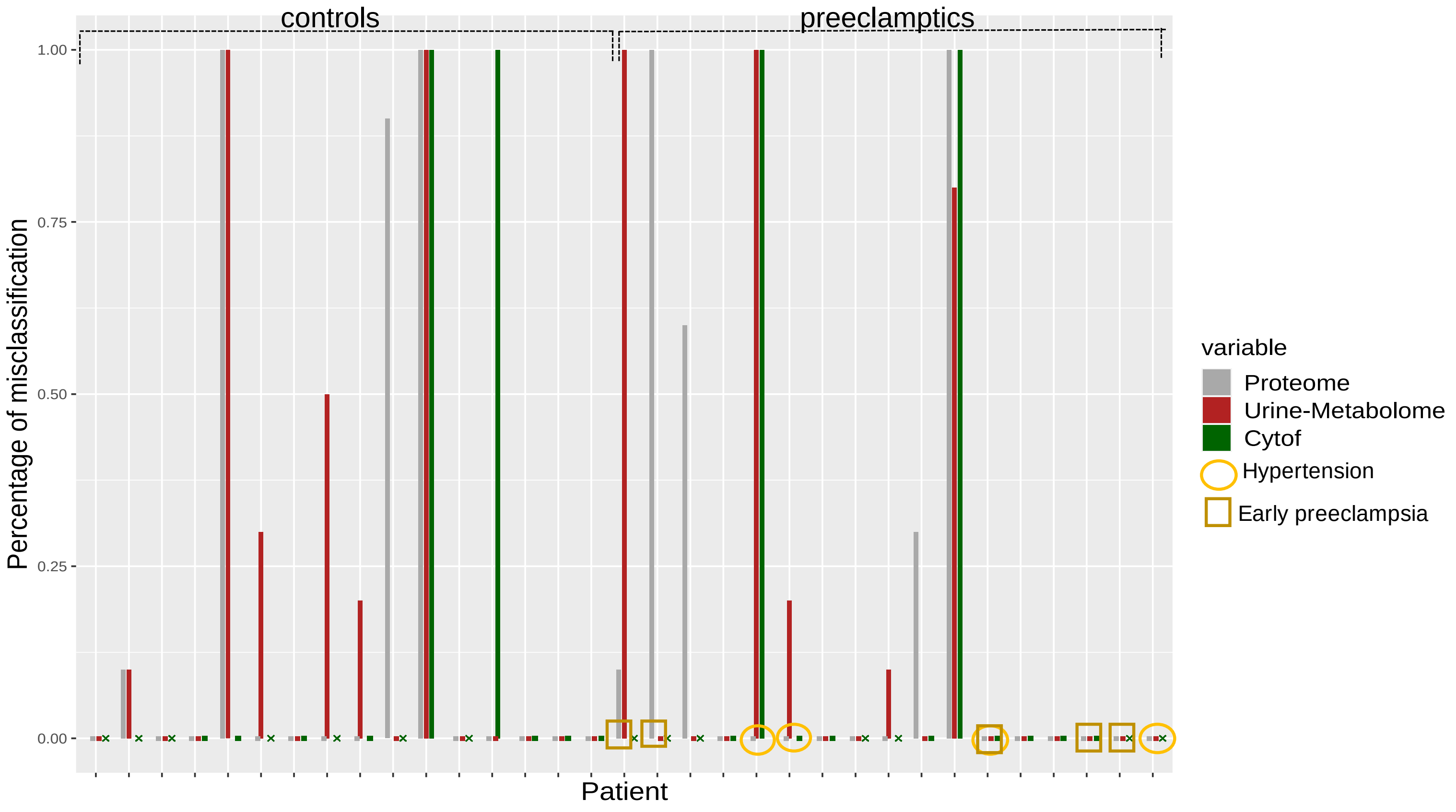

Figure S13. Misclassification rate per each patient in the cohort. Misclassification rate is shown for three prediction algorithms: proteome (gray), urine metabolome (red) and immunome (green).

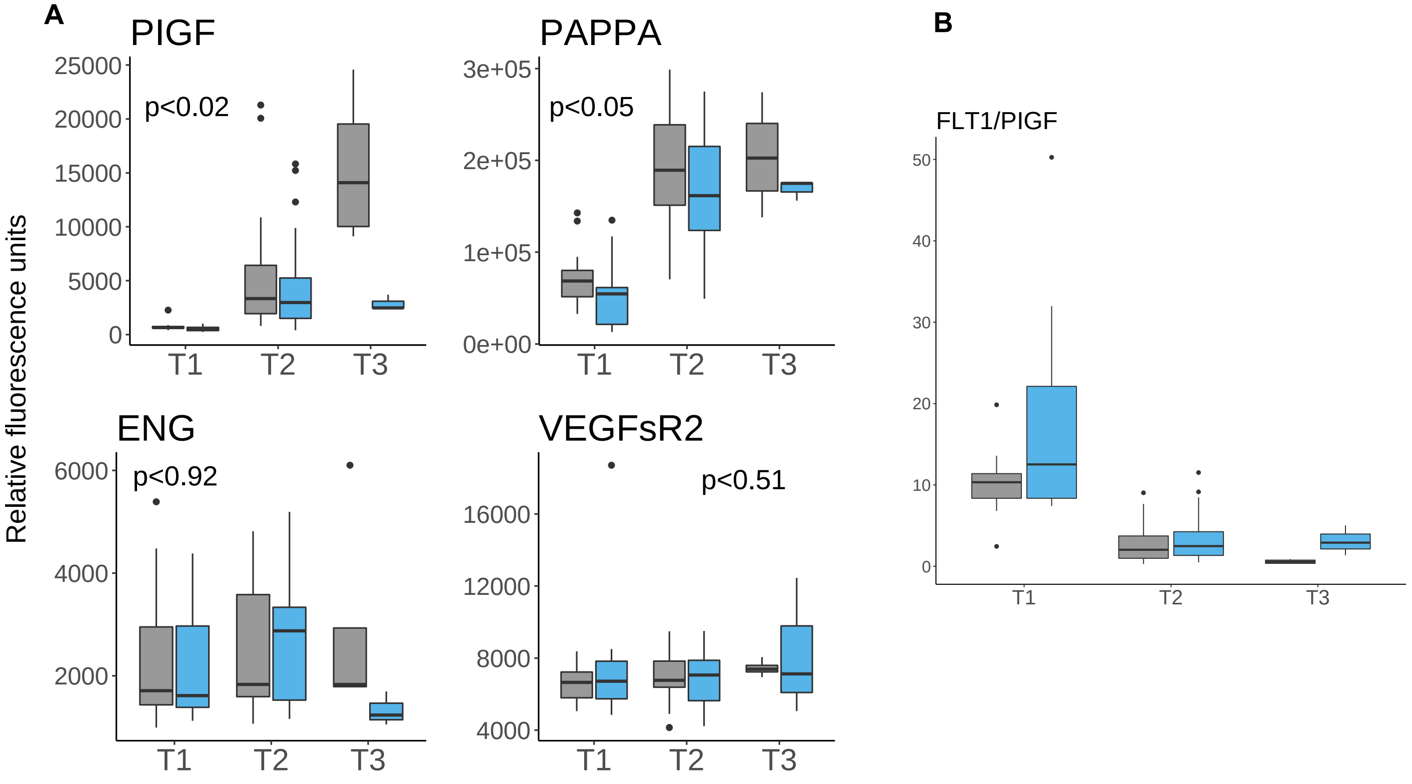

Figure S14. A. Values of known preeclampsia biomarkers over gestation. Values for Placenta growth factor (PlGF), pregnancy-associated plasma protein-A (PAPP-A), endoglin (ENG), vascular endothelial growth factor receptor 2 (VEGFsR2) proteins are shown. For VEGFsR2, the corresponding gene is sFLT-1. Y-axis shows a value stratified by normal pregnancy (grey) and preeclamptic pregnancy (blue). P-value using LME model is shown. PlGF and PAPP-A came as significant. **B.** FLT1/PlGF Ratio.

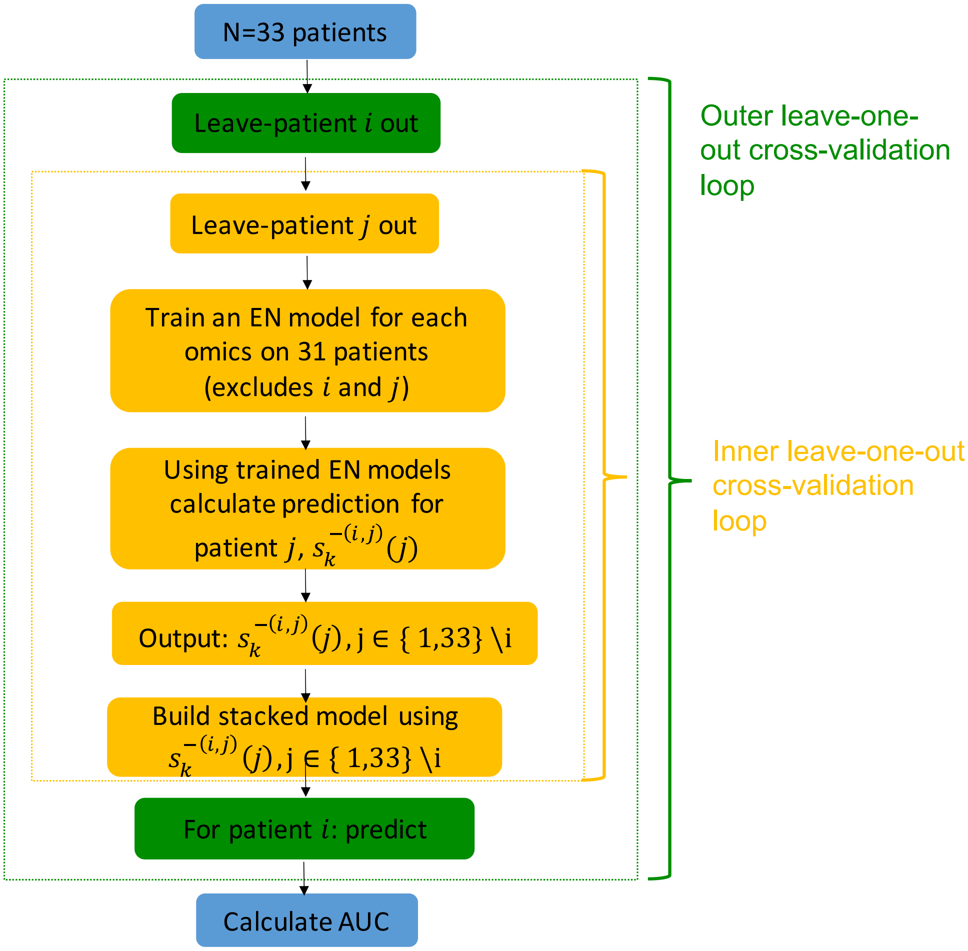

Figure S15. Integration using nested (two-step) cross-validation to build predictive model of preeclampsia using six omics datasets. In each step of cross-validation, EN models for each omics set are first trained and then the stacked model is trained in the same step. After the stacked model is built, it is tested on the test patient that was left out in the outer cross-validation loop. Therefore, no leakage of information between training and test data occurred.

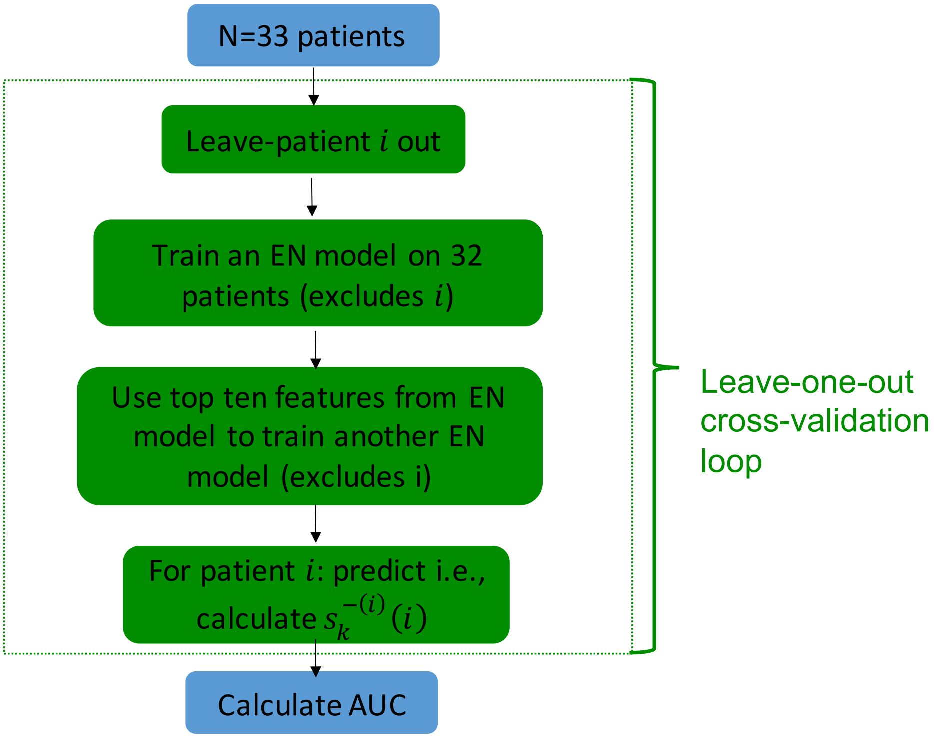

Figure S16. Algorithm for an EN prediction model using top ten features. In each cross-validation step, EN model is trained and then a regression model is trained based on ten features chosen by EN in the same step. The refitted model is then tested on the test patient that was left out in the cross-validation loop. Therefore, no leakage of information between training and test data occurred.

Supplemental Experimental Procedures

1. Cell-free RNA Transcriptome

Cell-free RNA (cfRNA) was extracted from 1 mL of plasma using Plasma/Serum Circulating RNA and Exosomal Purification kit (Norgen, cat 29500) following manufacturer instructions. Residual DNA was digested using BaselineZERO DNase (Epicentre) and then cleaned using RNA Clean and Concentrator-96 kit (Zymo). RNA was eluted to 12 ul in elution buffer. Libraries were prepared using 4 uL cfRNA and SMARTer Stranded Total RNAseq Kit v2 -Pico Input Mammalian Components (Clontech Cat No 634419) and SMARTer RNA Unique Dual Index Barcodes (Set A, Cat 634452) according to the manufacturer's manual. Short read sequencing was performed using the Illumina NovaSeq (2 × 75 bp) platform to an average depth of 50 million reads per

sample. Raw sequencing reads were trimmed with trimmomatic and then mapped to the human reference genome (hg38) with STAR. Duplicates were removed by Picard and then unique reads were quantified using htseq-count. Mapping quality statistics were aggregated using MultiQC.

To estimate RNA degradation, we first counted the number of reads per exon and annotated each exon with its corresponding gene ID and exon number using htseq-count. We then counted the number of genes for which all reads mapped exclusively to the 3' most exon per sample and divided by the total number of genes detected to obtain the fraction of genes where all reads mapped to the 3' most exon.

Finally, we estimated ribosomal read fraction by counting the number of reads that mapped to the ribosomal region (GL00220.1:105424-118780) using samtools view.

Dataset quality is described in the parallel work[17]. Briefly, for every sequenced sample, we estimated three quality parameters were estimated as previously described by our group[18,19]. Our final analysis included a subset of all samples that passed pre-defined quality cutoffs, empirically estimated based on ~700 previously sequenced cell-free RNA samples collected from 5 sites across the globe. Finally, we visualized sample quality as a function of the three defined metrics and find that low-quality samples both cluster separately using hierarchical clustering and drive variance using principal component analysis. Both visualizations and further details regarding quality metrics can be found in Moufarrej et. al (Main text, Methods, Fig S1,2)[17].

2. Proteome

Proteomic assay: Blood was collected into EDTA tubes, immediately placed in ice, centrifuged (3,000 rpm), and plasma was removed and transferred into 1.5-ml microfuge tubes. Tubes were then spun at 13,000 rpm for 1 min, plasma was transferred into another set of microfuge tubes and spun again for 1 min. Plasma was stored at -80°C. All processing was completed within 60 min of collection.

All proteomic analyses were performed blinded and in randomly allocated samples by SomaLogic, Inc. (Boulder, CO) using a highly multiplex aptamer-based platform [S20]. The assay quantifies relative concentrations of 1,310 proteins over a wide dynamic range (> 8 log) using chemically-modified aptamers with slow off-rate kinetics (SOMAmer reagents). Each SOMAmer reagent is a unique, high-affinity, single-strand DNA endowed with functional groups mimicking amino acid side chains. Nucleotide signals are quantified using relative florescence on microarrays (Agilent Technologies, Santa Clara, CA). The assay has a historic median intra- and inter-run coefficient of variation of about 5%, and median lower and upper limits of quantification of 3.0 pM and 1.5 nM[20].

Quality control at the sample level included the use of control SOMAmers on the microarray to monitor for differences in hybridization efficiency, and the calculation of the median signal over all SOMAmers to account for technical variability. The resulting hybridization and median scale factors were used for data normalization across samples. Acceptable scale factors ranged between 0.4 and 2.5 based on historic runs. Quality control at the SOMAmer level included the

use of replicate calibrator plasma samples (7) and biological controls (4) to monitor for repeatability and batch-to-batch variability. Historic values were used for each SOMAmer to derive a calibration scale factor. Acceptance criteria were a median scale factor between 0.8 and 1.2, and deviation by less than 0.4 from the plate median for 95% of SOMAmers. All quality metrics for the proteomic assay were met with plate scale factors of 1.24 and 1.46, and SOMAmer calibration factors < 0.4 for 95% of SOMAmers. The median coefficient of variation was 4.1%. A negligible number of proteins did not pass quality control (Fig S17).

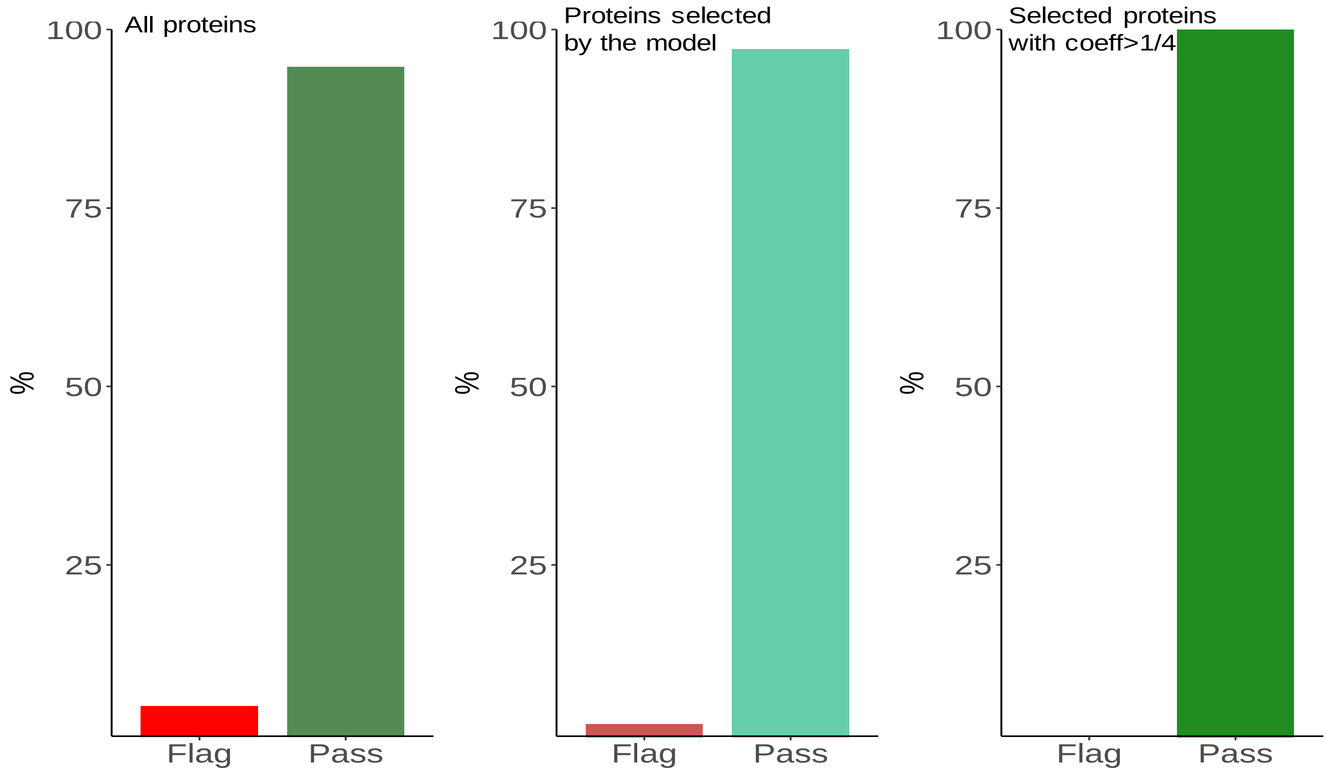

Figure S17. Quality analysis of proteome. Y-axis shows the percentage of proteins that passed the quality assessment.

We point out that SomaLogic aptamer technology used in this study have been previously extensively validated using orthogonal technologies (the enzyme-linked immunosorbent assay (ELISA) and Olink), multiple reaction monitoring mass spectrometry (MRM-MS), data dependent acquisition mass spectrometry (DDA-MS) and genetic strategies. Specifically,

studies that performed validation of proteins identified in our study are listed in Table S5 below.

Table S5. Validation of aptamer assays for identified proteins.			
**Protein**	**Orthogonal strategy**	**MS**	**Genetic Strategies**
LEP	Elisa[21]		
CXCL10	Elisa[22], Olink[23]		[23,24]
SELE	Olink[23]	MRM-MS[24]	[23,24]
SELL	Luminex[25], Olink[23]	DDA-MS[24]	[23,24]
APOB		MRM-MS[24], DDA-DS[24]	[24]
SPARCL1			[23,24]
PRSS2			[23]
ROR1			[24]

3. Microbiome

Self-sampling of the vagina was performed weekly by study participants. Sterile Catch-AllTM Sample Collection Swabs (Epicentre Biotechnologies, Madison, WI, USA) were used to obtain material from: vagina (midvaginal wall). All clinical specimens were placed immediately after collection at −20◦C until transport to the laboratory for storage at −80◦C until further processing. Whole genomic DNA was extracted from each vaginal swab by means of the PowerSoil DNA isolation kit (MO BIO Laboratories) according to the manufacturer's protocol except for the inclusion of a 10-min incubation at 65◦C immediately after the addition of solution C1. The V4 hypervariable region of the 16S rRNA gene was amplified by PCR. The forward PCR primer (50 AAT GAT ACG GCG ACC ACC GAG ATC TAC ACG CTN NNN NNN NNN NNT ATG GTA ATT GTG TGY CAG CMG CCG CGG TAA 30) was a 75-nucleotide (nt) fusion primer consisting of the 32-nt Illumina adapter (designated by bold), a unique 12-nt barcode to label each amplicon (designated by the N's), a 10-nt forward primer pad, a 2-nt linker (GT), and the 19-nt broad-range bacterial primer 515F (designated by underlining). The 56-nt reverse primer (5' CAA GCA GAA GAC GGC ATA CGA GAT AGT CAG CCA GCC GGA CTA

CNV GGG TWT CTA AT 30) consisted of the 24-nt Illumina adapter (designated by bold), a 10-nt reverse primer pad, a 2-nt reverse primer linker (CC), and the 20-nt broad-range bacterial primer 806R (designated by underlining). Triplicate 25-µL PCRs were carried out by using 1× HotMasterMix (5 PRIME), 0.4 µM concentrations of each commercially synthesized primer, and 3 µL of prepared DNA template. Thermal cycling conditions consisted of an initial denaturing step of 94◦C for 3 min, followed by 30 cycles of 94◦C for 45s, 50◦C for 60s, and 72◦C for 90s, with a final extension step of 72◦C for 10 minutes. Upon completion of the PCRs, the corresponding triplicate reaction mixtures were pooled and purified by using the Ultra-clean-htp 96-well PCR clean-up kit (Mo Bio Laboratories) according to the manufacturer's protocol. DNA concentrations from each triplicate pool were quantified using the QuantiT High-Sensitivity dsDNA Assay Kit (Invitrogen) and combined in equimolar
14 ratios into a single tube. The resulting amplicon mixture was concentrated by ethanol precipitation and resuspended in 100 µL of molecular biology-grade water (Life Technologies). The resuspended amplicon mixture was gel purified and recovered using a QIAquick gel extraction kit (Qiagen). Recovered PCR products were sequenced on an Illumina HiSeq 2500 instrument (Illumina) at the W. M. Keck Center for Comparative Functional Genomics at the University of Illinois, Urbana–Champaign, IL. Bioinformatics processing largely followed the DADA2 Workflow for Big Data (benjjneb.github.io/dada2/bigdata_paired.html). Forward/reverse read pairs were trimmed and filtered, with forward reads truncated at 245 nt and reverse reads at 235 nt, no ambiguous bases allowed, and each read required to have less than two expected errors based on their quality scores. The relationship between quality scores and error rates was estimated for each sequencing run to reduce batch effects arising from run-to-run variability. ASVs were

independently inferred from the forward and reverse of each sample using the run-specific error rates, and then read pairs were merged. Chimeras were identified in each sample, and ASVs were removed if identified as chimeric in a sufficient fraction of the samples in which they were present. Taxonomic assignment was performed against the Silva v123 database using the implementation of the RDP naive Bayesian classifier available in the dada2 R package[26]. Lactobacillus species were assigned by hand via BLAST against sequences from cultured Lactobacillus strains.

For the vaginal microbiome dataset, biodiversity coverage was nearly complete, as shown by rarefaction curves for each sample (Fig S18). The curves are asymptotic, suggesting that the sequencing depth was sufficient to exhaustively sample the biodiversity present, which was measured using amplicon sequence variants (ASVs). Note that it is common for vaginal microbiomes to have relatively low estimates of biodiversity in states of health, as shown in Figure S19, and for increased diversity to be associated with disease risk (e.g., preterm birth). Our reads have been submitted to SRA. The BioProject accession is PRJNA752652.

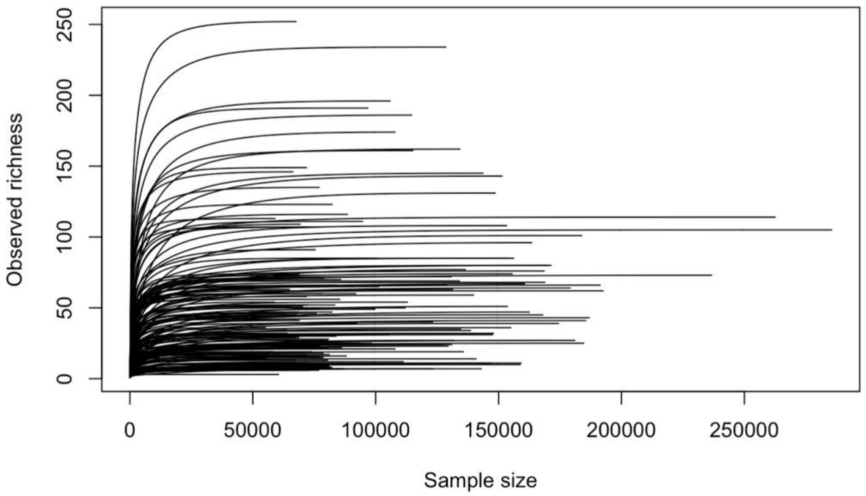

Figure S18. Rarefaction (coverage) curves for vaginal swabs analyzed for microbiome dataset, demonstrating nearly complete biodiversity coverage.

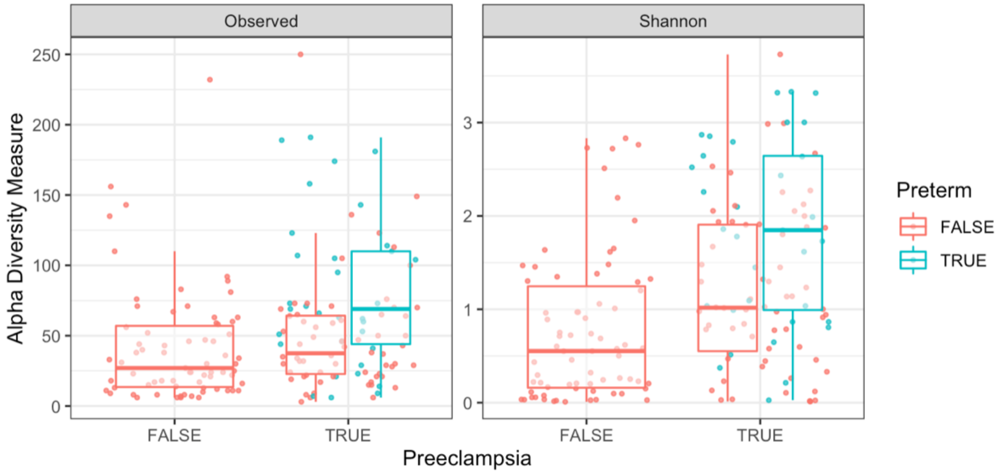

Figure S19. Alpha diversity estimates for vaginal swabs analyzed for microbiome dataset. These demonstrate that vaginal microbiomes have lower estimates of biodiversity in states of health and increased diversity to be associated with disease risk (e.g., preterm birth).

Principal Component Analysis (PCA) revealed that microbiome samples cluster together and no batch effects (Fig. S20).

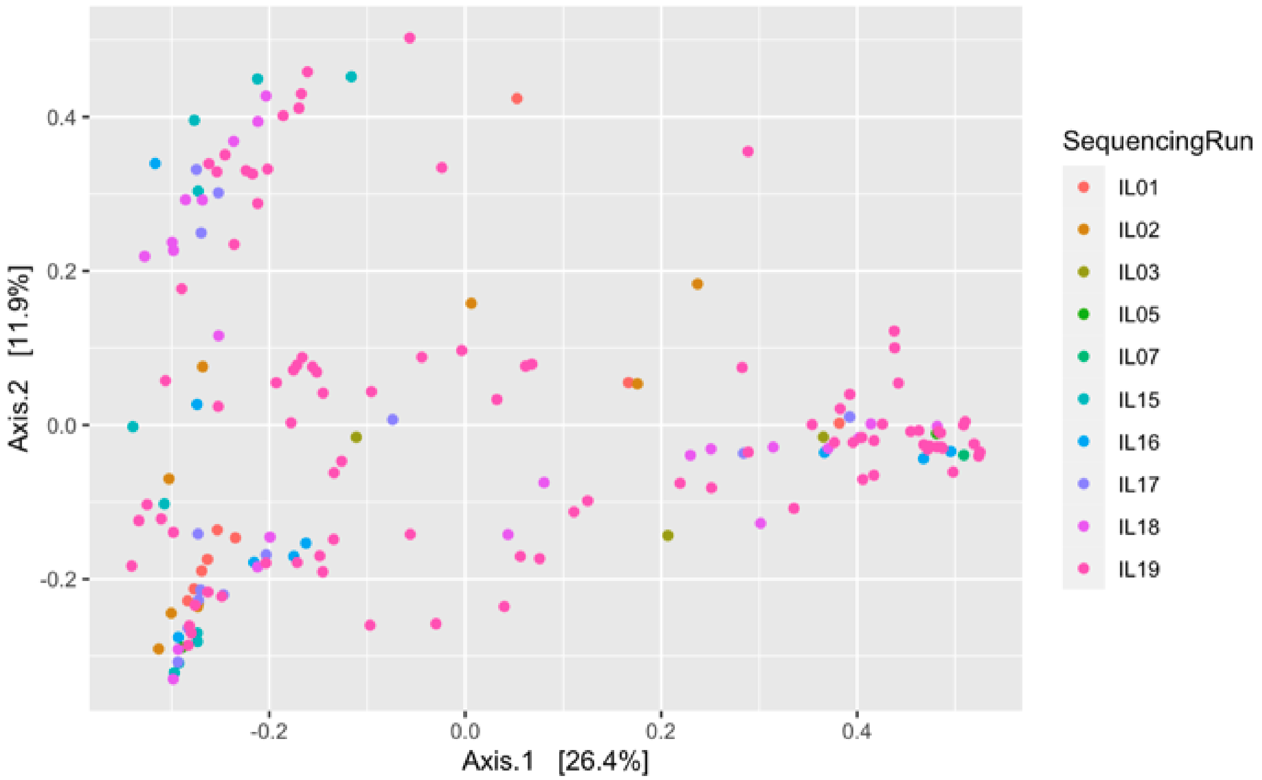

Figure S20: PCA of microbiome samples. Different sequencing runs are shown with different colors.

4. Immunome

Whole blood samples were stimulated for 15 min with either LPS, IFNα, a cocktail containing IL-2 and IL-6, or left unstimulated. Samples were then processed using a standardized protocol for fixation (SmartTube Inc), barcoding and antibody staining of whole blood samples for mass cytometry analysis[27]. For further details see[28]. Three categories of immune features were derived for integrative analysis: Cell frequency features: cell frequencies were expressed as a percentage of gated singlets in the case of neutrophils, and as a percentage of mononuclear cells (CD45+CD66−) in the case of all other cell types. Endogenous signaling immune features: Endogenous intracellular signaling

activities were derived from the analysis of unstimulated blood samples. The signal intensity of the following functional markers was simultaneously quantified per single cell: phospho (p) STAT1, pSTAT3, pSTAT5, pNFκB, total IκB, pMAPKAPK2, pP38, prpS6, pERK1/2, and pCREB. For each cell type, signaling immune features were calculated as the median signal intensity (arcsinh transformed value) of each signaling protein. Intracellular signaling response features: the signal intensity of all functionalmarkers was analyzed from samples stimulated with LPS, IFNα or IL. For each cell type, signaling responses were calculated as the difference in median signal intensity (arcsinh transformed value) of each signaling protein between the stimulated and unstimulated conditions.

5. Metabolomics and Lipidomics Analyses

While lipidome can be considered a part of the metabolome, in this study, we consider them separately because the datasets are generated using a very different workflow. Also, in this study lipidome refers to complex lipids, whereas small lipids such as fatty acids, oxylipins, etc. are part of our metabolome data.

Untargeted Metabolomics by Liquid Chromatography (LC)- Mass Spectrometry (MS)

LC-MS-grade solvents and mobile phase modifiers were obtained from Fisher Scientific (water, acetonitrile, methanol) and Sigma−Aldrich (acetic acid, ammonium acetate). Urine and plasma samples were analyzed using a broad-spectrum metabolomics platform consisting of hydrophilic interaction chromatography (HILIC) and reverse phase liquid chromatography (RPLC)–MS[29].

Sample preparation. Frozen urine samples were thawed on ice and centrifuged at 17,000g for 10 min at 4°C. Supernatants (25 µl) were then diluted 1:4 with 75% acetonitrile and 100% water for

HILIC- and RPLC-MS experiments, respectively. Samples for HILIC-MS experiments were further centrifuged at 21,000g for 10 min at 4°C to precipitate proteins. Frozen plasma samples were thawed on ice and metabolites were prepared from 100 µl of plasma using 1:1:1 acetone:acetonitrile:methanol, evaporated to dryness under nitrogen, and reconstituted in 1:1 methanol:water. Each sample was spiked-in with 15 analytical-grade internal standards (IS).

Data acquisition. Metabolic extracts were analyzed using HILIC and RPLC separations in both positive and negative ionization modes. Data were acquired on a Thermo Q Exactive HF mass spectrometer equipped with a Heated Electrospray Ionization probe (HESI-II) and operating in full MS scan mode. MS/MS data were acquired at different fragmentation energies (NCE 25, 35 and 50) on pooled samples consisting of an equimolar mixture of all the samples in the study. HILIC experiments were performed using a ZIC-HILIC column 2.1 x 100 mm, 3.5 µm, 200Å (Merck Millipore) and mobile phase solvents consisting of 10 mM ammonium acetate in 50/50 acetonitrile/water (A) and 10 mM ammonium acetate in 95/5 acetonitrile/water (B). RPLC experiments were performed using a Zorbax SBaq column 2.1 x 50 mm, 1.7 µm, 100Å (Agilent Technologies) and mobile phase solvents consisting of 0.06% acetic acid in water (A) and 0.06% acetic acid in methanol (B).

Data quality was ensured by: (1) sample randomization for metabolite extraction and data acquisition, (2) multiple injections of a pooled sample to equilibrate the LC-MS system prior to running the sequence (12 and 6 injections for HILIC and RPLC methods, respectively), (3) spike-in labeled IS during sample preparation to control for extraction efficiency and evaluate LC-MS performance, (4) checking mass accuracy, retention time and peak shape of the IS in each sample and (5) injection of a pooled sample every 10 injections to control for signal deviation over time.

Data processing. Data from each mode were independently processed using Progenesis QI software (v2.3) (Nonlinear Dynamics). Metabolic features from blanks and that did not show sufficient linearity upon dilution in QC samples ($r < 0.6$) were discarded. Only metabolic features present in > 2/3 of the samples were kept for further analysis. Inter- and intra-batch variations were corrected by applying locally estimated scatterplot smoothing local regression (LOESS) on pooled samples injected repetitively along the batches (span = 0.75). Dilution effects for urine samples were corrected using probabilistic quotient normalization (PQN). Missing values were imputed by drawing from a random distribution of low values in the corresponding sample. Data from each mode were then merged, producing a dataset containing 8718 and 3622 metabolic features for urine and plasma, respectively. Metabolite abundances were reported as spectral counts.

Metabolic feature annotation. Peak annotation was first performed by matching experimental m/z, retention time and MS/MS spectra to an in-house library of analytical-grade standards. Remaining peaks were identified by matching experimental m/z and fragmentation spectra to publicly available databases including HMDB (http://www.hmdb.ca/), MoNA (http://mona.fiehnlab.ucdavis.edu/) and MassBank (http://www.massbank.jp/) using the R package 'metID' (v0.2.0)[30]. Briefly, metabolic feature tables from Progenesis QI were matched to fragmentation spectra with a m/z and a retention time window of ± 15 ppm and ± 30 s (HILIC) and ± 20 s (RPLC), respectively. When multiple MS/MS spectra match a single metabolic feature, all matched MS/MS spectra were used for the identification. Next, MS1 and MS2 pairs were searched against public databases and a similarity score was calculated using the forward dot–product algorithm which considers both fragments and intensities. Metabolites were reported if the similarity score was above 0.4. We used the Metabolomics Standards Initiative (MSI) level of

confidence to grade metabolite annotation confidence (level 1 - level 4). Level 1 represents formal identifications where the biological signal matches accurate mass, retention time and fragmentation spectra of an authentic standard run on the same platform. For level 2 identification, the biological signal matches accurate mass and fragmentation spectra available in one of the public databases listed above. Level 3 represents putative identifications that are the most likely name based on previous knowledge of blood and urine composition. Level 4 consists in unknown metabolites. Annotated urine metabolites selected in the prediction models are reported in Table S3.

PCA revealed that samples cluster together and the absence of batch effects (Fig. S21), thereby confirming satisfactory quality control.

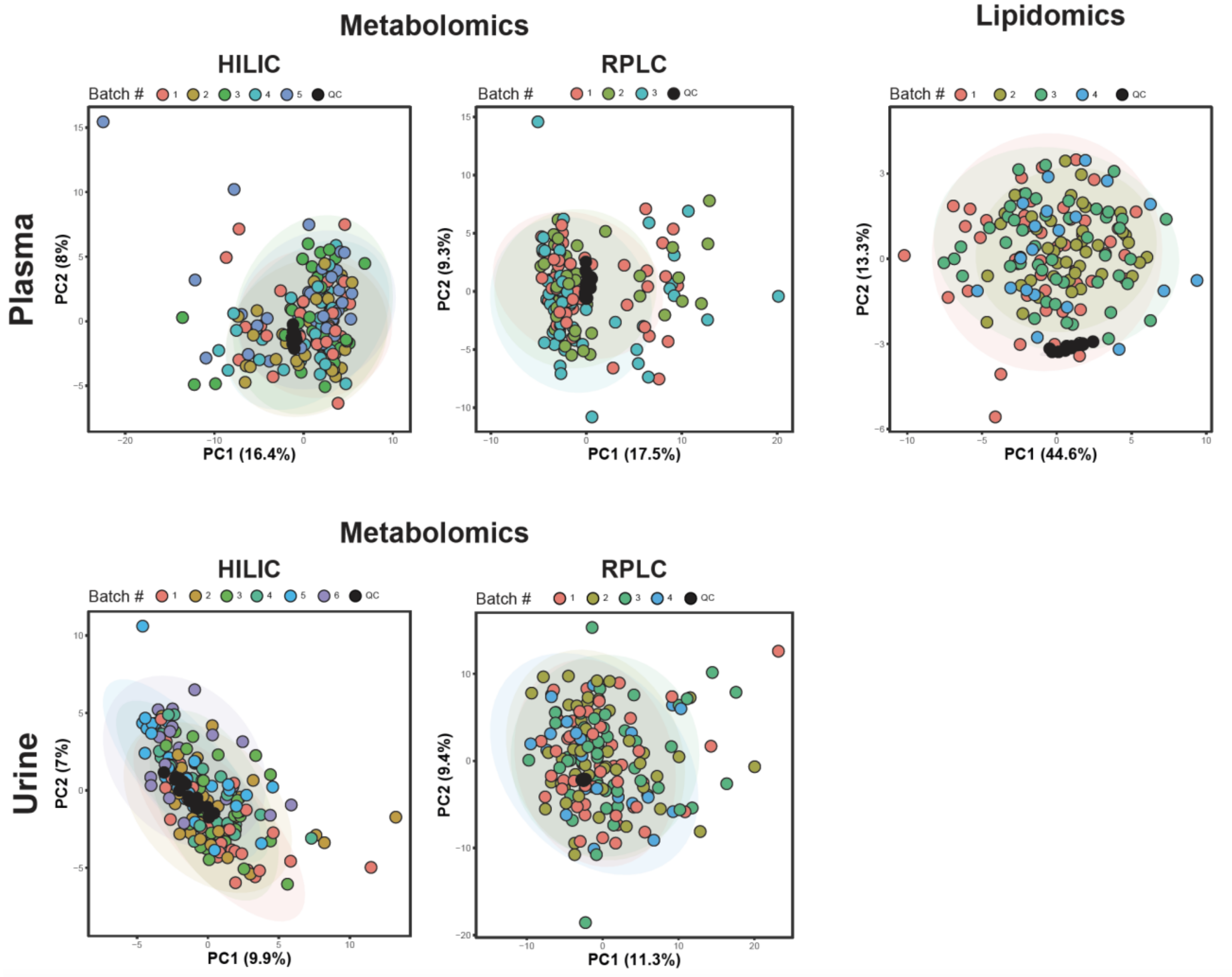

Figure S21: PCA of metabolome plasma, metabolome urine and lipidome samples. Different colors are assigned to different batches.

Comparison between enrichment factors and statistical significance of pathways enriched over gestation versus in early pregnancy is shown in Figure S22.

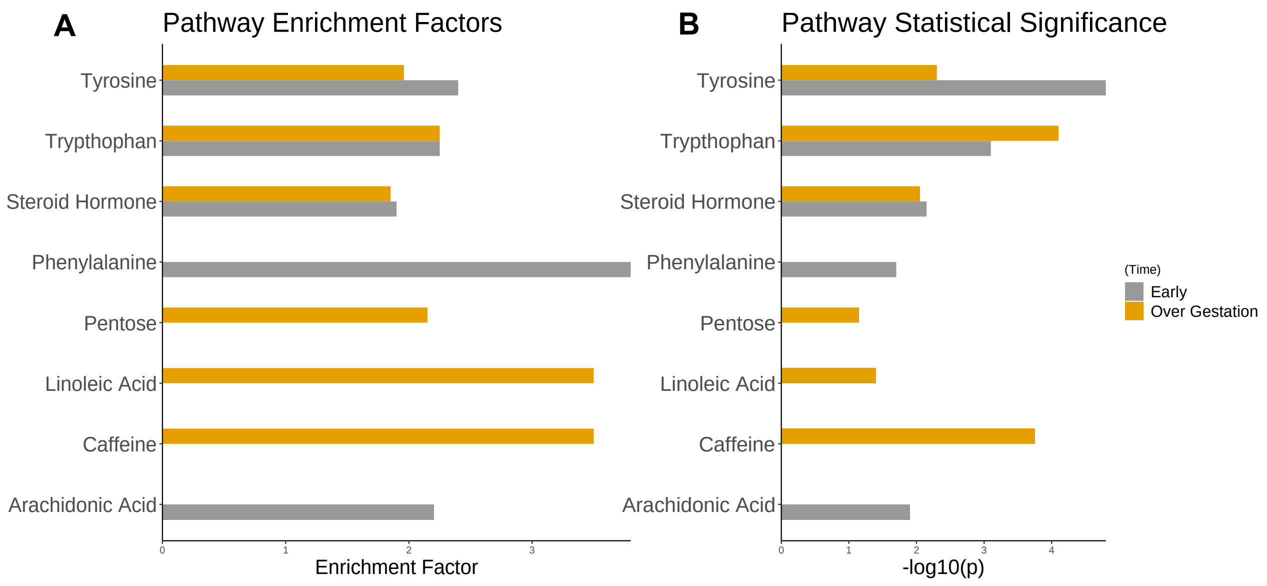

Figure S22. Pathways enriched over gestation (yellow) and early in pregnancy (grey). A. Pathway enrichment. **B.** Statistical significance.

References

1. Taylor BD, Ness RB, Olsen J, Hougaard DM, Skogstrand K, Roberts JM, Haggerty CL. leptin measured in early pregnancy is higher in women with preeclampsia compared with normotensive pregnant women. Hypertension 65, 594–599 (2015).
2. Pérez-Pérez A, Toro A, Vilariño-García T, Maymó J, Guadix P, Dueñas JL, Fernández-Sánchez M, Varone C, Sánchez-Margalet V.Leptin action in normal and pathological pregnancies. J. Cell Mol. Med. 22, 716–727 (2018).
3. Naylor, C. & Petri, W. A. Leptin regulation of immune responses. Trends Mol. Med. 22, 88–98 (2016).
4. Abella V, Scotece M, Conde J, Pino J, Gonzalez-Gay MA, Gómez-Reino JJ, Mera A, Lago F, Gómez R, Gualillo O. Leptin in the interplay of inflammation, metabolism and immune system disorders. Nat. Rev. Rheumatol. 13, 100–109 (2017).
5. Martín-Romero, C., Santos-Alvarez, J., Goberna, R. & Sánchez-Margalet, V. Human leptin enhances activation and proliferation of human circulating T lymphocytes. Cell Immunol. 199, 15–24 (2000).
6. Maynard, S. E. & Karumanchi, S. A. Angiogenic factors and preeclampsia. Semin Nephrol 31, 33–46 (2011).
7. Rath, G. & Tripathi, R. Angiogenic balance and diagnosis of pre-eclampsia: selecting the right VEGF receptor. J Hum Hypertens 26, 207–210 (2012).
8. Docheva N, Romero R, Chaemsaithong P, Tarca AL, Bhatti G, Pacora P, Panaitescu B, Chaiyasit N, Chaiworapongsa T, Maymon E, Hassan SS, Erez O.The profiles of soluble adhesion molecules in the “great obstetrical syndromes”. J. Matern. Fetal Neonatal Med. 32, 2113–2136 (2019).
9. Ivetic, A., Hoskins Green, H. L. & Hart, S. J. L-selectin: A Major Regulator of Leukocyte Adhesion, Migration and Signaling. Front. Immunol. 10, 1068 (2019).
10. Seidelin, J. B., Nielsen, O. H. & Strøm, J. Soluble L-selectin levels predict survival in sepsis. Intensive Care Med. 28, 1613–1618 (2002).
11. Rainer, T. H. L-selectin in health and disease. Resuscitation 52, 127–141 (2002).
12. Chen J, Yue C, Xu J, Zhan Y, Zhao H, Li Y, Ye Y. Downregulation of receptor tyrosine kinase-like orphan receptor 1 in preeclampsia placenta inhibits human trophoblast cell proliferation, migration, and invasion by PI3K/AKT/mTOR pathway accommodation. Placenta 82, 17–24 (2019).
13. Gotsch F, Romero R, Friel L, Kusanovic JP, Espinoza J, Erez O, Than NG, Mittal P, Edwin S, Yoon BH, . et al. CXCL10/IP-10: a missing link between inflammation and anti-angiogenesis in preeclampsia? J. Matern. Fetal Neonatal Med. 20, 777–792 (2007).
14. Løset M, Mundal SB, Johnson MP, Fenstad MH, Freed KA, Lian IA, Eide IP, Bjørge L, Blangero J, Moses EK, Austgulen R. A transcriptional profile of the decidua in preeclampsia. Am. J. Obstet. Gynecol. 204, 84.e1-27 (2011).
15. Ma, H. Y., Cu, W., Sun, Y. H. & Chen, X. MiRNA-203a-3p inhibits inflammatory response in preeclampsia through regulating IL24. Eur Rev Med Pharmacol Sci 24, 5223–5230 (2020).
16. Zhang, Y., Cao L., Jia J., Ye L., Wang Y., Zhou B., Zhou R. CircHIPK3 is decreased in preeclampsia and affects migration, invasion, proliferation, and tube formation of human trophoblast cells. Placenta 85, 1–8 (2019).
17. Moufarrej M.N., Vorperian S.K., Wong R.J., Campos A.A., Quaintance C.C., Sit R.V., Tan M., Detweiler A.M., Mekonen H., Neff N.F., Baruch-Gravett C., Litch J.A., Druzin M.L., Winn V.D.,

Shaw G.M., Stevenson D.K., Quake S.R. Early prediction of preeclampsia in pregnancy with cell-free RNA. Nature. 2022 Feb;602(7898):689-694. doi: 10.1038/s41586-022-04410-z. Epub 2022 Feb 9. PMID: 35140405; PMCID: PMC8971130.
18. Pan W., Development of diagnostic methods using cell-free nucleic acids. Stanford University, 2016.
19. Moufarrej M., Wong R.J., Shaw G.M., Stevenson D.K., Quake S.R. Investigating Pregnancy and Its Complications Using Circulating Cell-Free RNA in Women's Blood During Gestation, Front. Pediatr., 2020.
20. Gold, L., Ayers D., Bertino J., Bock C., Bock A., Brody E.N., Carter J., Dalby A.B., Eaton B.E., Fitzwater T., *et al.* Aptamer-based multiplexed proteomic technology for biomarker discovery. *PLoS One* **5,** e15004 (2010).
21. Anderson, J., Seol H., Gordish-Dressman H., Hathout Y., Spurney C.F. Interleukin 1 Receptor-Like 1 Protein (ST2) is a Potential Biomarker for Cardiomyopathy in Duchenne Muscular Dystrophy. *Pediatr. Cardiol.* **38,** 1606–1612 (2017).
22. Bodewes, I. L. A., Van der Spek P.J., Leon L.G., Wijkhuijs A.J.M., Van Helden-Meeuwsen C.G., Tas L., Schreurs M.W.J., Van Daele P.L.A., Katsikis P.D., Versnel M.A. Fatigue in Sjögren's syndrome: A search for biomarkers and treatment targets. *Front. Immunol.* **10,** (2019).
23. Sun, B. B., Maranville J.C., Peters J.E., Stacey D., Staley J.R., Blackshaw J., Burgess S., Jiang T., Paige E., Surendran P., *et al.* Genomic atlas of the human plasma proteome. *Nature* **558,** 73–79 (2018).
24. Emilsson, V., Ilkov M., Lamb J.R., Finkel N., Gudmundsson E.F., Pitts R., Hoover H., Gudmundsdottir V., Horman S.R., Aspelund T. *et al.* Co-regulatory networks of human serum proteins link genetics to disease. *Science* **361,** 769–773 (2018).
25. Giudice, V., Biancotto A., Wu Z., Cheung F., Candia J., Fantoni G., Kajigaya S., Rios O., Townsley D., Feng X., Young N.S. Aptamer-based proteomics of serum and plasma in acquired aplastic anemia. *Exp. Hematol.* **68,** 38–50 (2018).
26. Wang Q., Garrity G.M., Tiedje J.M., Cole J.R. Naive Bayesian classifier for rapid assignment of rRNA sequences into the new bacterial taxonomy. Appl Environ Microbiol. 2007 Aug;73(16):5261-7. doi: 10.1128/AEM.00062-07. Epub 2007 Jun 22. PMID: 17586664; PMCID: PMC1950982.
27. Bodenmiller, B., Zunder, E., Finck, R., Chen T.J., Savig E.S., Bruggner R.V., Simonds E.F., Bendall S.C., Sachs K., Krutzik P.O., Nolan G.P. Multiplexed mass cytometry profiling of cellular states perturbed by small-molecule regulators. *Nat Biotechnol* **30**, 858–867 (2012). https://doi.org/10.1038/nbt.2317
28. Aghaeepour N., Ganio EA, Mcilwain D., Tsai A.S., Tingle M., Van Gassen S., Gaudilliere D.K., Baca Q., McNeil L., Okada R., Ghaemi M.S., Furman D., Wong R.J., Winn V.D., Druzin M.L., El-Sayed Y.Y. *et.al.*, B. An immune clock of human pregnancy. Sci Immunol. 2017 Sep 1;2(15):eaan2946. doi: 10.1126/sciimmunol.aan2946. PMID: 28864494; PMCID: PMC5701281.
29. Contrepois K., Jiang L., Snyder M. Optimized Analytical Procedures for the Untargeted Metabolomic Profiling of Human Urine and Plasma by Combining Hydrophilic Interaction (HILIC) and Reverse-Phase Liquid Chromatography (RPLC)-Mass Spectrometry. Mol Cell Proteomics. 2015 Jun;14(6):1684-95. doi: 10.1074/mcp.M114.046508. Epub 2015 Mar 18. PMID: 25787789; PMCID: PMC4458729.
30. Shen, X., Wang R., Xiong X., Yin Y., Cai Y., Ma Z., Liu N., Zhu Z-J. Metabolic reaction network-based recursive metabolite annotation for untargeted metabolomics. *Nat. Commun.* **10,**

1516 (2019).
